# New Tricyclic Aryl Quinazoline Derivatives by Suzuki‐Miyaura Cross‐Coupling

**DOI:** 10.1002/open.202400197

**Published:** 2024-09-27

**Authors:** Burkhon Elmuradov, Rasul Okmanov, Bakhromjon Juraev, Gerald Dräger, Holger Butenschön

**Affiliations:** ^1^ Leibniz Universität Hannover Institut für Organische Chemie Schneiderberg 1B D-30167 Hannover Germany; ^2^ Institute of the Chemistry of Plant Substances Academy of Sciences of Uzbekistan 100170 Mirzo-Ulugbek str. 77 Tashkent Uzbekistan

**Keywords:** Cross-Coupling, Palladium Catalysis, Suzuki-Miyaura Reaction, Quinazoline

## Abstract

A number of new deoxyvasicinone (2,3‐dihydropyrrolo[2,1‐*b*]quinazolin‐9(1*H*)‐one) and mackinazolinone (6,7,8,9‐tetrahydro‐11*H*‐pyrido[2,1‐*b*]quinazolin‐11‐one) derivatives with aryl substituents at C7/C8 and at C5 are reported. These compounds are rare representatives of their kind and were prepared in high yields by Suzuki‐Miyaura cross‐coupling reactions between 7‐bromo‐2,3‐dihydro[2,1‐*b*]quinazoline‐9‐(1*H*)*‐*one, 5,7‐dibromo‐2,3‐dihydro[2,1‐*b*]quinazoline‐9‐(1*H*)*‐*one or 8‐bromomackinazolinone and respective arylboronic acids with palladium acetate as the catalyst. The products were characterized spectroscopically and, in addition, by X‐ray crystal structure analyses in six cases.

## Graphical abstract

New quinazoline derivatives are prepared in high yields by single or double Suzuki‐Miyaura cross‐coupling of the rare respective brominated precursors. Six of the products are crystallographically characterized, and results of some DFT calculations of the new products are reported.

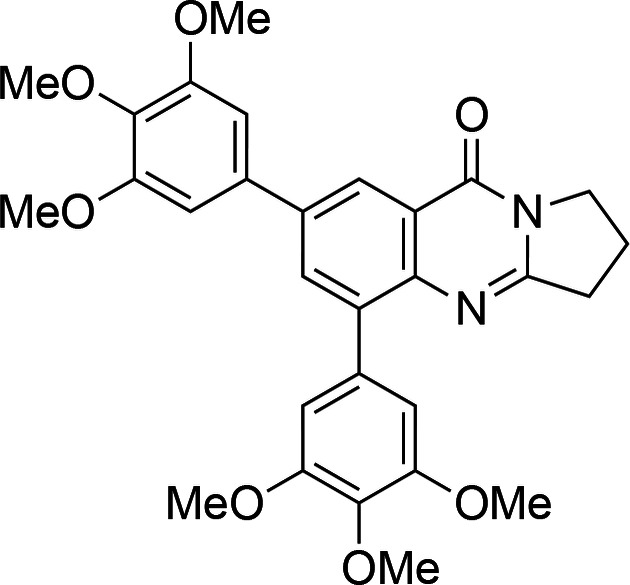

## Introduction

**Scheme 1:**
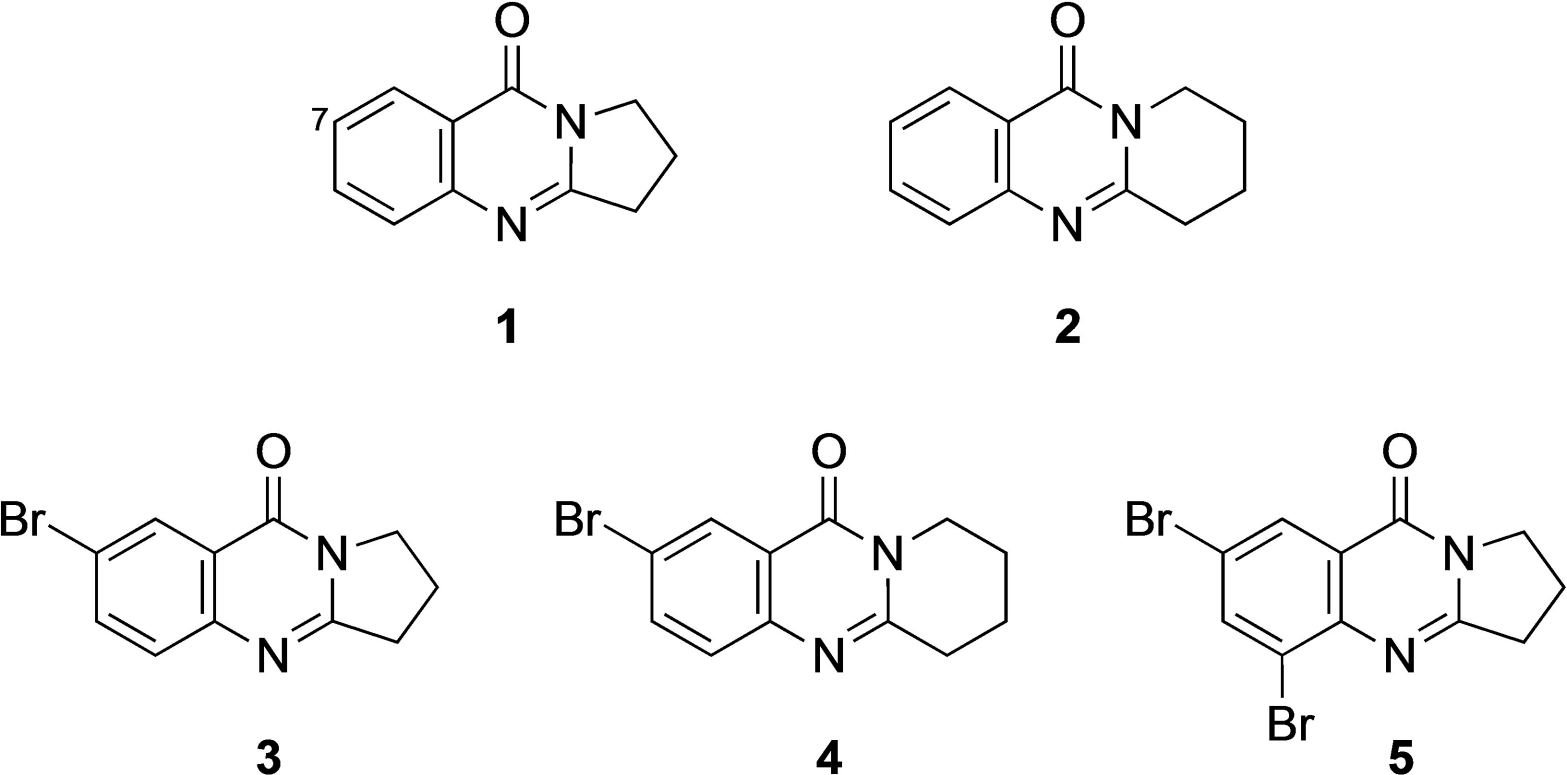
Deoxyvasicinone (**1**), mackinazolinone (**2**), and bromo derivatives **3**–**5**.

Deoxyvasicinone (2,3‐dihydropyrrolo[2,1‐*b*]quinazolin‐9(1*H*)‐one, **1**)^[1]^ and mackinazolinone (6,7,8,9‐tetrahydro‐11*H*‐pyrido[2,1‐*b*]quinazolin‐11‐one, **2**)^[2]^ are tricyclic quinazoline alkaloids with significant anti‐inflammatory, anti‐microbial, anti‐depressant and anti‐oxidant activities,[^3^, ^4^, ^5^, ^6^, ^7^] which also deserve interest as ligands in the context of the treatment of Alzheimer's disease.^[8]^ Therefore a number of derivatives have been prepared with a variety of substitution patterns.[^9^, ^10^] Interestingly, there are less than 80 derivatives of **1** known, which bear an aryl substituent at C‐7 (Scheme 1).^[11]^

Remarkably, reports of the bromo derivatives **3**,[^1^, ^12^, ^13^, ^14^, ^15^, ^16^, ^17^, ^18^, ^19^, ^20^, ^21^, ^22^] **4**,[^17^, ^23^] and **5**[^21^, ^24^] are comparatively rare. However, they offer the attractive possibility to obtain a variety of new derivatives by using palladium‐catalyzed coupling reactions such as the Suzuki‐Miyaura cross‐coupling.[^25^, ^26^, ^27^, ^28^] In the context of our ongoing research[^10^, ^16^, ^29^, ^30^, ^31^, ^32^, ^33^] we investigated this possibility and report here the syntheses and characterization of a number of new tricyclic quinazoline derivatives starting from **3**–**5**. **3**–**5** were obtained by condensation of 5‐bromoanthranilic acid or 3,5‐dibromoanthranilic acid with respective lactams according to the published procedures (see Experimental Section).[^17^, ^19^, ^34^]

## Results and Discussion

Biaryl derivatives have a remarkable importance in a variety of fields such as natural products synthesis, medicinal chemistry or materials science.[^35^, ^36^, ^37^, ^38^] While stoichiometric reactions targeting at axially chiral derivatives[^39^, ^40^] and dehydrogenative couplings such as the Scholl reaction^[41]^ are still important, over the last decades catalytic reactions allowing for the coupling of two aryl building blocks have been developed,[^42^, ^43^, ^44^, ^45^] the most common ones being Suzuki‐Miyaura,[^46^, ^47^, ^48^] Negishi, and Stille coupling reactions.^[27]^ Among these the Suzuki‐Miyaura coupling often gives highest yields and, in addition, has the advantage of using the least toxic, often commercially available and most easily to handle reagents.

**Scheme 2:**
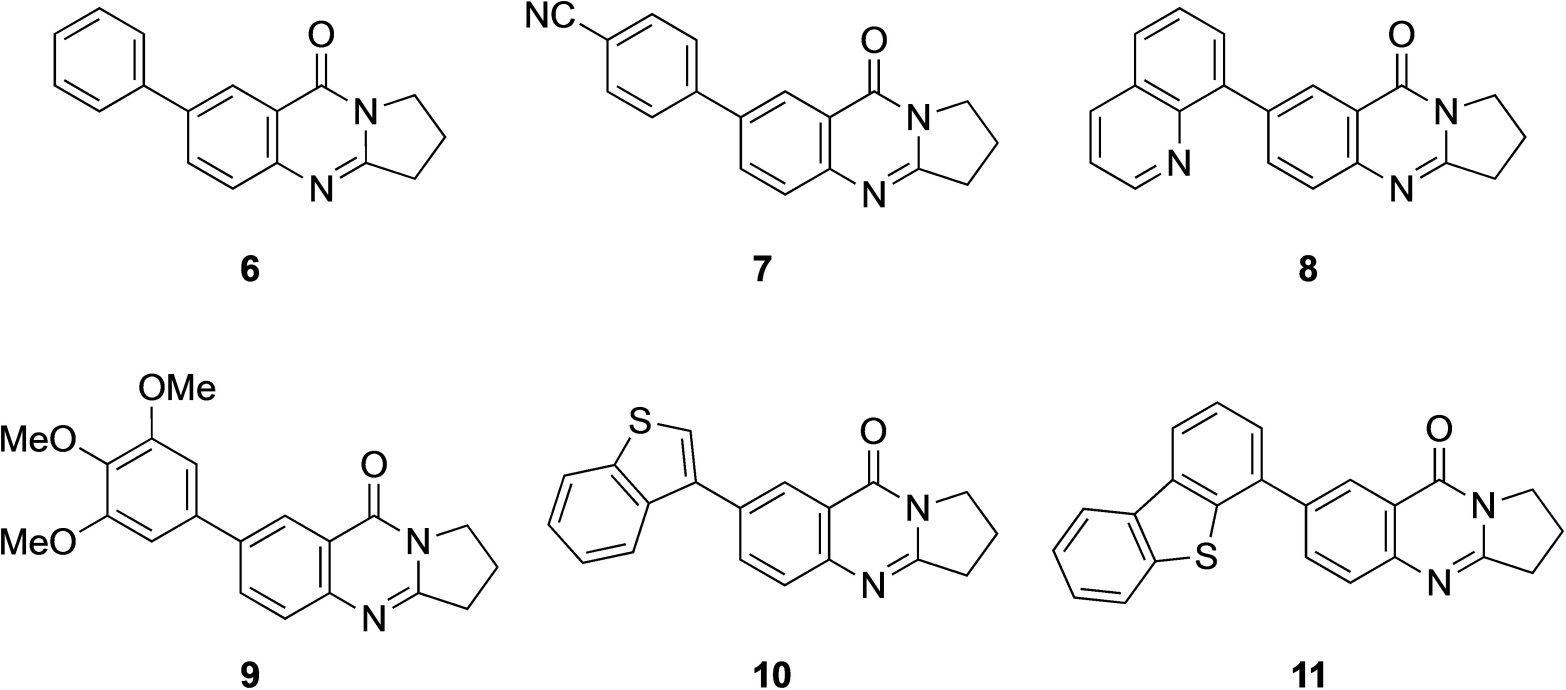
Arylated deoxyvasicinone derivatives **6**–**11**.

New deoxyvasicinone derivatives **6**–**11** (Scheme 2) were prepared in high yields by palladium‐catalyzed Suzuki‐Miyaura coupling of **3** (Table 1) with the respective boronic acids. Use of Pd(OAc)_2_ gave 10–15 % higher yields than reactions performed with [Pd(PPh_3_)_4_] as the catalyst. Smaller catalyst amounts resulted in reduced product yields.

**Table 1:** Suzuki‐Miyaura coupling reactions of 7‐bromo‐2,3‐dihydropyrrolo[2,1‐b]quinazoline‐9‐(1H)‐one (**3**).

Entry	Product	Conditions	Yield (%)
1	**6**	Pd(OAc)_2_ (4.2 mol %), PhB(OH)_2_ (2.2 equiv.), Na_2_CO_3_ (2.0 equiv.), acetone/water (1 : 1), 40–45 °C, 3 h.	92
3	**7**	Pd(OAc)_2_ (4.2 mol %), 4‐NCC_6_H_4_B(OH)_2_ (1.1 equiv.), Na_2_CO_3_ (2.5 equiv.), acetone/water (2 : 1), 40–45 °C, 3 h.	96
4	**8**	Pd(OAc)_2_ (4.2 mol %), 8‐quinolinyl B(OH)_2_, (1.2 equiv.), Na_2_CO_3_ (2.5 equiv.), acetone/water (2 : 1), 40–45 °C, 5 h.	83
5	**9**	Pd(OAc)_2_ (4.2 mol %), (3,4,5‐trimethoxyphenyl) B(OH)_2_ (1.2 equiv.), Na_2_CO_3_ (2.5 equiv.), acetone/water (2 : 1), 40–45 °C, 0.5 h.	97
6	**10**	Pd(OAc)_2_ (4.2 mol %), benzothiophene‐3‐B(OH)_2_ (2.2 equiv.), Na_2_CO_3_ (2.0 equiv.), acetone/water (2 : 1), 40–45 °C, 3 h.	89
7	**11**	Pd(OAc)_2_ (4.2 mol %), dibenzothiophene‐4‐B(OH)_2_ (1.2 equiv.), Na_2_CO_3_ (2.5 equiv.), acetone/water (2 : 1), 40–45 °C, 7 h.	91


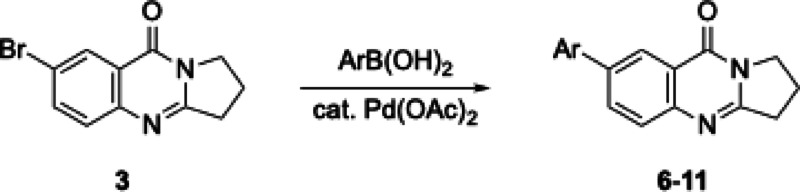


**Figure 1:**
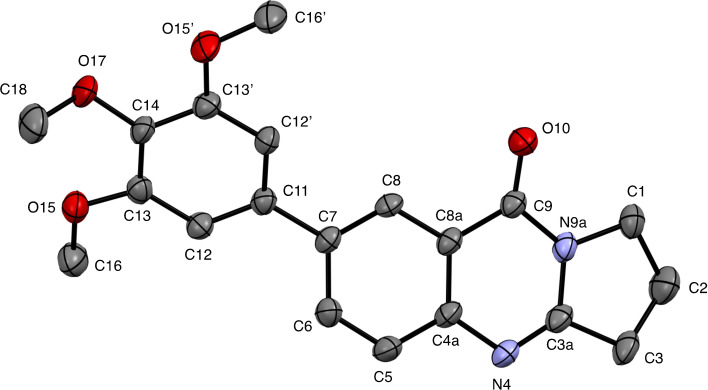
Structure of **9** in the crystal.^[49]^ Ellipsoids at 50 % probability level. Hydrogen atoms omitted for clarity. For selected bond lengths, bond angles and torsional angles see Table 3.

**Figure 2:**
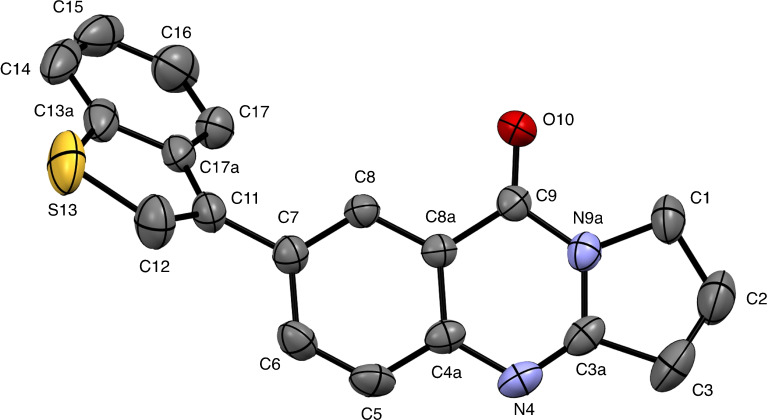
Structure of **10** in the crystal.^[49]^ Ellipsoids at 50 % probability level. For selected bond lengths, bond angles and torsional angles see Table 3.

**6**–**11** were characterized on the basis of their consistent spectroscopic data (^1^H NMR, ^13^C{^1^H} NMR, IR, MS). In addition, compounds **9** and **10** were subjected to X‐ray crystal structure analyses (Figures 1, 2).

Most of the reactions give the π extended reaction products in very high yields. However, use of 2,4,6‐trifluorophenyl boronic acid under the usual conditions did not result in product formation, presumably because of the electron withdrawing effect of the fluoro substituents in *ortho* and *para* positions.

**Scheme 3:**
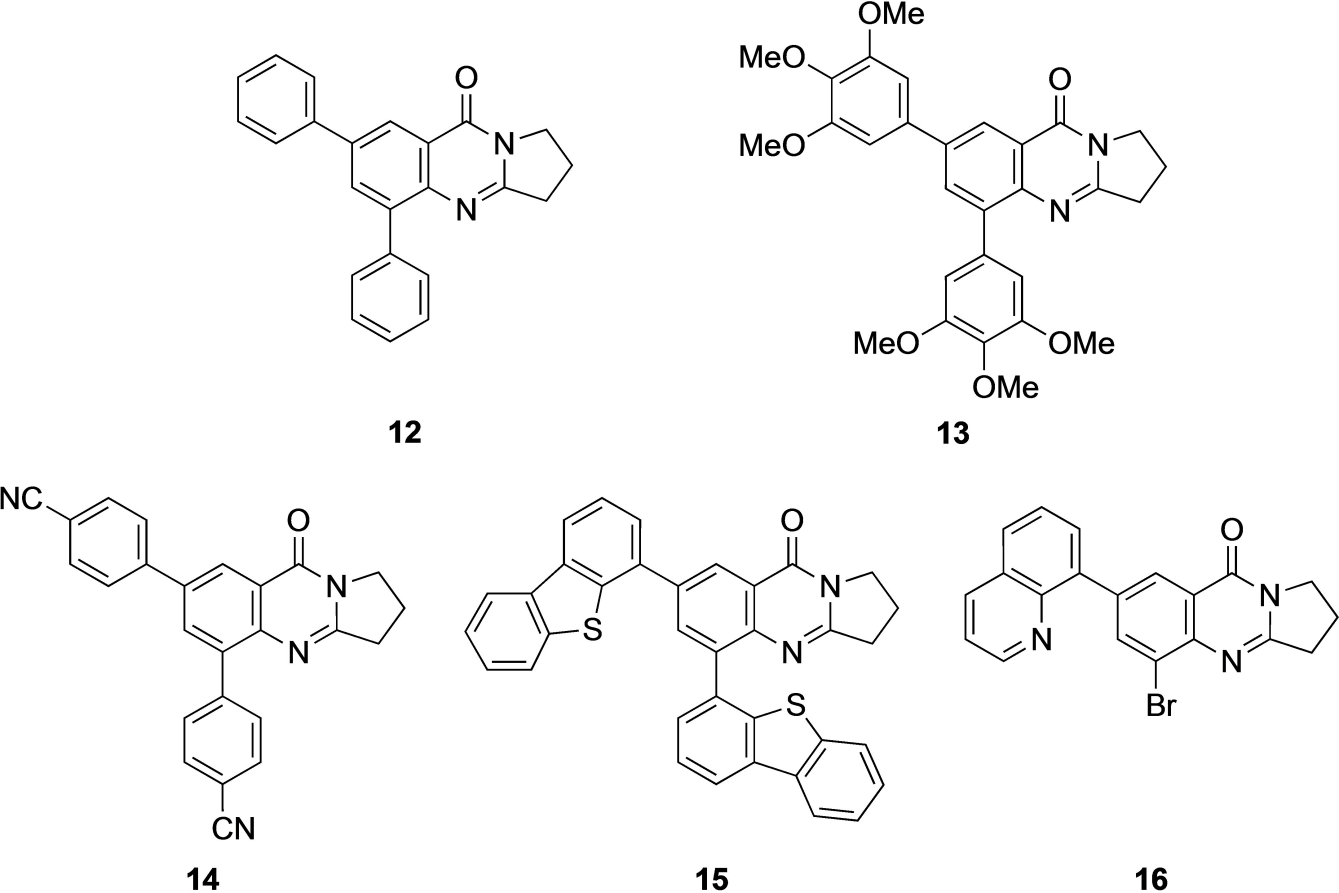
Arylated deoxyvasicinone derivatives **12**–**16**.

5,7‐Dibromo derivative **5** offers the possibility of a double cross‐coupling reaction. This was tested and gave disubstituted derivatives **12**–**16** in 74–98 % yield (Table 2, Scheme 3). Remarkably, the reaction with 8‐quinolineboronic acid remained incomplete and gave mono‐substituted derivative **16** in 68 % yield clearly indicating that the Suzuki‐Miyaura coupling at C7 is significantly faster than that at C5 with the additional option of attaching different substituents at C5 and C7. This may be a result of the proximity of the nitrogen atom N4, which bears a lone electron pair possibly interacting with the catalyst metal. 5,7‐Diarylated 2,3‐dihydro[2,1‐*b*]quinazoline‐9‐(1*H*)‐one derivatives are very rare, a SciFinder® substructure search gave only five loosely related compounds mentioned in a Chinese patent in the context of electroluminescence applications.^[50]^

**Table 2:** Suzuki‐Miyaura coupling of 5,7‐dibromo‐2,3‐dihydro[2,1‐*b*]quinazoline‐9‐(1*H*)*‐*one (**5**). Conditions: Pd(OAc)_2_ (8.4 mol %), ArB(OH)_2_ (2.4 equiv.), Na_2_CO_3_ (2.5 mmol), acetone/water (1 : 1), 40–45 °C, 5 h.

Entry	Product	Yield (%)
1	**12**	84
3	**13**	97
4	**14**	98
5	**15**	75
6	**16**	68

**Figure 3:**
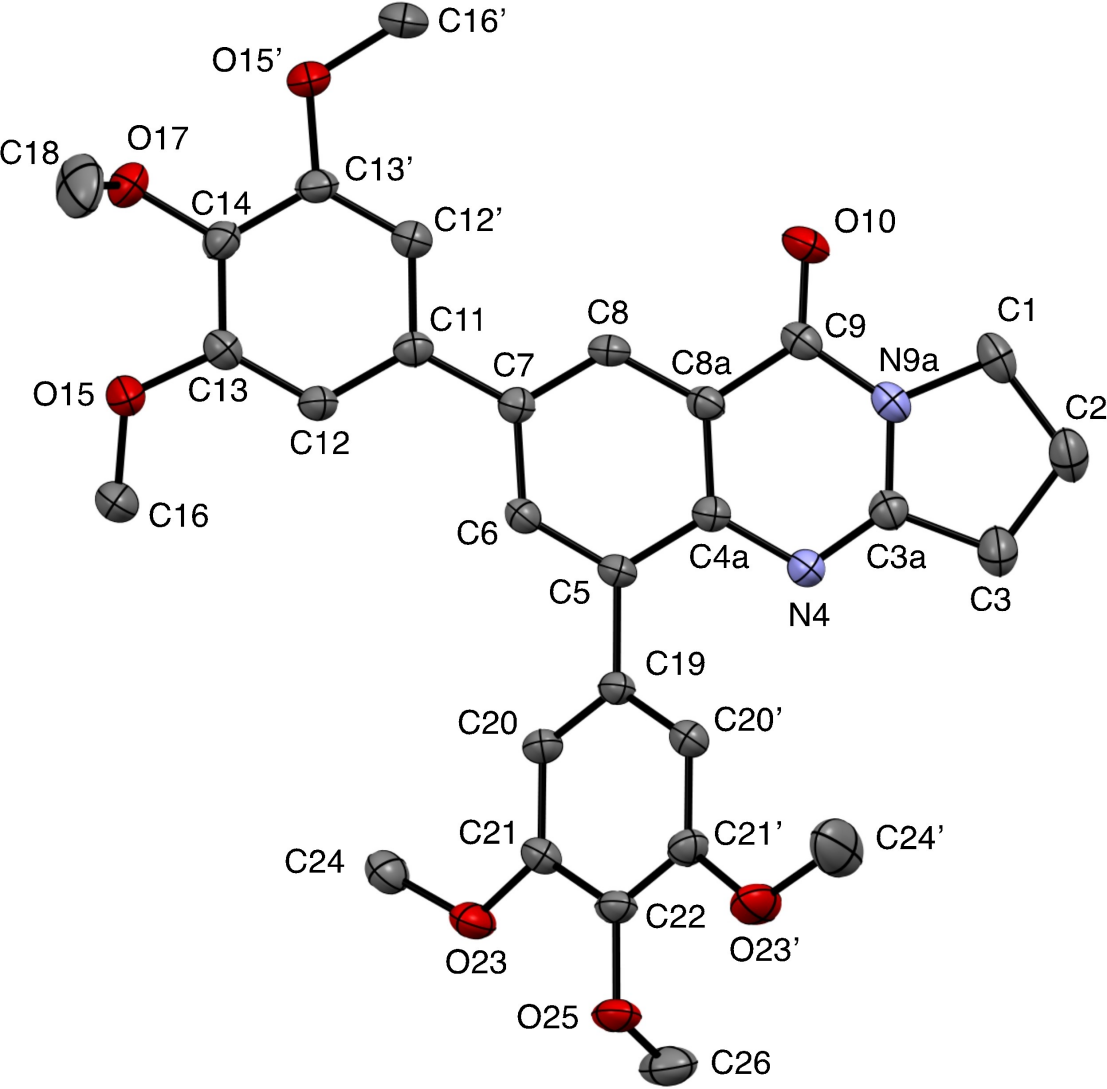
Structure of **13** in the crystal.^[49]^ Ellipsoids at 50 % probability level. Hydrogen atoms omitted for clarity. For selected bond lengths, bond angles and torsional angles see Table 3.

**Figure 4:**
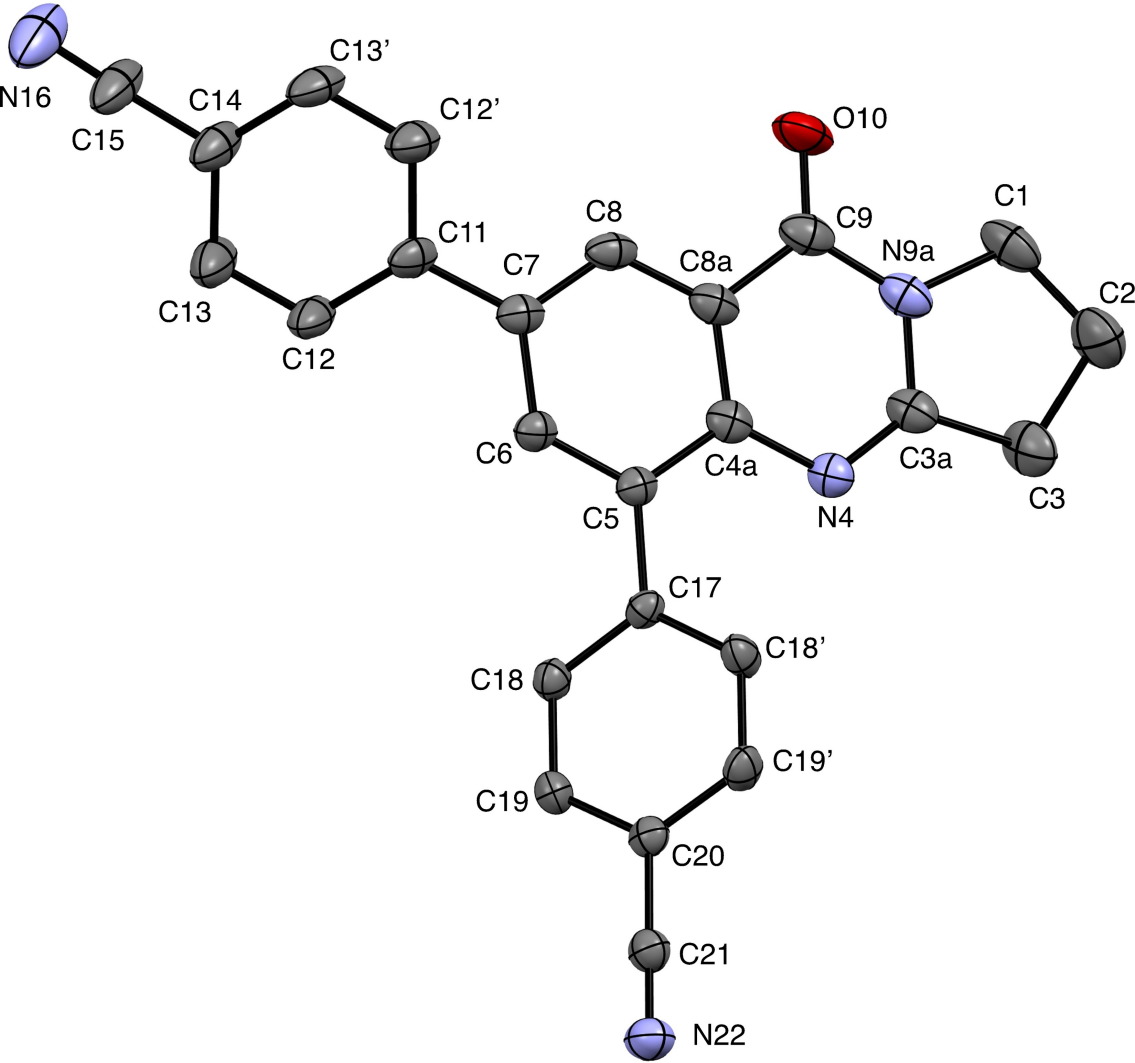
Structure of **14** in the crystal.^[49]^ Ellipsoids at 50 % probability level. Hydrogen atoms omitted for clarity. For selected bond lengths, bond angles and torsional angles see Table 3.

Coupling products **12**–**16** were characterized spectroscopically, and crystals suitable for X‐ray structure analyses were obtained from **13** and **14** (Figures 3 and 4).

Selected geometrical data of coupling products **9**, **10**, **13**, and **14** are listed in Table 3. Within the systematic error the data in Table 3 indicate same geometries for the basic tricyclic quinazoline systems in **9**, **10**, **13**, and **14**. Slight deviations can be observed at the substituted positions. Differences are observed for the torsional angles indicating the degree of non‐coplanarity of up to 48.3° of the π systems of the basic system and that of the substituents, particularly reflecting the steric bulk of the substituents in **10** and **13**.

**Table 3:** Selected bond lengths, bond angles, and torsion angles of deoxyvasicinone derivatives **9**, **10**, **13**, and **14** as determined by X‐ray crystal structure analyses.^[49]^ For atom numbering schemes see Figures 1, 2, 3, 4.

Bond length [pm], bond angle [°] or torsion angle [°]	**9**	**10**	**13**	**14**
C1‐C2	152.9(4)	151.5(3)	153.9(3)	153.2(5)
C1‐N9a	146.7(3)	147.6(3)	147.3(3)	147.7(4)
C2‐C3	151.9(4)	150.8(4)	152.4(4)	154.1(5)
C3‐C3a	150.0(4)	150.4(3)	151.0(3)	150.1(5)
C3a‐N4	128.9(3)	128.4(3)	129.6(3)	129.4(3)
C3a‐N9a	137.7(3)	137.8(2)	137.3(3)	137.5(4)
N4‐C4a	139.8(3)	139.4(2)	140.6(3)	139.5(3)
C4a‐C5	139.5(4)	140.2(2)	141.9(3)	143.7(3)
C5‐C6	137.6(3)	136.9(3)	139.6(3)	139.1(3)
C6‐C7	140.3(3)	141.2(3)	141.3(3)	140.4(3)
C7‐C8	138.2(3)	138.1(2)	138.8(3)	138.9(3)
C7‐C11	149.0(3)	148.4(3)	150.2(3)	149.0(3)
C8‐C8a	140.0(3)	139.8(2)	140.7(3)	139.9(4)
C8a‐C9	146.1(4)	146.4(2)	146.8(3)	146.9(4)
C9‐N9a	137.7(3)	137.9(2)	138.6(3)	138.4(4)
C9‐O10	122.7(3)	122.6(2)	122.7(3)	122.5(3)
C1‐N9a‐C9	123.0(2)	123.9(1)	123.1(2)	123.5(2)
C3‐C3a‐N4	126.0(2)	126.9(2)	125.0(2)	126.1(3)
N4‐C4a‐C5	118.9(2)	119.1(1)	119.8(2)	119.5(2)
C6‐C7‐C11‐C12	−37.0(3)	47.8(3)	−11.2(3)	−20.5(4)
C6‐C7‐C11‐C12′	142.9(2)		168.8(2)	157.3(2)
C6‐C7‐C11‐C17a		−134.6(2)		
C6‐C5‐C19‐C20			48.4(3)	
C6‐C5‐C19‐C20′			−130.4(2)	
C6‐C5‐C17‐C18				38.4(3)
C6‐C5‐C17‐C18′				−137.3(2)

**Scheme 4:**
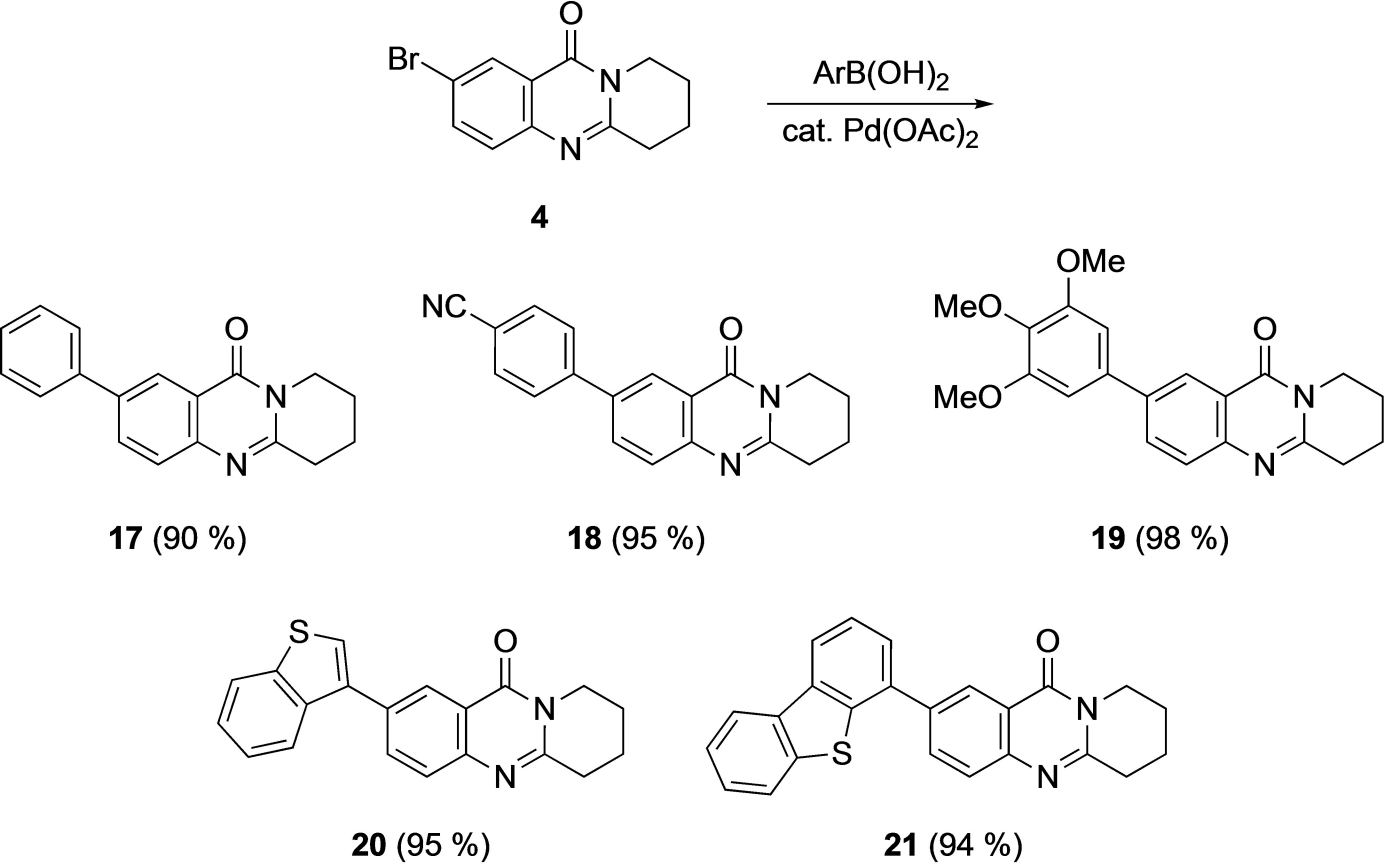
Mackinazolinone derivatives **17**–**21**.

Starting from bromomackinazolinone **4** with one methylene group more than 7‐bromo‐2,3‐dihydropyrrolo[2,1‐b]quinazoline‐9‐(1H)‐one (**3**) new arylated derivatives **17**–**21** were prepared by Suzuki‐Miyaura coupling reactions in 90 % and 95 % yield, respectively (Scheme 4). Remarkably, according to a SciFinder® search, there are so far only eight arylated mackinazolinione derivatives known including a patent in the context of electroluminescent properties.^[51]^

**Figure 5:**
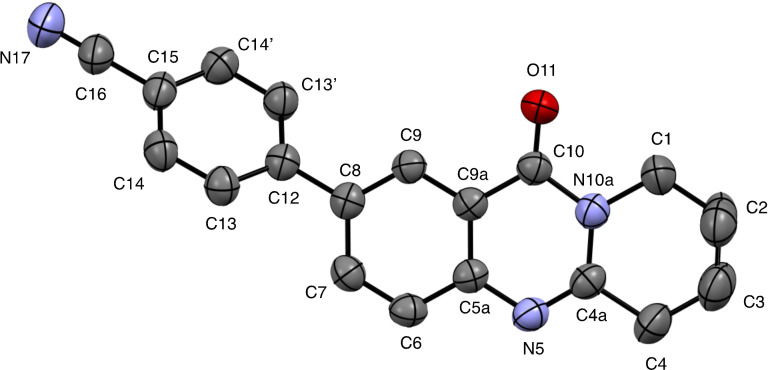
Structure of **18** in the crystal.^[49]^ Ellipsoids at 50 % probability level. Hydrogen atoms omitted for clarity. For selected bond lengths, bond angles and torsional angles see Table 4.

**Figure 6:**
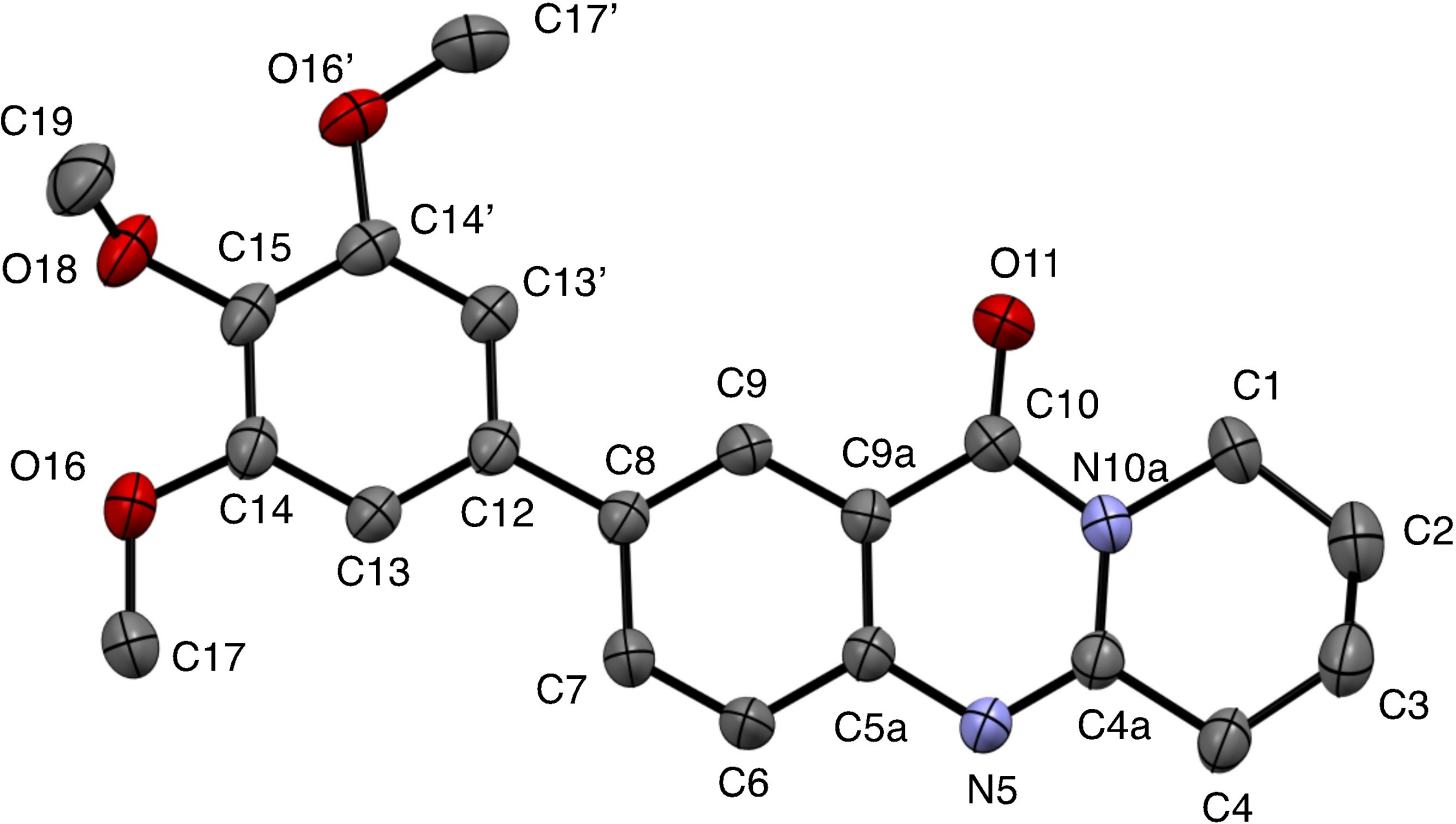
Structure of **19** in the crystal.^[49]^ Ellipsoids at 50 % probability level. Hydrogen atoms omitted for clarity. For selected bond lengths, bond angles and torsional angles see Table 4.

In addition to their spectroscopic characterization, **18** and **19** were subjected to crystal structure analyses (Figures 5 and 6). Selected geometrical data of **18**, and **19** are listed in Table 4.

**Table 4:** Selected bond lengths, bond angles, and torsion angles of mackinazolinone derivatives **18** and **19**. For atom numbering schemes see Figures 5–6.

Bond length [pm], bond angle [°] or torsion angle [°]	**18**	**19**
C1‐C2	150.8(7)	151.9(3)
C1‐N10a	148.3(2)	148.0(2)
C2‐C3	151.1(7)	147.3(4)
C3‐C4	150.1(5)	149.9(3)
C4‐C4a	151.1(3)	150.4(3)
C4a‐N5	130.0(2)	129.3(2)
C4a‐N10a	137.5(2)	137.4(2)
N5‐C5a	138.7(2)	138.2(2)
C5a‐C6	140.1(2)	139.7(3)
C5a‐C9a	140.1(2)	138.7(2)
C6‐C7	137.7(2)	136.5(3)
C7‐C8	140.7(2)	140.5(3)
C8‐C9	138.8(2)	138.4(2)
C8‐C12	148.4(2)	148.7(2)
C9‐C9a	140.0(2)	139.6(2)
C9a‐C10	145.5(2)	145.6(2)
C10‐N10a	140.5(2)	140.1(2)
C10‐O11	121.8(2)	122.0(2)
C1‐N10a‐C10	114.8(1)	114.5(2)
C4‐C4a‐N5	116.9(1)	117.2(2)
C7‐C8‐C12‐C13	40.5(2)	13.7(3)
C7‐C8‐C12‐C13′	−138.9(1)	−167.4(2)

In **18** the methylene groups of the molecule are disordered over two positions (C2, C3). Refinement of the structure yielded 0.628 (12) : 0.372 (12) occupancy ratio of the disordered atoms (i. e. two conformers). The disorder of the methylene groups was previously observed in the quinazoline derivative crystals of 2‐bromo‐6,8,9,11‐tetrahydro‐7*H*‐pyrido[2,1‐*b*]quinazolin‐11‐ol and 6,8,9,11‐tetrahydro‐7*H*‐pyrido[2,1‐*b*]quinazolin‐11‐ol.^[52]^ The piperidine ring with two *sp* ^*2*^‐hybridized atoms of C4a and N10a adopts half‐chair conformation.

**Figure 7:**
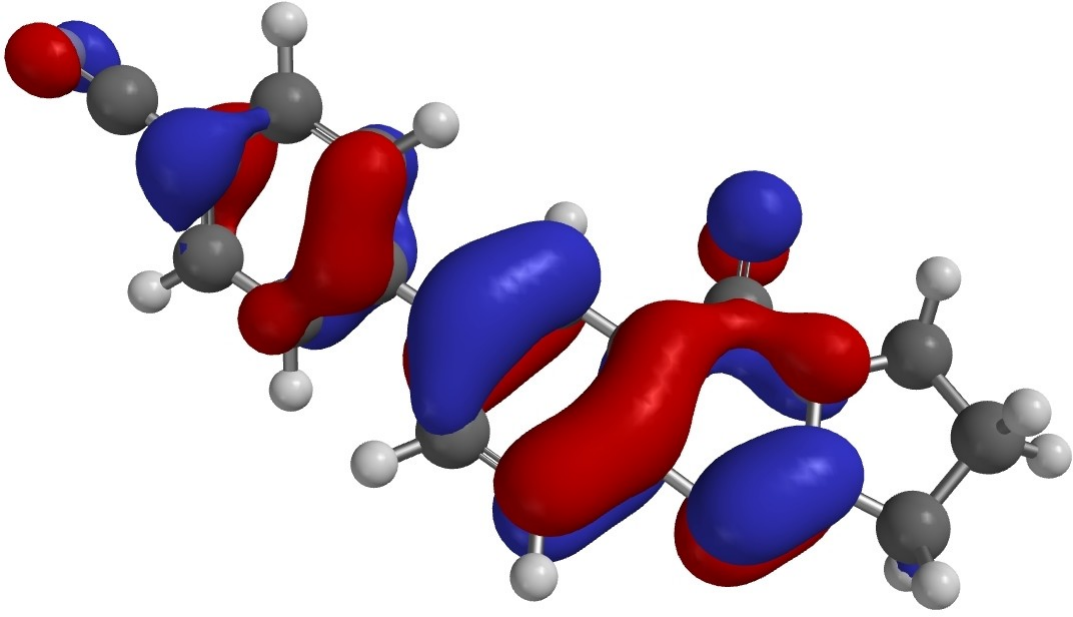
HOMO of **7** as calculated by DFT.^[53]^

**Figure 8:**
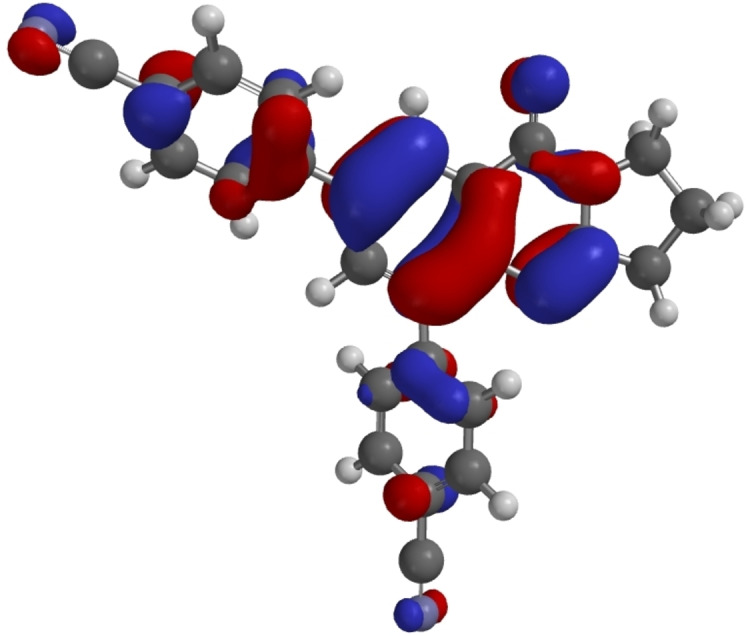
HOMO of **14** as calculated by DFT.^[53]^

Some DFT calculations of **1** and **6**–**11** have been performed.^[53]^ Calculated HOMO and LUMO energies are listed in Table 5, which also contains the energies of the HOMO‐LUMO gaps and the torsional angles between the aryl substituents and the quinazoline system based on the structure analyses and on the calculations. As a representative examples, the HOMOs of **7** and **14** are shown in Figures 7 and 8.

**Table 5:** Calculated HOMO and LUMO energies in eV and torsional angles.^[53]^ Groups **A**‐**C** refer to 7‐monosubstituted (**A**), 5,7‐disubstituted deoxyvasicinone (**B**), and 8‐monosubstituted mackinazolinone (**C**) derivatives.

Group	Compound	Formula	HOMO [eV]	LUMO [eV]	Δ [eV]	Torsion^[a]^
A	1	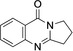	−8.33	0.48	8.81	–
	6	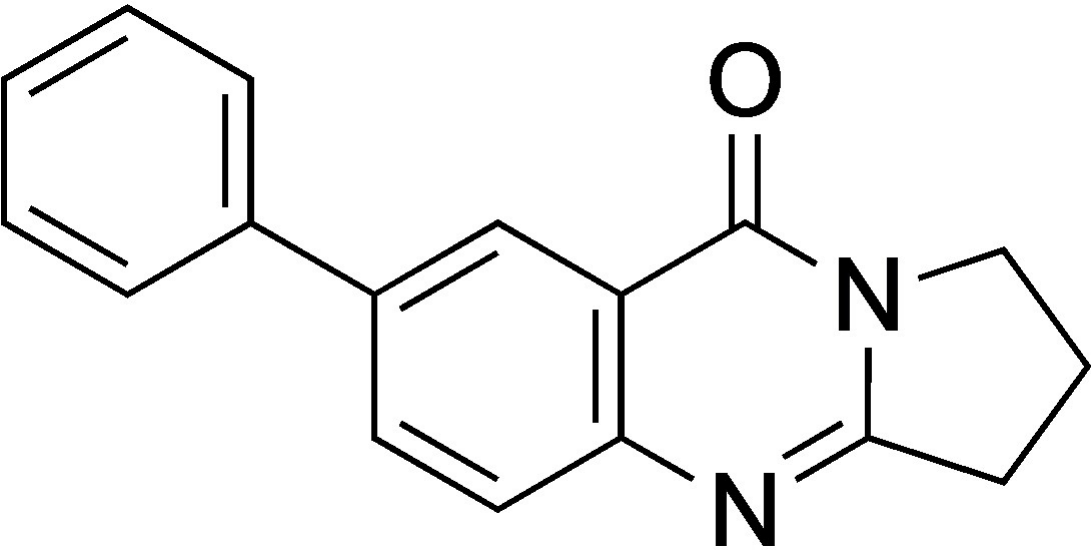	−7.98	0.38	8.36	C7 : 40.8°
	7	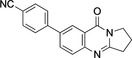	−8.34	−0.34	8.00	C7 : 39.9°
	8	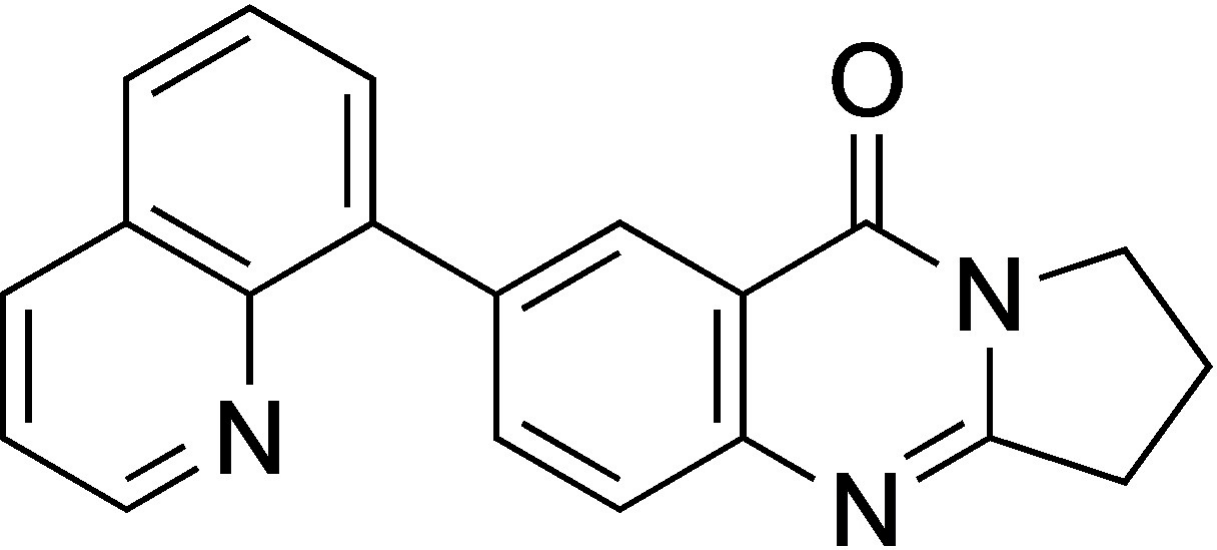	−7.84	−0.10	7.74	C7 : 51.0°
	9	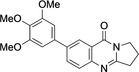	−7.86	0.40	8.26	C7 : *37.0°*/39.6°
	10	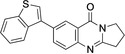	−7.77	0.39	8.16	C7 : *47.8°*/51.3°
	11	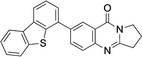	−7.81	0.28	8.09	C7 : 57.1°
B	12	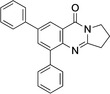	−7.87	0.28	8.15	C5 : 50.4° C7 : 40.3°
	13	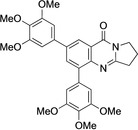	−7.67	0.29	7.96	C5 : *48.4°*/50.1° C7 : *11.2°*/40.0°
	14	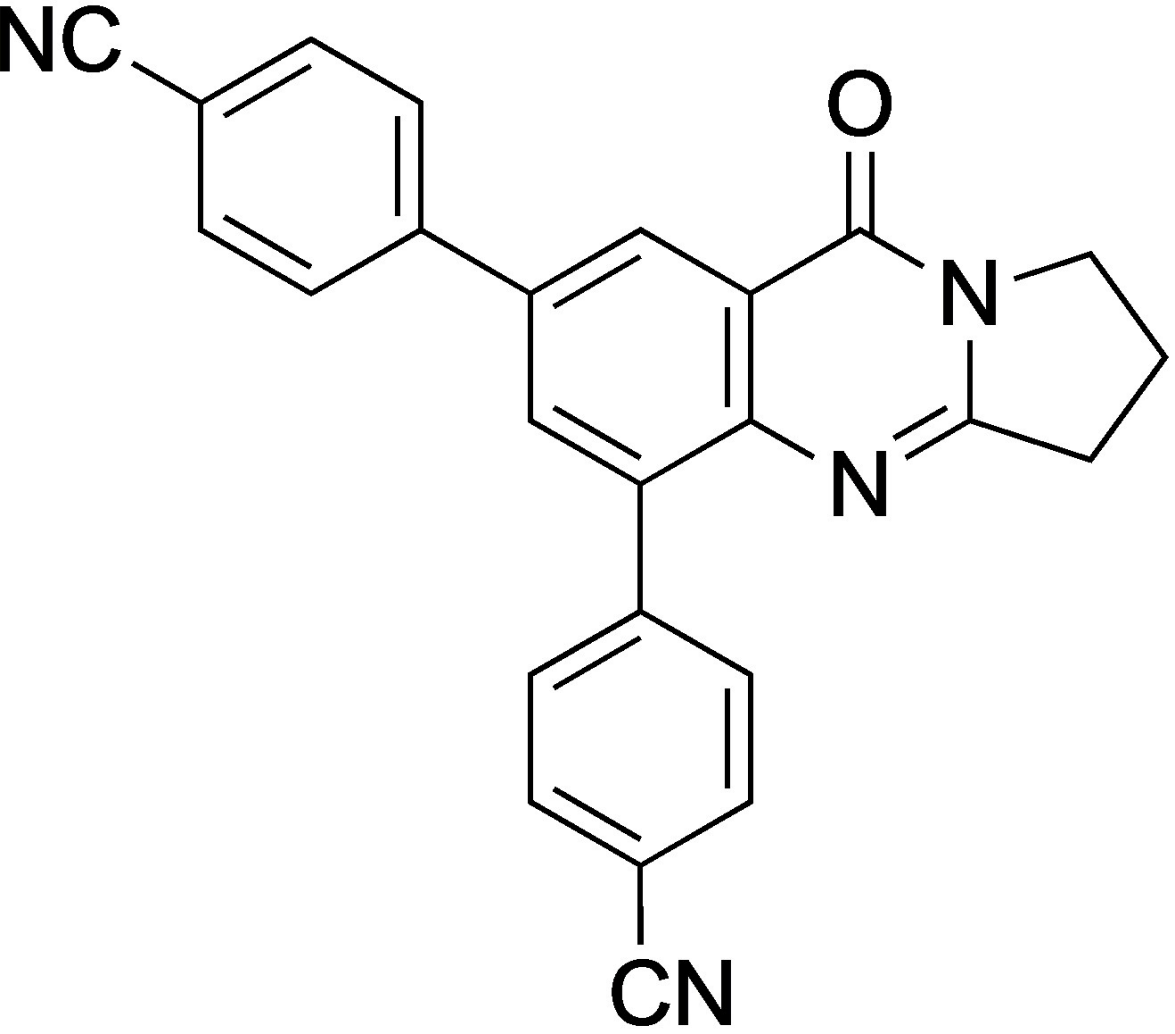	−8.19	−0.28	7.91	C5 : *38.4°*/47.6° C7 : *20.5°*/39.2°
	15^*b*^	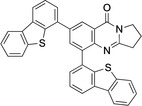	−7.43	0.56	7.99	C5 : 67.5° C7 : 51.5°
	16	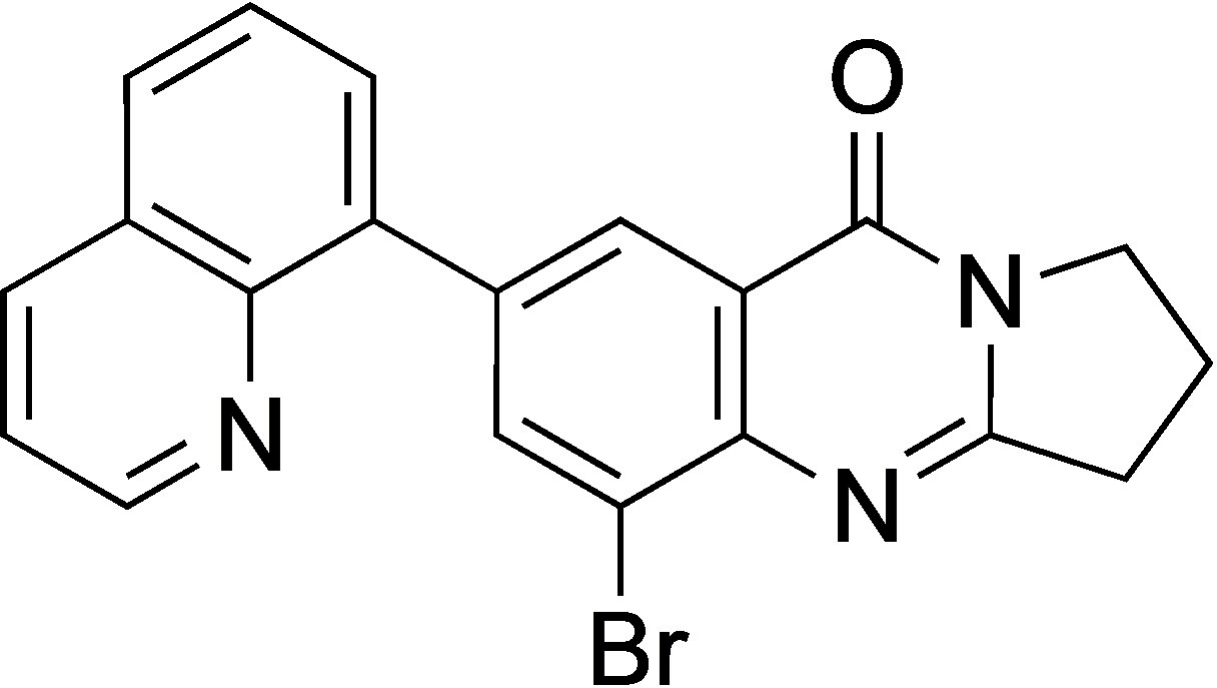	−7.94	−0.22	7.72	C7 : 49.3°
C	17	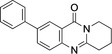	−7.94	0.39	8.33	C8 : 41.1°
	18	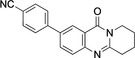	−8.29	−0.32	7.97	C8 : *40.5°*/40.3°
	19	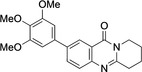	−7.73	0.42	8.15	C8 : *13.7°*/40.8°
	20	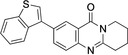	−7.71	0.38	8.09	C8 : 48.2°
	21	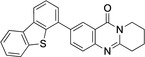	−7.78	0.28	8.06	C8 : 55.9°

[a] The numbers give the torsional angle between the connected π systems at the indicated carbon atom. Torsional angle signs omitted for clarity. *Italics*: by X‐ray structure analysis; non‐italics: by DFT calculation (ωB97X−D, 6–311+G**). [b] Calculation with 6–311+G** failed, data calculated with 6–31G*.

The data show that the calculated torsions of the substituents at C7 in **9** and **10** are reasonably in agreement with those of the structure analyses. For **13** and **14** this is the case only for the substituents at C5, while the calculated torsions at C7 significantly deviate from those measured. While there is an excellent agreement between the measured and the calculated torsion for **18**, this is not the case for the methoxy substituted derivative **19**. A possible reason for the observed deviations may be the fact that the calculations were done for the gas phase, while the measurements show the compounds in the crystalline state more prone to intermolecular interactions.

**Figure 9:**
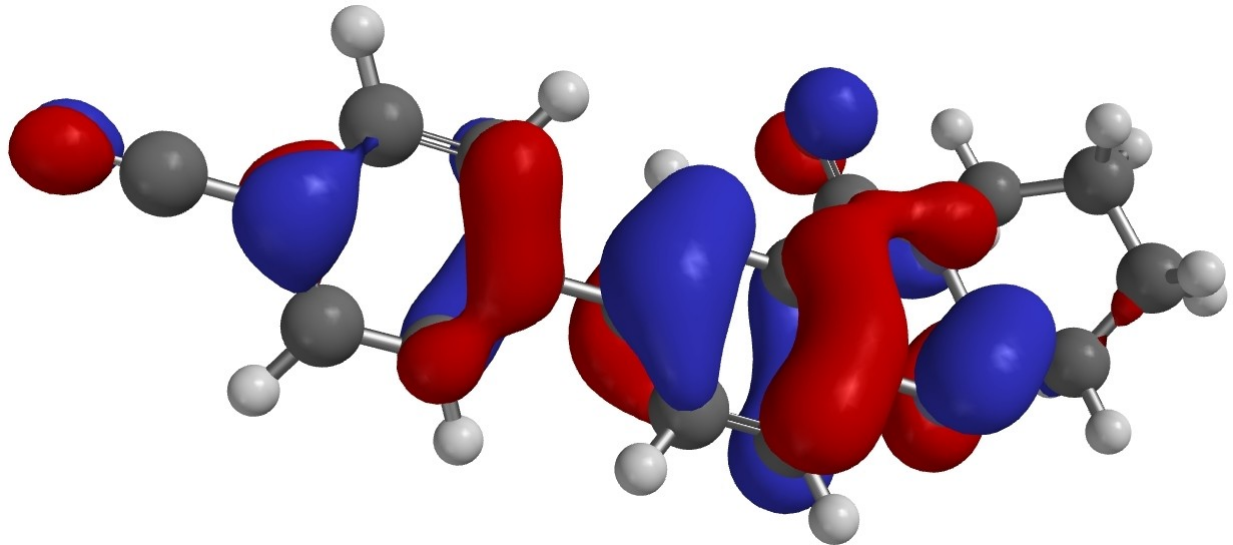
HOMO of **18** as calculated by DFT.^[53]^

**Figure 10:**
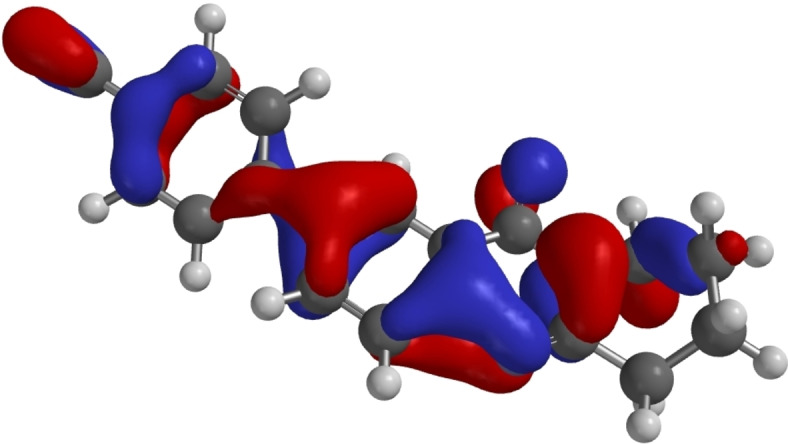
HOMO‐6 of **18** as calculated by DFT.^[53]^

As representative examples Figures 7, 8, 9 show the HOMOs of **7**, **14**, and **18**. These derivatives are those with the lowest HOMO in the respective group as a result of the electron withdrawing cyano substituents. All three HOMOs show a nodal plane between the substituent and the quinazoline π systems. For **18** HOMO‐6 is the highest π orbital with a significant overlap between the aryl substituent and the quinazoline π system as shown in Figure 10.

Within group **A** (7‐monosubstituted deoxyvasicinones) 4‐cyanophenyl derivative **7** with an electron withdrawing substituent has the lowest HOMO and also the lowest LUMO. Compound **10** with an electron rich benzo[*b*]thiophen‐3‐yl substituent has the highest HOMO and a similar LUMO as the electron rich **9**. The reliability of the calculations for **9** and **10** is high as indicated by the close matches for the torsional angles between the calculated and the measured values (*vide supra*). Within group **B** (5,7‐disubstituted deoxyvasicinones) the di(4‐cyanophenyl) substituted derivative **14** has the lowest HOMO and also the lowest LUMO with a HOMO‐LUMO gap of only 7.91 eV. The highly electron rich derivative **13** has the highest HOMO and also the highest LUMO in this group. Among the 2‐substituted mackinazolinone derivatives (group **C**) 4‐cyanophenyl derivative **18** has the lowest HOMO and also the lowest LUMO with excellent match of the calculated and measured torsional angle (*vide supra*). Electron rich derivatives **19** and **20** show the highest HOMOs. Over all, the (4‐phenyl) substituted derivatives **7**, **14**, and **18** consistently show the lowest HOMOs in their respective groups.

## Conclusions

In conclusion, we report the syntheses of a number of new deoxyvasicinone and mackinazolinone derivatives. The deoxyvasicinone derivatives bear aryl substituents at C7 and at C5, while mackinazolinone derivatives are substituted at C2. The systems with substituents at C5 or C2 are rare, so that the number of compounds of these types has significantly been increased. The compounds have been prepared in high yields by Suzuki‐Miyaura cross‐coupling reactions with palladium acetate as the catalyst and the respective boronic acids. Six of the compounds have been characterized by X‐ray crystal structure analyses showing very similar geometries for the tricyclic base systems of the derivatives. DFT calculations have been performed showing very good matches with the structural measurements of the torsional angles between the connected π systems for some cases. For all three groups of compounds the 4‐cyanophenyl substituted derivatives show the lowest HOMO energies.

## Experimental Section

**General**. Starting materials were either commercially acquired or were prepared according to published procedures.[^17^, ^19^, ^34^] ^1^H and ^13^C{^1^H} NMR spectra were obtained with Bruker AVS 400 (^1^H: 400 MHz, ^13^C: 100.6 MHz) and AVS 500 (^1^H: 500 MHz, ^13^C: 125.7 MHz) and with Varian Unity400 plus and Jeol JNM‐ECZ 600 R instruments. Chemical shifts *δ* refer to *δ*_*T*MS_=0 ppm (^1^H NMR) or to residual solvent signals (^13^C{^1^H} NMR). Primary, secondary, tertiary and quaternary carbon atom signals were identified as such by the APT or DEPT spectra. IR spectra were obtained with Perkin‐Elmer instruments FT‐IR 580 and 1170 using the ATR technique. Signal characteristics are abbreviated as s (strong), m (medium), w (weak), and br (broad). Mass spectra were obtained with a Micromass LCT instrument with lockspray source and direct injection and with a Q−TOF premier LC–MS/MS instrument with an Ionsabre‐APCI‐source (25 μA, 350 °C). In all cases acetonitrile was used as the solvent. Crystal structure analyses were obtained with CCD Xcalibur diffractometer (Oxford Diffraction) or Bruker SMART X2S instruments and were deposited with the CCDC. Analytical TLC was performed with Merck 60F‐254 silica gel thin layer plates. Column chromatography was performed with J. T. Baker silica gel (60 μm) as the stationary phase using the flash chromatography method.^[54]^ Melting points were measured with an instrument Electrothermal IA9000. All operations were performed in an argon atmosphere.

**General Procedure** (**GP**): 7‐Bromo‐2,3‐dihydropyrrolo[2,1‐*b*]quinazoline‐9‐(1*H*)*‐*one (**3**), 5,7‐dibromo‐2,3‐dihydro[2,1‐*b*]quinazoline‐9‐(1*H*)*‐*one (**5**) or 2‐bromo‐6,7,8,9‐tetrahydro‐11*H*‐pyrido[2,1‐*b*]quinazolin‐11‐one (**4**), arylboronic acid, sodium carbonate and palladium (II) acetate in acetone/water are stirred at 40–45 °C. After cooling the reaction mixture to 20–25 °C aqueous sodium hydroxide (1 M) is added, and the mixture is extracted with chloroform (2×15 mL) and then washed with water (2×5 mL). The organic layer is separated and dried over anhydrous sodium sulfate. After solvent removal at reduced pressure the product is purified by column chromatography or crystallized from benzene.

**7**‐**Bromo**‐**2,3**‐**dihydropyrrolo[2,1**‐***b*** **]quinazolin**‐**9(1*H*)**‐**one (7**‐**bromodeoxyvasicinone, 3**):[^1^, ^12^, ^13^, ^14^, ^15^, ^16^, ^17^, ^18^, ^19^, ^20^, ^21^, ^22^] At 0–2 °C phosphorus oxychloride (13 mL, 21.8 g, 142.0 mmol) is added dropwise with stirring over 1 h to anthranilic acid (4.32 g, 20.0 mmol) and γ‐butyrolactam (2.72 g, 32 mmol). After heating the reaction mixture at reflux for 2 h it is cooled to 25 °C, and ice (200 g) is added. Then aqueous ammonia (25 %) is added up to pH 10–11. The yellow precipitate is filtered off and dried. After recrystallization from methanol 7‐bromo‐2,3‐dihydropyrrolo[2,1‐*b*]quinazolin‐9(1*H*)‐one (**3**, 4.35 g, 16.4 mmol, 82 %) is obtained as yellow crystals (m. p. 159–160 °C).

^1^H NMR (400 MHz, CDCl_3_): *δ*=8.40 (1H, d, *J*=2.4 Hz, NCCHCHCC*H*), 7.80 (1 H, dd, *J*=2.4, 8.8 Hz, NCCHC*H*), 7.52 (1H, d, *J*=8.8 Hz, NCC*H*), 4.22 (2H, t, *J*=8.0 Hz, NCH_2_), 3.18 (2H, t, *J*=7.6 Hz, NCH_2_CH_2_C*H*_2_), 2.31 (2 H, m, NCH_2_C*H*_2_) ppm. ^13^C{^1^H} NMR (100.6 MHz, CDCl_3_): *δ*=159.9 (CO), 159.8 (OCC*C*H), 148.0 (OC*C*), 137.3 (N*C*CH), 129.0 (N*C*CH_2_), 128.7 (OCCCHC*CH*), 122.0 (NC*C*H), 119.7 (CBr), 46.7 (NCH_2_), 32.6 (NCH_2_CH_2_ *C*H_2_), 19.5 (NCH_2_ *C*H_2_) ppm.

**2**‐**Bromo‐6,7,8,9**‐**tetrahydro**‐**11*H***‐**pyrido[2,1**‐***b*** **]quinazolin**‐**11**‐**one (2**‐**bromomackinazolinone, 4)**:[^17^, ^23^] At 0–2 °C phosphorus oxychloride (26 mL, 43.55 g, 280.0 mmol) is added dropwise with stirring over 1 h to 5‐bromoanthranilic acid (8.0 g, 40.0 mmol) and *δ*‐valerolactam (6.34 g, 64.0 mmol). After heating the reaction mixture at reflux for 2 h it is cooled to 25 °C, and ice (200 g) is added. Then aqueous ammonia (25 %) is added up to pH 10–11. The yellow precipitate is filtered off and dried. After recrystallization from methanol 2‐bromo‐6,7,8,9‐tetrahydro‐11*H*‐pyrido[2,1‐*b*]quinazolin‐11‐one (**4**, 8.6 g, 33.2 mmol, 83 %) is obtained as yellow crystals (m. p. 163–164 °C).

^1^H NMR (600 MHz, CDCl_3_): *δ*=8.35 (1 H, d, *J*=2.3 Hz, OCCCH), 7.75 (1 H, dd, *J*=2.4, 8.7 Hz, NCCHC*H*), 7.45 (1 H, d, *J*=8.7 Hz, NCCH), 4.04 (2 H, t, *J*=6.2 Hz, NCH_2_), 2.98 (2 H, t, *J*=6.7 Hz, NCCH_2_), 2.02–1.98, 1.95–1.91 (2×2 H, 2 m, NCH_2_C*H*_2_, NCH_2_CH_2_C*H*_2_) ppm. ^13^C{^1^H} NMR (150 MHz, CDCl_3_): *δ*=161.1 (CO), 155.6 (OCC*C*H), 146.2 (OC*C*), 137.5 (N*C*CH), 129.3 (N*C*N), 128.4 (OCCCHC*C*H), 121.8 (NC*C*H), 119.6 (CBr), 42.7 (NCH_2_CH_2_ *C*H_2_), 32.0 (NCH_2_ *C*H_2_), 22.1 (NCH_2_CH_2_CH_2_ *C*H_2_), 19.3 (NCH_2_) ppm.

**5,7**‐**Dibromo**‐**2,3**‐**dihydropyrrolo[2,1**‐***b*** **]quinazolin**‐**9(1*H*)**‐**one (5,7**‐**dibromodeoxyvasicinone, 5**):[^21^, ^24^] At 0–2 °C phosphorus oxychloride (23.8 mL, 39.86 g, 260.0 mmol) is added dropwise with stirring over 1 h to 3,5‐dibromoanthranilic acid (10.0 g, 34.0 mmol) and γ‐butyrolactam (4.44 g, 52.0 mmol). After heating the reaction mixture at reflux for 2 h it is cooled to 25 °C, and ice (200 g) is added. Then aqueous ammonia (25 %) is added up to pH 10–11. The yellow precipitate is filtered off and dried. After recrystallization from methanol 5,7‐dibromo‐2,3‐dihydropyrrolo[2,1‐*b*]quinazolin‐9(1*H*)‐one (**5**, 9.8 g, 28.6 mmol, 84 %) is obtained as yellow crystals (m. p. 243–244 °C).

^1^H NMR (600 MHz, CDCl_3_): *δ*=8.35 (1 H, m, NCCCHCC*H*), 8.09 (1 H, m, NCCC*H*), 4.19 (2 H, t, *J*=7.1 Hz, NCH_2_), 3.24 (2 H, t, *J*=8.0 Hz, NCH_2_CH_2_C*H*_2_), 2.30 (2 H, m, NCH_2_C*H*_2_) ppm. ^13^C{^1^H} NMR (150 MHz, CDCl_3_): *δ*=161.2 (CO), 159.3 (OCC*C*H), 146.0 (OC*C*), 140.4 (N*C*CH), 128.8 (N*C*CH_2_), 123.0 (NC*C*Br), 122.8 (NC*C*H), 119.5 (OCCCH*C*Br), 47.0 (NCH_2_), 33.0 (NCH_2_CH_2_ *C*H_2_), 19.6 (NCH_2_ *C*H_2_) ppm.

**7**‐**Phenyl**‐**2,3**‐**dihydropyrrolo[2,1**‐***b*** **]quinazolin**‐**9(1*H*)**‐**one** (**6**): GP; 7‐bromo‐2,3‐dihydropyrrolo[2,1‐*b*]quinazoline‐9‐(1*H*)*‐*one (**3**, 132.5 mg, 0.5 mmol), phenylboronic acid (137 mg, 1.1 mmol), sodium carbonate (106.0 mg, 1.0 mmol) and palladium (II) acetate (9.43 mg, 0.042 mmol) in acetone / water (1 : 1, 6 mL), aqueous sodium hydroxide (1 M, 2 mL), 7‐phenyl‐2,3‐dihydropyrrolo[2,1‐*b*]quinazolin‐9(1*H*)‐one (**6**, 120 mg, 0.46 mmol, 92 %), colorless solid (m. p. 170–171 °C).

*R*_f_=0.21 (ethyl acetate). ^1^H NMR (400 MHz, CDCl_3_): *δ*=8.51 (1 H, d, *J*=2.0 Hz, NCCHCHCC*H*), 7.98 (1 H, dd, *J*=2.4, 8.0 Hz, NCCHC*H*), 7.70 (3 H, t, *J*=8.0 Hz, *m*‐CH_Ph_, NCC*H*), 7.47 (2 H, d, *J*=8.0 Hz, *o*‐CH_Ph_), 7.38 (1 H, t, *J*=8.0 Hz, *p*‐CH_Ph_), 4.23 (2 H, t, *J*=8.0 Hz, NCH_2_), 3.20 (2 H, t, *J*=8.0 Hz, NCH_2_CH_2_C*H*_2_), 2.30 (2 H, m, NCH_2_C*H*_2_) ppm. ^13^C{^1^H} NMR (100.6 MHz, CDCl_3_): *δ*=161.1 (CO), 159.6 (OCC*C*H), 148.3 (OC*C*), 139.8 (OCCCH*C*), 139.3 (N*C*CH), 133.2 (N*C*CH_2_), 129.1 (OCCCHC*CH*), 127.9 (OCCCHC*C*), 127.4 (NC*C*H), 127.3 (*o*‐CH), 124.4 (*m*‐CH), 120.9 (*p*‐CH), 46.7 (NCH_2_), 32.7 (NCH_2_CH_2_ *C*H_2_), 19.7 (NCH_2_ *C*H_2_) ppm. IR (ATR): ν=1661 (C=O), 1609 (C=N), 1474 (C−N) cm^−1^. EI−MS: *m/*z (%)=262 ([M]^+^, 100), 261 ([M−H]^+^, 37), 219 ([M−H−(CH_2_)_3_]^+^, 6.2). HRMS: Calcd. for C_17_H_15_N_2_O ([M+H]^+^) 263.1184; found 263.1184. Calcd. for C_17_H_14_N_2_O 262.1106; found: 262.1109.

**4**‐**(9**‐**Oxo**‐**1,2,3,9**‐**tetrahydropyrrolo[2,1**‐***b*** **]quinazolin**‐**7**‐**yl)benzonitrile** (**7**): GP; 7‐bromo‐2,3‐dihydropyrrolo[2,1‐*b*]quinazoline‐9‐(1*H*)*‐*one (**3**, 132.5 mg, 0.5 mmol), 4‐cyanophenylboronic acid (80.8 mg, 0.55 mmol), sodium carbonate (132.5 mg, 1.25 mmol), palladium (II) acetate (9.43 mg, 0.042 mmol), acetone/water (2 : 1, 6 mL), aqueous sodium hydroxide (1 M, 2 mL), purification by column chromatography (20×1 cm, SiO_2_, ethyl acetate), 4‐(9‐oxo‐1,2,3,9‐tetrahydropyrrolo[2,1‐*b*]quinazolin‐7‐yl)benzonitrile (**7**, 138 mg, 0.48 mmol, 96 %), colorless crystals, m. p. 240–241 °C.

*R*_f_=0.16 (ethyl acetate). ^1^H NMR (400 MHz, CDCl_3_): *δ*=8.50 (1 H, d, *J*=2.3 Hz, OCCCH), 7.96 (1 H, dd, *J*=2.3, 8.5 Hz, NCCHCH), 7.73–7.80 (5 H, m, NCCH, 4 C_Ph_H), 4.24 (2H, t, *J*=7.2 Hz, NCH_2_), 3.21 (2 H, t, *J*=8.0 Hz, NCH_2_CH_2_C*H*_2_), 2.32 (2H, m, NCH_2_C*H*_2_) ppm. ^13^C{^1^H} NMR (100.6 MHz, CDCl_3_): *δ*=160.9 (C≡N), 160.4 (CO), 149.4 (OCC*C*H), 144.2 (OC*C*), 137.0 (OCCCH*C*), 132.9 (OCCCHCCCH*C*H, OCCCHCCCHCH*C*), 128.0 (OCCCHC*C*CH), 127.9 (OCCCHCC*C*H), 125.0 (N*C*N), 121.1 (NCCH*C*H), 118.9 (NC*C*H), 111.5 (N*C*CH), 46.8 (NCH_2_), 32.8 (NCH_2_CH_2_ *C*H_2_), 19.7 (NCH_2_ *C*H_2_). IR (ATR): *ν*=2970 (CH_2_), 2221 (CN), 1670 (C=O), 1600 (C=N), 1508 (C=C), 1462 (C−N) cm^−1^. MS (TOF, ES+): *m/*z (%)=288 ([M+H]^+^, 100), 261 ([M−CN]^+^, 2), 183 ([M−HCN−C_6_H_5_]^+^, 1.8), 142 ([M−C_6_H_5_−CN−(CH_2_)_3_]^+^, 3.1). HRMS: Calcd. for C_18_H_14_N_3_O ([M+H]^+^) 288.1137; found 288.1371.

**7**‐**(Quinolin**‐**8**‐**yl)**‐**2,3**‐**dihydropyrrolo[2,1**‐***b*** **]quinazolin**‐**9(1H)**‐**one** (**8**): GP; 7‐bromo‐2,3‐dihydropyrrolo[2,1‐*b*]quinazoline‐9‐(1*H*)*‐*one (**3**, 132.5 mg, 0.5 mmol), 8‐quinolinylboronic acid (103.8 mg, 0.6 mmol), sodium carbonate (132.5 mg, 1.25 mmol), palladium (II) acetate (9.43 mg, 0.042 mmol), acetone/water (2 : 1, 6 mL), stirring at 40–45 °C for 5 h, aqueous sodium hydroxide (1 M, 2 mL), purification by column chromatography (20×1 cm, SiO_2_, ethyl acetate/methanol 25 : 1), 7‐(quinolin‐8‐yl)‐2,3‐dihydropyrrolo[2,1‐*b*]quinazolin‐9(1H)‐one (**8**, 130 mg, 0.42 mmol, 83 %), light purple crystals, m. p. 215–216 °C.

*R*_f_=0.21 (ethyl acetate/methanol 15 : 1). ^1^H NMR (400 MHz, CDCl_3_): *δ*=8.97 (1 H, dd, *J*=1.8, 4.2 Hz, NCCC*H*CHCHCCH), 8.58 (1 H, dd, *J*=0.5, 2.1 Hz, OCCCH), 8.22 (1 H, dd, *J*=1.8, 8.3 Hz, NCHC*H*), 8.17 (1 H, dd, *J*=2.1, 8.4 Hz, NCCHC*H*), 7.87 (1 H, dd, *J*=1.5, 8.2 Hz, NCCCHC*H*CH), 7.83 (1 H, dd, *J*=1.5, 7.2 Hz, NCHCHC*H*C), 7.76 (1 H, d, *J*=8.4 Hz, NCCH), 7.63 (1 H, dd, *J*=7.2, 8.1 Hz, NCCC*H*CH), 7.44 (1 H, dd, *J*=4.2, 8.3 Hz, NCH), 4.24 (2 H, t, *J*=7.2 Hz, NCH_2_), 3.22 (2H, t, *J*=7.9 Hz, NCH_2_CH_2_C*H*_2_), 2.30–2.37 (2 H, m, NCH_2_C*H*_2_) ppm. ^13^C{^1^H} NMR (100.6 MHz, CDCl_3_): *δ*=161.3 (CO), 159.5 (OCCCH*C*), 150.5 (OC*C*), 148.6 (OCC*C*H), 146.0 (NC*C*H), 139.6 (N*C*CH), 138.0 (NCN), 137.3 (NCCH*C*H), 136.5 (OCCCHC*C*), 130.7 (OCCCHCC*C*), 128.9 (OCCCHCCC*C*), 128.2 (OCCCHCCN*C*H), 128.0 (OCCCHCCNCHCH*C*H), 126.5 (OCCCHCCNCH*C*H), 126.4 (OCCCHCC*C*H), 121.3 (OCCCHCCCHCH*C*H), 120.5 (OCCCHCCCH*C*HCH), 46.7 (NCH_2_), 32.7 (NCH_2_CH_2_ *C*H_2_), 19.7 (NCH_2_ *C*H_2_CH_2_) ppm. IR (ATR): *ν*=1678 (C=O), 1610 (C=N), 1546 (C=C), 1483 (C−N) cm^−1^. EI−MS: *m/z* (%)=313 ([M]^+^, 22), 312 ([M−H]^+^, 100). HRMS: Calcd. for C_20_H_14_N_3_O 312.1137; found: 312.1137. ESI HRMS (LCT): Calcd. for C_20_H_15_N_3_ONa ([M+Na]^+^) 336.1113; found: 336.1107.

**7**‐**(3,4,5**‐**Trimethoxyphenyl)**‐**2,3**‐**dihydropyrrolo[2,1**‐***b*** **]quinazolin**‐**9(1*H*)**‐**one** (**9**): GP; 7‐bromo‐2,3‐dihydropyrrolo[2,1‐*b*]quinazoline‐9‐(1*H*)*‐*one (**3**, 132.5 mg, 0.5 mmol), 3,4,5‐trimethoxyphenylboronic acid (127.2 mg, 0.6 mmol), sodium carbonate (132.5 mg, 1.25 mmol), palladium (II) acetate (9.43 mg, 0.042 mmol), acetone/water (2 : 1, 6 mL), stirring at 40–45 °C for 5 h, aqueous sodium hydroxide (1 M, 2 mL), purification by column chromatography (20×1 cm, SiO_2_, ethyl acetate / petroleum ether 7 : 1), 7‐(3,4,5‐trimethoxyphenyl)‐2,3‐dihydropyrrolo[2,1‐*b*]quinazolin‐9(1H)‐one (**9**, 171.3 mg, 0.47 mmol, 97 %), yellow crystals, m. p. 169–170 °C.

*R*_f_=0.13 (ethyl acetate/petroleum ether 7 : 1). ^1^H NMR (400 MHz, CDCl_3_): *δ*=8.47 (1 H, d, *J*=1.9 Hz, OCCCH), 7.96 (1 H, dd, *J*=2.2, 8.5 Hz, NCCHC*H*), 7.72 (1 H, d, *J*=8.5 Hz, NCCH), 6.88 (2 H, s, *o*‐CH), 4.25 (2 H, t, *J*=7.2 Hz, NCH_2_), 3.97 (6H, s, *m*‐COCH_3_), 3.92 (3H, s, *p*‐COCH_3_), 3.22 (2H, t, *J*=7.8 Hz, NCH_2_CH_2_C*H*_2_), 2.29–2.37 (2H, m, NCH_2_C*H*_2_) ppm. ^13^C{^1^H} NMR (100.6 MHz, CDCl_3_): *δ*=161.1 (CO), 159.4 (OCCCH*C*), 153.6 (OC*C*), 148.3 (NCCH*C*H), 139.2 (NC*C*H), 135.6 (N*C*CH), 133.1 (NCN), 127.3 (*o*‐CH), 124.1 (*m*‐*C*OCH_3_), 120.7 (*p*‐*C*OCH_3_), 104.4 (OCCCHC*C*), 60.9 (*p*‐CO*C*H_3_), 58.3 (*m*‐CO*C*H_3_), 46.6 (NCH_2_), 32.5 (NCH_2_CH_2_ *C*H_2_), 19.5 (NCH_2_ *C*H_2_CH_2_) ppm. IR (ATR): *ν*=1672 (C=O), 1620 (C=N), 1481 (C−N) cm^−1^. EI‐MS: *m/z* (%)=352 ([M]^+^, 96), 337 ([M−CH_3_]^+^, 100), 309 (8.4), 279 (5.3), 223 (9.5), 176 (4.2), 139 (3.1), 111 (6.3). HRMS: Calcd. for C_20_H_20_N_2_O_4_ 352.1423; found 352.1422.

Crystal structure analysis:^[49]^ CCDC 2344447. Single crystals suitable for X‐ray crystallographic analysis were obtained by crystallization from ethyl acetate at −15 °C. C_20_H_20_N_2_O_4_, yellow prism, *M*_r_=352.38 g . mol^−1^, crystal system monoclinic, space group P 1 21/c 1, *a*=13.9978(18), *b*=7.4552(9), *c*=17.018(2) Å, *α*=90°, *β*=103.547(4)°, *γ*=90°. *V*=1726.5(4) Å^3^, *Z*=4, *d*_calc_=1.356 g cm^−3^, *μ*=0.094 mm^−1^, crystal size 0.62×0.26×0.20 mm^3^, *F*(000)=744, Bruker SMART X2S diffractometer, graphite crystal monochromator, *T*=200 K, Mo−K_*α*_ radiation (*λ*=0.71073 Å), 2.46≤*θ*≤27.59°, index ranges −18≤*h*≤18, −9≤*k*≤9, −20≤*l*≤22, reflections collected/unique 3980/2568, numerical absorption correction, structure solution and refinement with SHELXL‐2018/3,^[55]^ parameters/restrains 238/0, *R*_1_=0.0647 [*l*>2 s(*l*)], *wR*_2_=0.1638 (all data), *S*=1.022, final maximum/minimum difference electron density 0.268/–0.248 eÅ^−3^.

**7**‐**(Benzo[*b*]thiophen**‐**3**‐**yl)**‐**2,3**‐**dihydropyrrolo[2,1**‐***b*** **]quinazolin**‐**9**(**1** **H)**‐**one** (**10**): GP; 7‐bromo‐2,3‐dihydropyrrolo[2,1‐*b*]quinazoline‐9‐(1*H*)*‐*one (**3**, 132.5 mg, 0.5 mmol), benzothiophene‐3‐boronic acid (106.8 mg, 0.6 mmol), sodium carbonate (132.5 mg, 1.25 mmol), palladium (II) acetate (9.43 mg, 0.042 mmol) in acetone/water (2 : 1, 6 mL), stirring at 40–45 °C for 3 h, then for 8 h a 25 °C, aqueous sodium hydroxide (1 M, 2 mL), purification by column chromatography (20×1 cm, SiO_2_, ethyl acetate / petroleum ether 8 : 1), 7‐(benzo [*b*] thiophen‐3‐yl)‐2,3‐dihydropyrrolo[2,1‐*b*]quinazolin‐9(1H)‐one (**10**, 142 mg, 0.45 mmol, 89 %), colorless crystals, m. p. 340 °C.

*R*_f_=0.18 (ethyl acetate/petroleum ether 8 : 1). ^1^H NMR (400 MHz, CDCl_3_): *δ*=8.53 (1 H, d, *J*=2.0 Hz, OCCCH), 7.95–8.00 (3 H, m, NCCHC*H*, H‐6, SCCH, SCCHCHC*H*), 7.78 (1 H, d, *J*=8.4 Hz, NCCH), 7.54 (1 H, s, SCH), 7.42–7.46 (2H, m, SCCHC*H*, SCCHCHCHC*H*), 4.27 (2 H, t, *J*=8.1 Hz, NCH_2_), 3.24 (2 H, t, *J*=7.8 Hz, NCH_2_CH_2_C*H*_2_), 2.30–2.38 (2 H, m, NCH_2_C*H*_2_) ppm.^13^C{^1^H} NMR (100.6 MHz, CDCl_3_): *δ*=161.0 (CO), 159.6 (NCCHCH*C*), 148.5 (NCCHCHC*C*), 140.7 (OCC*C*H), 137.6 (SCH), 136.7 (OC*C*), 134.6 (N*C*C), 134.1 (NC*C*H), 127.3 (NCCH*C*H), 125.9 (N*C*CH_2_), 124.6 (S*C*CH, SC*C*CH), 124.4 (SCCHCHCH*C*H), 123.0 (SC*C*H), 122.7 (SCCH*C*H), 120.8 (SCCHCH*C*H), 46.6 (NCH_2_), 32.6 (NCH_2_CH_2_ *C*H_2_), 19.6 (NCH_2_ *C*H_2_) ppm. IR (ATR): *ν*=1675 (C=O), 1618 (C=N), 1474 (C−N) cm^−1^. HRMS: Calcd. for C_19_H_14_N_2_NaO ([M+Na]^+^) 341.0725; found: 341.0725.

Crystal structure analysis:^[49]^ CCDC 2344448. Single crystals suitable for X‐ray crystallographic analysis were obtained by crystallization from toluene at 5 °C. C_19_H_14_N_2_OS, colorless needle, *M*_r_=318.38 g . mol^−1^, crystal system orthorhombic, space group P b c a, *a*=17.041(3), *b*=7.0846(10), *c*=25.021(4) Å, *α*=90°, *β*=90°, *γ*=90°, *V*=3020.8(8) Å^3^, *Z*=8, *d*_calc_=1.400 g cm^−3^, *μ*=0.220 mm^−1^, crystal size 0.97×0.28×0.25 mm^3^, *F*(000)=1328, Bruker SMART X2S diffractometer, graphite crystal monochromator, *T*=300(2) K, Mo−K_*α*_ radiation (λ=0.71073 Å), 2.39≤*θ*≤28.66°, index ranges −22≤*h*≤22, −9≤*k*≤9, −33≤*l*≤33, reflections collected/unique 3868/2944, multi‐scan absorption correction, structure solution and refinement with SHELXL‐2018/3,^[55]^ parameters/restrains 208/0, *R*_1_=0.0532 [*l*>2 s(*l*)], *wR*_2_=0.1457 (all data), *S*=1.020, final maximum/minimum difference electron density 0.351/–0.485 eÅ^−3^.

**7**‐**(Dibenzo[*b,d*] thiophen**‐**4**‐**yl)**‐**2,3**‐**dihydropyrrolo[2,1**‐***b*** **]quinazolin**‐**9**(**1*H***)‐**one** (**11**): GP; 7‐bromo‐2,3‐dihydropyrrolo[2,1‐*b*]quinazoline‐9‐(1*H*)*‐*one (**3**, 132.5 mg, 0.5 mmol), dibenzothiophene‐4‐boronic acid (137 mg, 0.60 mmol), sodium carbonate (132.5 mg, 1.25 mmol), palladium (II) acetate (9.43 mg, 0.042 mmol) in acetone/water (2 : 1, 6 mL), stirring at 40–45 °C for 7 h, aqueous sodium hydroxide (1 M, 2 mL), purification by column chromatography (20×1 cm, SiO_2_, ethyl acetate/petroleum ether 4 : 1), 7‐(dibenzo[*b,d*]thiophen‐4‐yl)‐2,3‐dihydropyrrolo[2,1‐*b*]quinazolin‐9(1*H*)‐one (**11**, 168 mg, 0.46 mmol, 91 %), yellow crystals, m. p. 180–182 °C.

*R*_f_=0.2 (ethyl acetate/petroleum ether 4 : 1). ^1^H NMR (400 MHz, CDCl_3_): *δ*=8.67 (1 H, d, *J*=2.1 Hz, OCCCH), 8.20–8.23 (2 H, m, C_arom_H), 8.14 (1 H, dd, *J*=2.2, 8.4 Hz, NCCHC*H*), 7.84–7.87 (1 H, m, C_arom_H), 7.80 (1 H, d, *J*=8.4, NCCH), 7.57–7.59 (2H, m, C_arom_H), 7.46–7.49 (2H, m, C_arom_H), 4.25 (2H, t, *J*=7.2 Hz, NCH_2_), 3.23 (2H, t, *J*=7.8 Hz, NCH_2_CH_2_C*H*_2_), 2.34 (2 H, m, NCH_2_C*H*_2_) ppm. ^13^C{^1^H} NMR (100.6 MHz, CDCl_3_): *δ*=161.1 (CO), 160.0 (NCCHCH*C*), 148.9 NCCHCHC*C*), 139.6 (OCC*C*H), 138.8 (NCCHCHC*C*S), 138.7 (NCCHCHCCS*C*), 136.6 (N*C*CH), 135.8 (NC*C*H), 135.8 (NCCH*C*H), 134.3 (NCN), 127.5 (S*C*CH), 127.3 (SC*C*H), 127.1 (SC*C*CH), 126.2 (SCC*C*CH), 125.4 (NCCHCHCCCH*C*H), 124.6 (SCCH*C*H), 122.8 (SCC*C*H), 121.9 (SCCHCH*C*H), 121.0 (NCCHCHC*C*), 120.9 NCCHCHCC*C*H), 46.7 (NCH_2_), 32.8 (NCH_2_CH_2_ *C*H_2_), 19.7 (NCH_2_ *C*H_2_) ppm. IR (ATR): ν=2968 (CH_2_), 1661 (C=O), 1612 (C=N), 1496 (C=C), 1423 (C−N) cm^−1^. EI−MS: *m/z* (%)=368 ([M]^+^, 4), 264 (86), 150 (14), 131 (100). HRMS: Calcd. for C_23_H_16_N_2_OS 368.0983; found 368.0982.

**5,7**‐**Diphenyl**‐**2,3**‐**dihydropyrrolo[2,1**‐***b*** **]quinazolin**‐**9**(**1H**)‐**one** (**12**): GP; 5,7‐dibromo‐2,3‐dihydropyrrolo[2,1‐*b*]quinazoline‐9‐(1*H*)*‐*one (**5**, 172 mg, 0.5 mmol), phenylboronic acid (146.3 mg, 1.2 mmol), sodium carbonate (265 mg, 2.5 mmol), palladium (II) acetate (18.86 mg, 0.084 mmol) in acetone/water (1 : 1, 12 mL), stirring at 40–45 °C for 4 h, aqueous sodium hydroxide (1 M, 2 mL), purification by column chromatography (20×1 cm, SiO_2_, ethyl acetate/petroleum ether 1 : 1), 5,7‐diphenyl‐2,3‐dihydropyrrolo[2,1‐*b*]quinazolin‐9(1H)‐one (**12**, 142 mg, 0.42 mmol, 84 %), colorless crystals, m. p. 207–208 °C.

*R*_f_=0.27 (ethyl acetate/petroleum ether 1 : 1). ^1^H NMR (400 MHz, CDCl_3_): *δ*=8.56 (1 H, d, *J*=2.4 Hz, OCCC*H*CCH), 8.02 (1 H, d, *J*=2.4 Hz, OCCCHCC*H*), 7.67–7.75 (4 H, dt, *J*=1.2, 8.8 Hz, 2×2 *o*‐CH), 7.50 (4 H, t, *J*=7.2 Hz, 2×2 *m*‐CH), 7.46–7.48 (2H, td, *J*=1.2, 7.6 Hz, 2 *p‐*CH), 4.24 (2 H, t, *J*=7.2 Hz, NCH_2_), 3.14 (2H, t, *J*=8.0 Hz, NCH_2_CH_2_C*H*_2_), 2.23–2.31 (2H, m, NCH_2_ C*H*_2_) ppm. ^13^C{^1^H} NMR (100.6 MHz, CDCl_3_): *δ*=161.5 (CO), 158.7 (OCC*C*H), 146.0 (OC*C*), 139.8 (OCC*C*), 139.5 (OCCCH*C*), 138.9 (OCCCHCCH*C*), 138.7 (NCN), 134.3 (OCCCHC*C*H), 130.7 (*ipso*‐C), 129.1 (*ipso*‐C), 128.1 (*o*‐CH), 127.9 (*o*‐CH), 127.6 (*m*‐CH), 127.3 (*m*‐CH), 123.8 (*p*‐CH), 121.7 (*p*‐CH), 46.6 (NCH_2_), 32.9 (NCH_2_CH_2_ *C*H_2_), 19.8 (NCH_2_ *C*H_2_) ppm. IR (ATR): ν=1670 (C=O), 1604 (C=N), 1494 (C=C), 1477 (C−N) cm^−1^. EI‐MS: *m/z* (%)=338 ([M]^+^, 37), 337 ([M−H]^+^, 100). HRMS: Calcd. for C_23_H_17_N_2_O 337.1341; found 337.1340. ESI HRMS (LCT): Calcd. for C_23_H_19_N_2_O ([M+H]^+^) 339.1497; found: 339.1497.

**5,7**‐**Bis (3,4,5**‐**trimethoxyphenyl)**‐**2,3**‐**dihydropyrrolo[2,1**‐***b*** **]quinazolin**‐**9**(**1H**)‐**one** (**13**): GP; 5,7‐dibromo‐2,3‐dihydropyrrolo[2,1‐*b*]quinazoline‐9‐(1*H*)*‐*one (**5**, 103 mg, 0.3 mmol), (3,4,5‐trimethoxyphenyl) boronic acid (148.3 mg, 0.70 mmol), sodium carbonate (79.5 mg, 0.75 mmol), palladium (II) acetate (7.2 mg, 0.03 mmol) in acetone/water (1 : 1, 10 mL), stirring at 40–45 °C for 8 h, aqueous sodium hydroxide (1 M, 2 mL), purification by column chromatography (20×1 cm, SiO_2_, ethyl acetate/petroleum ether 6 : 1), 5,7‐bis (3,4,5‐trimethoxyphenyl)‐2,3‐dihydropyrrolo[2,1‐*b*]quinazolin‐9(1H)‐one (**13**, 151 mg, 0.29 mmol, 97 %), colorless crystals, m. p. 257–258 °C.

*R*_f_=0.20 (ethyl acetate/petroleum ether 6 : 1). ^1^H NMR (400 MHz, CDCl_3_): *δ*=8.48 (1 H, d, *J*=2.3 Hz, OCCH), 7.95 (1 H, d, *J*=2.3 Hz, OCCCHCC*H*), 6.90 (4 H, 2 s, 4 *o*‐CH), 4.25 (2 H, t, *J*=7.1 Hz, NCH_2_), 3.95 (6 H, s, *m*‐OCH_3_), 3.93 (3 H, s, *p*‐OCH_3_), 3.91 (6 H, s, *m*‐OCH_3_), 3.91 (3 H, s, *p*‐OCH_3_), 3.17 (2 H, t, *J*=7.8 Hz, NCH_2_CH_2_C*H*_2_), 2.29 (2 H, m, NCH_2_C*H*_2_) ppm. ^13^C{^1^H} NMR (100.6 MHz, CDCl_3_): *δ*=161.4 (CO), 158.9 (OC*C*), 153.8 (*ipso*‐C), 153.0 (*ipso*‐C), 145.8 (N*C*CCH), 139.4 (OCCCH*C*), 139.0 (OCC*C*H), 138.3 (NC*C*CH), 137.8 (NCC*C*H), 135.7 (*p*‐C), 134.2 (*o*‐CH), 134.0 (*o*‐CH), 123.7 (2 C, *m*‐C), 121.6 (2 C, *m*‐C), 108.1 (*p*‐C), 104.7 (NCN), 61.1 (6 C, *m*‐O*C*H_3_), 61.0 (3 C, *p*‐O*C*H_3_), 56.5 (6 C, *m*‐O*C*H_3_), 56.4 (3 C, *p*‐O*C*H_3_), 46.7 (NCH_2_), 32.9 (NCH_2_CH_2_ *C*H_2_), 19.8 (NCH_2_ *C*H_2_CH_2_) ppm. IR (ATR): *ν* **=**2987, 2939 (CH_2_), 1670 (C=O), 1629 (C=N), 1508 (C=C), 1455 (C−N). EI‐MS: *m/z* (%)=518 ([M]^+^, 100), 519 ([M+H]^+^, 33), 332 ([M−6OMe]^+^, 51), 259 ([M−C_6_H(OMe)_3_]^+^, 20), 187 (23), 144 (quinazoline, 28). HRMS calcd. for C_29_H_30_N_2_O_7_ 518.2053; found: 518.2066.

Crystal structure analysis:^[49]^ CCDC 2344450. Single crystals suitable for X‐ray crystallographic analysis were obtained by crystallization from ethyl acetate at 5 °C. C_29_H_30_N_2_O_7_, colorless plate, M_r_=518.55 g . mol^−1^, crystal system monoclinic, space group P 1 21/n 1, *a*=7.821(3), *b*=12.927 (5), *c*=25.543(9) Å, *α*=90°, *β*=94.384 (13)°, *γ*=90°. V=2574.9(15) Å^3^, Z=4, d_calc_=1.338 g cm^−3^, μ=0.096 mm^−1^, crystal size 0.78×0.35×0.19 mm^3^, F(000)=1096.0, Bruker SMART X2S diffractometer, graphite crystal monochromator, T=300(2) K, Mo−K_*α*_ radiation (λ=0.71073 Å), 2.67≤θ≤27.56°, index ranges −10≤h≤9, −16≤k≤16, −33≤l≤31, reflections collected/unique 5760/3644, multi‐scan absorption correction, structure solution and refinement with SHELXL‐2018/3,^[55]^ parameters/restrains 349/0, R_1_=0.0629 [l>2 s(l)], wR_2_=0.1699 (all data), S=1.023, final maximum/minimum difference electron density 0.270/−0.262 eÅ^−3^.

**4,4′**‐**(9**‐**oxo**‐**1,2,3,9**‐**tetrahydropyrrolo[2,1**‐**b]quinazoline**‐**5,7**‐**diyl)dibenzonitrile** (**14**): GP; 5,7‐dibromo‐2,3‐dihydropyrrolo[2,1‐*b*]quinazoline‐9‐(1*H*)*‐*one (**5**, 103 mg, 0.3 mmol), 4‐cyanophenylboronic acid (103 mg, 0.70 mmol), sodium carbonate (79.5 mg, 0.75 mmol), palladium (II) acetate (7.2 mg, 0.03 mmol) in acetone/water (1 : 1, 10 mL), stirring at 40–45 °C for 8 h, aqueous sodium hydroxide (1 M, 2 mL), purification by column chromatography (20×1 cm, SiO_2_, ethyl acetate/petroleum ether 4 : 1), 4,4′‐(9‐oxo‐1,2,3,9‐tetrahydropyrrolo[2,1‐b]quinazoline‐5,7‐diyl) dibenzonitrile (**14**, 114.5 mg, 0.29 mmol, 98 %), colorless crystals, m. p. 261–262 °C.

*R*_f_=0.53 (ethyl acetate/petroleum ether 4 : 1). ^1^H NMR (400 MHz, CDCl_3_): *δ*=8.60 (1 H, d, *J*=2.3 Hz, OCCCH), 7.95 (1 H, d, *J*=2.3 Hz, OCCCHCC*H*), 7.81–7.83 (2 H, m, 2 *o*‐CH), 7.77 (6 H, m, 2 *o*‐CH, 4 *m*‐CH), 4.25 (2 H, t, *J*=7.2 Hz, NCH_2_), 3.15 (2 H, t, *J*=7.8, NCH_2_CH_2_C*H*_2_), 2.31 (2H, m, NCH_2_CH_2_) ppm. ^13^C{^1^H} NMR (100 MHz, CDCl_3_): *δ*=160.9 (CO), 160.3 (OC*C*), 146.7 (OCCCH*C*), 143.7 (NC*C*CCH), 143.1 (N*C*CCH), 138.0 (COC*C*H), 136.7 (NCN), 133.7 (OCCCHC*C*H), 133.0 (N*C*CCH), 131.9 (N*C*CCH), 131.3 (2 C, 2 *ipso*‐C), 127.9 (2 C, 2 NC*C*), 125.5 (NCC*C*HCHC), 122.0 (NCC*C*HCHC), 119.1 (NCC*C*H), 118.8 (NCC*C*H), 111.8 (NCCCH*C*H), 111.6 (NCCCH*C*H), 46.8 (NCH_2_), 32.9 (NCH_2_CH_2_ *C*H_2_), 19.7 (NCH_2_ *C*H_2_ *C*H_2_) ppm. IR (ATR): *ν*=2954 (CH_2_), 2226 (CN), 1676 (C=O), 1602 (C=N), 1504 (C=C), 1462 (C−N) cm^−1^. EI‐MS: *m/z* (%)=388 ([M]^+^, 3.3), 366 (89), 362 ([M−CN]^+^, 24), 351 (100), 286 ([M−C_6_H_4_CN]^+^, 3.2), 260 ([M−C_6_H_4_−2CN]^+^, 3.5), 184 ([M−2 C_6_H_4_CN]^+^, 5.4). HRMS calcd. for C_25_H_16_N_4_O 388.1324; found 388.1330.

Crystal structure analysis:^[49]^ CCDC 2345691. Single crystals suitable for X‐ray crystallographic analysis were obtained by crystallization from ethyl acetate at 25 °C. C_25_H_16_N_4_O, colorless plates, M_r_=388.42 g . mol^−1^, crystal system monoclinic, space group P 2_1_/c, a=10.9030(5), b=7.4973(4), c=46.124(3) Å, *α*=90°, *β*=95.000(5)°, *γ*=90°, V=3755.5(3) Å^3^, Z=8, Z′=2, d_calc_=1.374 g cm^−3^, *μ*=0.693 mm^−1^, crystal size 0.24×0.23×0.06 mm^3^, F(000)=1616, CryoLoop (20 micron, 0.2–0.3 mm, Hampton Research) XtaLAB AFC12 (RINC): Kappa single diffractometer, mirror monochromator, T=100.00(10) K, Cu−K_*α*_ radiation (*λ*=1.54184 Å), 1.923°≤θ≤82.122°, index ranges −13≤h≤13, −9≤k≤9, −56≤l≤58, reflections llllcollected/unique 37418/7983, multi‐scan absorption correction, structure solution with ShelXT 2018/2^[55]^ with Intrinsic Phasing solution method and by using Olex2^[56]^ as the graphical interface, refinement with ShelXL 2019/3,^[55]^ parameters/restraints 542/0, R_1_=0.0673 [l>2 s(l)], wR_2_=0.2004 (all data), S=1.137, final maximum/minimum difference electron density 0.415/−0.374 eÅ^−3^.

**5,7**‐**Bis(dibenzo[b,d]thiophen**‐**4**‐**yl)**‐**2,3**‐**dihydropyrrolo[2,1**‐**b]quinazolin**‐**9**(**1H**)‐**one** (**15**): GP; 5,7‐dibromo‐2,3‐dihydropyrrolo[2,1‐*b*]quinazoline‐9‐(1*H*)*‐*one (**5**, 103 mg, 0.3 mmol), dibenzothiophene‐4‐boronic acid (160 mg, 0.70 mmol), sodium carbonate (79.5 mg, 0.75 mmol), palladium (II) acetate (7.2 mg, 0.03 mmol) in acetone/water (1 : 1, 10 mL), stirring at 40–45 °C for 20 h, aqueous sodium hydroxide (1 M, 2 mL), purification by column chromatography (20×1 cm, SiO_2_, ethyl acetate/petroleum ether 3 : 1), 5,7‐bis (dibenzo[*b,d*]thiophen‐4‐yl)‐2,3‐dihydropyrrolo[2,1*‐b*]quinazolin‐9(1*H*)‐one (**15**, 124 mg, 0.23 mmol, 75 %), yellow crystals, m. p. 195–196 °C.

*R*_f_=0.68 (ethyl acetate/petroleum ether 3 : 1). ^1^H NMR (400 MHz, CDCl_3_): *δ*=8.85 (1 H, d, *J*=2.2 Hz, OCCCH), 8.35 (1 H, d, *J*=2.2 Hz, OCCCHCC*H*), 8.18–8.24 (4 H, m, dibenzothiophenyl), 7.78–7.87 (2 H, m, dibenzothiophenyl), 7.58–7.68 (4 H, m, dibenzothiophenyl), 7.45–7.49 (4 H, m, dibenzothiophenyl), 4.25 (2 H, t, *J*=7.2 Hz, NCH_2_), 3.07 (2 H, t, *J*=7.8 Hz, NCH_2_CH_2_C*H*_2_), 2.25 (2 H, m, NCH_2_C*H*_2_) ppm. ^13^C{^1^H} NMR (100.6 MHz, CDCl_3_): *δ*=161.3 (OC), 159.5 (OC*C*), 146.7 (OCCCHC), 140.6 (N*C*C), 139.9 (OCC*C*H), 139.6 (OCCCHCCH*C*), 138.7 (CCCHC*C*H), 138.4 (NCN), 138.3 (SC*C*), 136.6 (SC*C*), 136.0 (S*C*C), 135.9 (SC*C*H), 135.8 (SC*C*H), 135.5 (SCCH*C*H), 135.3 (SCCH*C*H), 134.1 (SCCHCH*C*H), 129.1 (SCCHCH*C*H), 127.4 (SCCHCHCH*C*H), 127.0 (SCCHCHCH*C*H), 126.8 (SCCHCHCHCH*C*), 126.3 (SCCHCHCHCH*C*), 125.4 (SCCHCHCHCHC*C*), 124.6 (2 C, SCCHCHCHCHC*C*), 124.4 (SCCHCHCHCHCC*C*), 122.8 (SCCHCHCHCHCC*C*), 122.7 (SCCHCHCHCHCC*C*H), 121.9 (SCCHCHCHCHCC*C*H), 121.87 (SCCHCHCHCHCCCH*C*H), 121.82 (SCCHCHCHCHCCCH*C*H), 121.1 (SCCHCHCHCHCCCHCH*C*H), 121.0 (SCCHCHCHCHCCCHCH*C*H), 46.7 (NCH_2_), 32.8 (NCH_2_CH_2_ *C*H_2_), 19.7 (NCH_2_ *C*H_2_) ppm.

IR (ATR): ν=3059 (CH_2_), 1670 (C=O), 1604 (C=N), 1465 (C=C), 1415 (C−N) cm^−1^. EI−MS: *m/z* (%)=367 ([M‐dibenzothiophenyl]^+^, 10.5), 366 ([M‐dibenzothiophene]^+^, 89.5), 351 ([M‐dibenzothiophenyl−O]^+^, 100), 183 ([dibenzothiophenyl]^+^, 6.8). MS (TOF ES+): *m/*z (%)=551 ([M+H]^+^, 100), 552 ([M+2H]^+^, 37.4), 526 (26.4), 512 (8.8). HRMS: Calcd. for C_35_H_23_N_2_OS_2_ ([M+H]^+^) 551.1252; found 551.1236.

**5**‐**Bromo**‐**7**‐**(quinolin**‐**8**‐**yl)**‐**2,3**‐**dihydropyrrolo[2,1**‐**b]quinazolin**‐**9(1H)**‐**one** (**16**): GP; 5,7‐dibromo‐2,3‐dihydropyrrolo[2,1‐*b*]quinazoline‐9‐(1*H*)*‐*one (**5**, 103 mg, 0.3 mmol), 8‐quinolineboronic acid (121 mg, 0.70 mmol), sodium carbonate (79.5 mg, 0.75 mmol), palladium (II) acetate (7.2 mg, 0.03 mmol) in acetone/water (1 : 1, 10 mL), stirring at 40–45 °C for 23 h, aqueous sodium hydroxide (1 M, 2 mL), purification by column chromatography (20×1 cm, SiO_2_, ethyl acetate/petroleum ether 5 : 1), 5‐bromo‐7‐(quinolin‐8‐yl)‐2,3‐dihydropyrrolo[2,1‐b]quinazolin‐9(1H)‐one (**16**, 80 mg, 0.20 mmol, 68 %), yellow crystals, m. p. 359–360 °C.

*R*_f_=0.68 (ethyl acetate/petroleum ether 3 : 1). ^1^H NMR (500 MHz, CDCl_3_): *δ*=8.96 (1 H, dd, *J*=1.2, 2.7 Hz, C_quin_H), 8.57 (1 H, d, *J*=1.4 Hz, OCCCH), 8.47 (1 H, d, *J*=1.3, OCCCHCC*H*), 8.23 (1 H, dd, *J*=1.2, 5.5 Hz, C_quin_H), 7.89 (1 H, dd, *J*=0.9, 5.4 Hz, C_quin_H), 7.82 (1 H, dd, *J*=1.0, 4.8, C_quin_H), 7.64 (1 H, t, *J*=4.8 Hz, C_quin_H), 7.46 (1 H, dd, *J*=2.7, 5.5, C_quin_H), 4.24 (2 H, t, *J*=4.8, NCH_2_), 3.31 (2 H, t, *J*=5.2 Hz, NCH_2_C*H*_2_), 2.33 (2 H, m, NCH_2_C*H*_2_CH_2_) ppm. ^13^C{^1^H} NMR (125 MHz, CDCl_3_): *δ*=160.7 (OC), 160.5 (OC*C*), 150.7 (OCCCHCCH*C*), 146.3 (N*C*CCH), 145.8 (OCC*C*H), 140.6 (CBr), 138.6 (BrC*C*H), 138.2 (NCN), 136.5 (BrCCHC*C*), 130.7 (BrCCHCC*C*H), 128.9 (BrCCHCC*C*N), 128.7 (NCHCHCH*C*), 127.7 (N*C*HCHCHC), 126.5 (NCCCH*C*H), 121.9 (NCH*C*HCHC), 121.5 (NCHCH*C*HC), 121.2 (NCHCHCHC*C*H), 46.9 (NCH_2_), 33.1 (NCH_2_CH_2_ *C*H_2_), 19.8 (NCH_2_ *C*H_2_) ppm. IR (ATR): ν=2987 (CH_2_), 1668 (C=O), 1600 (C=N), 1471 (C=C), 1406 (C−N), 748 (C−Br) cm^−1^. EI‐MS: *m/z* (%)=392 ([M−H]^+^, 50), 391 ([M]^+^, 93), 390 ([M−H]^+^, 100), 312 ([M−^79^Br]^+^, 82.8), 310 ([M−^81^Br]^+^, 9.0), 155 (11.3). HRMS calcd. for C_20_H_14_BrN_3_O 391.0320; found 391.0337.

**2**‐**Phenyl**‐**6,7,8,9**‐**tetrahydro**‐**11*H***‐**pyrido[2,1**‐***b*** **]quinazolin**‐**11**‐**one** (**17**): GP; 2‐bromo‐6,7,8,9‐tetrahydro‐11*H*‐pyrido[2,1‐*b*]quinazolin‐11‐one (**4**, 139.5 mg, 0.5 mmol), phenylboronic acid (67 mg, 0.56 mmol), sodium carbonate (132.5 mg, 1.25 mmol) and palladium (II) acetate (9.43 mg, 0.042 mmol) in acetone/water (1 : 1, 6 mL) were stirred at 40–45 °C for 3 h. Column chromatography (20×1 cm, SiO_2_, ethyl acetate/petroleum ether 10 : 1) gave 2‐phenyl‐6,7,8,9‐tetrahydro‐11*H*‐pyrido[2,1‐*b*]quinazolin‐11‐one (**17**, 124 mg, 0.45 mmol, 90 %), colorless crystals (m. p. 143–144 °C).

*R*_f_=0.62 (benzene/methanol 5 : 1). ^1^H NMR (400 MHz, CDCl_3_): *δ*=8.49 (1 H, d, *J*=2.4 Hz, OCCCH), 7.98 (1 H, dd, *J*=2.4, 8.0 Hz, NCCHC*H*), 7.66–7.71 (3 H, m, *m*‐CH, *p*‐CH), 7.44–7.49 (2 H, m, *o*‐CH), 7.37 (1 H, d, *J*=8.0 Hz, NCCH), 4.10 (2H, t, *J*=6.1 Hz, NCH_2_), 3.01 (2 H, t, *J*=6.7 Hz, NCCH_2_), 1.95–2.05 (4 H, m, NCH_2_C*H*_2_, NCH_2_CH_2_C*H*_2_) ppm. ^13^C{^1^H} NMR (100.6 MHz, CDCl_3_): *δ*=162.4 (CO), 155.0 (OCC*C*H), 146.8 (OC*C*), 139.9 (OCCCH*C*), 139.1 (N*C*CH), 133.3 (N*C*N), 129.1 (OCCCHC*C*H), 127.8 (OCCCHC*C*), 127.3 (NC*C*H), 127.1 (*o*‐CH), 124.6 (*m*‐CH), 120.8 (*p*‐CH), 42.5 (NCH_2_CH_2_ *C*H_2_), 32.1 (NCH_2_ *C*H_2_), 22.3 (NCH_2_CH_2_CH_2_ *C*H_2_), 19.5 (NCH_2_) ppm. IR (ATR): ν=1654 (C=O), 1600 (C=N), 1473 (C−N) cm^−1^. EI−MS: *m/z* (%)=276 ([M]^+^, 100), 275 ([M−H]^+^, 15), 261 ([M−H−CH_2_]^+^, 4.4), 220 ([M−H−(CH_2_)_3_−CH_3_]^+^, 2.5), 199 ([M−C_6_H_5_]^+^, 2.3), 185 ([M−C_6_H_5_−CH_2_]^+^, 2.4). HRMS: Calcd. for C_18_H_16_N_2_O 276.1263; found: 276.1264.

**4**‐**(11**‐**Oxo**‐**6,8,9,11**‐**tetrahydro**‐**7*H***‐**pyrido[2,1**‐**b]quinazolin**‐**2**‐**yl) benzonitrile** (**18**): GP; 2‐bromo‐6,7,8,9‐tetrahydro‐11*H*‐pyrido[2,1‐*b*]quinazolin‐11‐one (**4**, 139.5 mg, 0.5 mmol), (4‐cyanophenyl) boronic acid (81.0 mg, 0.55 mmol), sodium carbonate (132.5 mg, 1.25 mmol) and palladium (II) acetate (9.43 mg, 0.042 mmol) in acetone / water (2 : 1, 6 mL) were stirred at 40–45 °C for 3 h. Column chromatography (20×1 cm, SiO_2_, ethyl acetate) gave 4‐(11‐oxo‐6,8,9,11‐tetrahydro‐7*H*‐pyrido[2,1‐b]quinazolin‐2‐yl) benzonitrile (**18**, 138.5 mg, 0.46 mmol, 92 %), colorless crystals (m. p. 194–195 °C).

*R*_f_=0.36 (ethyl acetate^1^H NMR (400 MHz, CDCl_3_): *δ*=8.49 (1 H, d, *J*=2.2 Hz, OCCCH), 7.95 (1 H, dd, *J*=2.3, 8.5 Hz, NCCHC*H*), 7.68–7.79 (5 H, m, NCCH, *o*‐CH, *m*‐CH), 4.11 (2 H, t, *J*=6.2 Hz, NCH_2_), 3.03 (2 H, t, *J*=6.7 Hz, NCCH_2_), 1.95–2.07 (4 H, m, NCH_2_C*H*_2_, NCH_2_CH_2_C*H*_2_) ppm. ^13^C{^1^H} NMR (100.6 MHz, CDCl_3_): *δ*=162.2 (CN), 155.9 (CO), 147.7 (NCH_2_), 144.3 (OC*C*), 136.8 (OCCCH*C*), 132.92 (*m*‐CH), 127.8 (*o*‐CH), 127.6 (OCCCHC*C*), 125.3 (NCN), 121.0 (NCCH*C*H), 118.9 (NC*C*H), 111.4 (N*C*CH), 42.7 (NCH_2_), 32.2 (NCH_2_CH_2_CH_2_ *C*H_2_), 22.2 (NCH_2_ *C*H_2_), 19.4 (NCH_2_CH_2_ *C*H_2_) ppm. IR (ATR): ν=2220 (CN), 1661 (C=O), 1583 (C=N), 1492 (C−N) cm^−1^. MS (TOF ES+): *m/*z (%)=303 ([M+2H]^+^, 16.5), 302 ([M+H]^+^, 100), 287 ([M−CH_2_]^+^, 1.3), 261 ([M−C_3_H_4_ (cyclopropene)]^+^, 1.3), 145 (4‐oxo‐3,4‐dihydroquinazolin‐1‐ium, 4.9). HRMS: Calcd. for C_19_H_15_N_3_O 302.1293; found 302.1465.

Crystal structure analysis:^[49]^ CCDC 1994934. Single crystals suitable for X‐ray crystallographic analysis were obtained by crystallization from ethyl acetate at 25 °C. C_19_H_15_N_3_O, colorless prism, M_r_=301.34 g . mol^−1^, crystal system monoclinic, space group C2/c, *a*=16.456(3), *b*=6.814(1), *c*=27.286(6) Å, *α*=90°, *β*=99.65(3)°, γ=90°. V=3016.5(11) Å^3^, Z=8, d_calc_=1.327 g cm^−3^, m=0.674 mm^−1^, crystal size 0.48×0.44×0.31 mm^3^, F(000)=1264, CCD Xcalibur diffractometer, graphite monochromator, T=291(2) K, Mo−K_*α*_ radiation (*λ*=0.71073 Å), 3.29≤θ≤75.78°, index ranges −20≤h≤10, −8≤k≤8, −34≤l≤32, reflections collected/unique 6013/3064, multi‐scan absorption correction, structure solution and refinement with SHELXL,^[57]^ parameters/restrains 227/0, R_1_=0.0505 [l>2 s(l)], wR_2_=0.1398 (all data all data R_1_=0.0610, wR_2_=0.1536), S=1.031, final maximum/minimum difference electron density 0.184/−0.262 eÅ^−3^.

**2**‐**(3,4,5**‐**Trimethoxyphenyl)**‐**6,7,8,9**‐**tetrahydro**‐**11*H***‐**pyrido[2,1**‐***b*** **]quinazolin**‐**11**‐**one** (**19**): GP; 2‐bromo‐6,7,8,9‐tetrahydro‐11*H*‐pyrido[2,1‐*b*]quinazolin‐11‐one (**4**, 139.5 mg, 0.5 mmol), **(**3,4,5‐trimethoxyphenyl)boronic acid (127.2 mg, 0.6 mmol), sodium carbonate (132.5 mg, 1.25 mmol) and palladium (II) acetate (9.43 mg, 0.042 mmol) in acetone/water (2 : 1, 6 mL) were stirred at 40–45 °C for 3 h. Column chromatography (20×1 cm, SiO_2_, ethyl acetate / petroleum ether 6 : 1) gave 2‐(3,4,5‐trimethoxyphenyl)‐6,7,8,9‐tetrahydro‐11*H*‐pyrido[2,1‐*b*]quinazolin‐11‐one (**19**, 180 mg, 0.49 mmol, 98 %), yellow crystals (m. p. 160–161 °C).

*R*_f_=0.30 (ethyl acetate). ^1^H NMR (400 MHz, CDCl_3_): *δ*=8.43 (1H, d, *J*=1.9 Hz, OCCCH), 7.93 (1 H, dd, *J*=2.2, 8.5 Hz, NCCHC*H*), 7.65 (1 H, d, *J*=8.5 Hz, NCCH), 6.86 (2 H, s, *o*‐CH), 4.10 (2 H, t, *J*=6.1 Hz, NCH_2_), 3.94 (6 H, s, *m*‐OCH_3_), 3.89 (3 H, s, *p*‐OCH_3_), 3.02 (2 H, t, *J*=6.7 Hz, NCH_2_CH_2_CH_2_C*H*_2_), 1.97–2.07 (4 H, m, NCH_2_C*H*_2_, NCH_2_CH_2_C*H*_2_) ppm. ^13^C{^1^H} NMR (100.6 MHz, CDCl_3_): *δ*=162.4 (CO), 155.0 (OCCCH*C*), 153.7 (OC*C*CH), 146.7 (NCCH*C*H), 139.1 (OCC*C*H), 138.1 (NC*C*H), 135.8 (N*C*CH), 133.2 (NCN), 127.0 (*o*‐CH), 124.4 (*m*‐CO), 120.7 (*p*‐CO), 104.5 (NCCHCHC*C*), 61.1 (*p*‐CO*C*H_3_), 56.4 (*o*‐CO*C*H_3_), 42.6 (NCH_2_CH_2_ *C*H_2_), 32.1 (NCH_2_ *C*H_2_), 22.2 (NCH_2_CH_2_CH_2_ *C*H_2_), 19.5 (NCH_2_) ppm. IR (ATR): *ν*=1664 (C=O), 1616 (C=N), 1473 (C−N) cm^−1^. TOF EI‐MS: *m/z* (%)=367 ([M+H]^+^, 8.4), 366 ([M]^+^, 100), 351 ([M−CH_3_]^+^, 97), 323 (9.5), 293 (4.2), 237 (5.3), 184 (3.1). HRMS: Calcd. for C_21_H_22_N_2_O_4_ 366.1580; found: 366.1576.

Crystal structure analysis:^[49]^ CCDC 2344455. Single crystals suitable for X‐ray crystallographic analysis were obtained by crystallization from ethyl acetate at 25 °C. C_21_H_22_N_2_O_4_, yellow prism, M_r_=366.40 g . mol^−1^, crystal system monoclinic, space group P 1 21/c 1, *a*=14.077(2), *b*=8.7947(14), *c*=15.364(2) Å, *α*=90°, *β*=108.393(5)°, *γ*=90°. V=1804.9(5) Å^3^, Z=4, d_calc_=1.348 g cm^−3^, *μ*=0.094 mm^−1^, crystal size 0.62×0.38×0.35 mm^3^, F(000)=776, Bruker SMART X2S diffractometer, graphite crystal monochromator, T=200(2) K, Mo−K_*α*_ radiation (λ=0.71073 Å), 2.71≤θ≤27.54°, index ranges −18≤h≤18, −11≤k≤11, −14≤l≤19, reflections collected/unique 4156/2746, multi‐scan absorption correction, structure solution and refinement with SHELXL,^[57]^ parameters/restrains 248/0, R_1_=0.0600 [l>2 s(l)], wR_2_=0.1676, S=1.056, final maximum/minimum difference electron density 0.634/−0.321 eÅ^−3^.

**2**‐**(Benzo[*b*]thiophen**‐**3**‐**yl)**‐**6,7,8,9**‐**tetrahydro**‐**11*H***‐**pyrido[2,1**‐**b]quinazolin**‐**11**‐**one** (**20**): GP; 2‐bromo‐6,7,8,9‐tetrahydro‐11*H*‐pyrido[2,1‐*b*]quinazolin‐11‐one (**4**, 139.5 mg, 0.5 mmol), benzothiophenyl‐3‐boronic acid (106.8 mg, 0.6 mmol), sodium carbonate (132.5 mg, 1.25 mmol) and palladium(II) acetate (9.43 mg, 0.042 mmol) in acetone / water (2 : 1, 6 mL) were stirred at 40–45 °C fo 3 h. Column chromatography (20×1 cm, SiO_2_, ethyl acetate/petroleum ether 6 : 1) gave 2‐(benzo[*b*]thiophen‐3‐yl)‐6,7,8,9‐tetrahydro‐11*H*‐pyrido[2,1‐*b*]quinazolin‐11‐one (**20**, 158 mg, 0.47 mmol, 95 %), colorless crystals (m. p. 320 °C).

*R*_f_=0.34 (ethyl acetate/petroleum ether 6 : 1). ^1^H NMR (400 MHz, CDCl_3_): *δ*=8.49 (1 H, d, *J*=2.0 Hz, OCCCH), 7.91–7.97 (3 H, m, NCCHC*H*, SCCH, SCCHCHC*H*), 7.71 (1 H, d, *J*=8.4 Hz, NCCH), 7.51 (1 H, s, SCH), 7.39–7.42 (2 H, m, SCCHC*H*, SCCHCHCHC*H*), 4.11 (2 H, t, *J*=6.1 Hz, NCH_2_), 3.04 (2 H, t, *J*=6.7 Hz, NCH_2_CH_2_CH_2_C*H*_2_), 1.97–2.07 (4 H, m, NCH_2_C*H*_2_, NCH_2_CH_2_C*H*_2_) ppm. ^13^C{^1^H} NMR (100.6 MHz, CDCl_3_): *δ*=162.3 (CO), 155.2 (OCCCH*C*), 146.9 (OCCCHC*C*), 140.9 (OCC*C*H), 137.7 (SCH), 136.9 (OC*C*CH), 134.8 (N*C*CH), 134.0 (NCCH*C*H), 127.0 (NCN), 126.3 (S*C*CH, SC*C*CH), 124.7 (SCCHCHCH*C*H), 124.4 (SC*C*H), 123.1 (SCCH*C*H), 122.8 (SCCHCH*C*H), 120.8 (NC*C*H), 42.6 (NCH_2_CH_2_ *C*H_2_), 32.1 (NCH_2_ *C*H_2_), 22.3 (NCH_2_CH_2_CH_2_ *C*H_2_), 19.5 (NCH_2_) ppm. IR (ATR): *ν*=1654 (C=O), 1618 (C=N), 1483 (C−N). EI‐MS: *m/z* (%)=333 ([M+H]^+^, 44), 332 ([M]^+^, 100), 317, 195, 158. HRMS: Calcd. for C_20_H_16_N_2_OS 332.0983; found: 332.0985.

**2**‐**(Dibenzo [*b,d*] thiophen**‐**4**‐**yl)**‐**6,7,8,9**‐**tetrahydro**‐**11*H***‐**pyrido[2,1**‐***b*** **]quinazolin**‐**11**‐**one** (**21**): GP; 2‐bromo‐6,7,8,9‐tetrahydro‐11*H*‐pyrido[2,1‐*b*]quinazolin‐11‐one (**4**, 139.5 mg, 0.5 mmol), dibenzothiophenyl‐4‐boronic acid (137 mg, 0.6 mmol), sodium carbonate (132.5 mg, 1.25 mmol) and palladium (II) acetate (9.43 mg, 0.042 mmol) in acetone / water (2 : 1, 6 mL) were stirred at 40–45 °C for 7 h. Column chromatography (20×1 cm, SiO_2_, ethyl acetate/petroleum ether 4 : 1) gave 2‐(dibenzo[*b,d*]thiophen‐4‐yl)‐6,7,8,9‐tetrahydro‐11H‐pyrido[2,1‐b]quinazolin‐11‐one (**21**, 180 mg, 0.47 mmol, 94 %), colorless crystals (m. p. 177–179 °C).

*R*_f_=0.13 (ethyl acetate). ^1^H NMR (400 MHz, CDCl_3_): *δ*=8.65 (1 H, d, *J*=2.1 Hz, OCCC*H*), 8.17–8.21 (2 H, m, SCCHC*H*, SCCHCHC*H*), 8.13 (1 H, dd, *J*=8.4, J=2.2 Hz, NCCHC*H*), 7.81–7.84 (1 H, m, SCCCHC*H*), 7.75 (1 H, d, *J*=8.4 Hz, NCC*H*), 7.55–7.60 (2 H, m, SCCHCHCHC*H*, SCCCHCHC*H*), 7.44–7.49 (2 H, m, SCC*H*, SCCC*H*), 4.11 (2 H, t, *J*=6.1 Hz, NCH_2_), 3.04 (2 H, t, *J*=6.6 Hz, NCH_2_CH_2_CH_2_C*H*_2_), 1.95–2.08 (4H, m, NCH_2_C*H*_2_, NCH_2_CH_2_C*H*_2_) ppm. ^13^C{^1^H} NMR (100.6 MHz, CDCl_3_): *δ*=162.3 (CO), 155.4 (OCCCH*C*), 147.2 (OCCCHC*C*), 139.6 (OCC*C*H), 138.7 (SC*C*CCH) 138.5 (S*C*CH), 136.5 (OC*C*CH), 135.8 (N*C*CH), 135.8 (NCCH*C*H), 134.3 (NCN), 127.2 (SCCHCHCHCH*C*), 127.1 (SCCHCHCHCHC*C*), 127.0 (SCCHCHCHCHCC*C*), 126.3 (SC*C*H), 125.3 (SCCCH*C*H), 124.6 (SCCH*C*H), 122.8 (SCCHCH*C*H), 121.9 (SCCHCHCH*C*H), 121.0 (SCCCHCH*C*H), 120.8 (NC*C*H), 42.6 (NCH_2_CH_2_ *C*H_2_), 32.1 (NCH_2_ *C*H_2_), 22.2 (NCH_2_CH_2_CH_2_ *C*H_2_), 19.5 (NCH_2_) ppm. IR (ATR): *ν*=1672 (C=O), 1600 (C=N), 1458 (C−N) cm^−1^. EI‐MS: *m/z* (%)=383 ([M+H]^+^, 12.6), 382 ([M]^+^, 100), 191 ([M]^2+^ or 4‐oxo‐3‐propyl‐1,2,3,4‐tetrahydroquinazolin‐1‐ium, 4). HRMS: Calcd. for C_24_H_18_N_2_OS 382.1140; found: 382.1142.

## Supporting Information Summary

^1^H and ^13^C{^1^H} NMR spectra of compounds **3**–**5** as well as of the synthesized new compounds are available in the Supporting Information.

## Conflict of Interests

The authors declare no conflict of interest.

1

## Supporting information

As a service to our authors and readers, this journal provides supporting information supplied by the authors. Such materials are peer reviewed and may be re‐organized for online delivery, but are not copy‐edited or typeset. Technical support issues arising from supporting information (other than missing files) should be addressed to the authors.

Supporting Information

## Data Availability

NMR spectra of compounds **3–5** as well as of new compounds are available in the Supporting Information of this article. Crystal structure analyses were deposited with the CCDC.
