# Supplementary material for: New Tricyclic Aryl Quinazoline Derivatives by Suzuki‐Miyaura Cross‐Coupling
Source: ChemistryOpen. 2024 Sep 27;13(12):e202400197. doi: 10.1002/open.202400197 (PMC11625927; doi:10.1002/open.202400197)

ChemistryOpen

Supporting Information

New Tricyclic Aryl Quinazoline Derivatives by Suzuki-Miyaura Cross-Coupling

Burkhon Elmuradov,* Rasul Okmanov, Bakhromjon Juraev, Gerald Dräger, and Holger Butenschön*

New Tricyclic Quinazoline Derivatives by Suzuki-Miyaura Cross-Coupling

Burkhon Elmuradov,[a,b]* Rasul Okmanov,[b] Bakhromjon Juraev,[b] Gerald Dräger[a] and Holger Butenschön[a]*

[a] Leibniz Universität Hannover, Institut für Organische Chemie, Schneiderberg 1B, D-30167 Hannover, Germany

[b] Institute of the Chemistry of Plant Substances, Academy of Sciences of Uzbekistan, 100170, Mirzo-Ulugbek str. 77, Tashkent, Uzbekistan

Supporting Information

- NMR spectra of starting materials **3**-**5** and of new compounds

7-Bromo-2,3-dihydropyrrolo[2,1-*b*]quinazolin-9(1*H*)-one (7-bromodeoxyvasicinone, 3):

^{1}H NMR (400 MHz, $CDCl_3$):

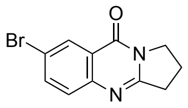

3

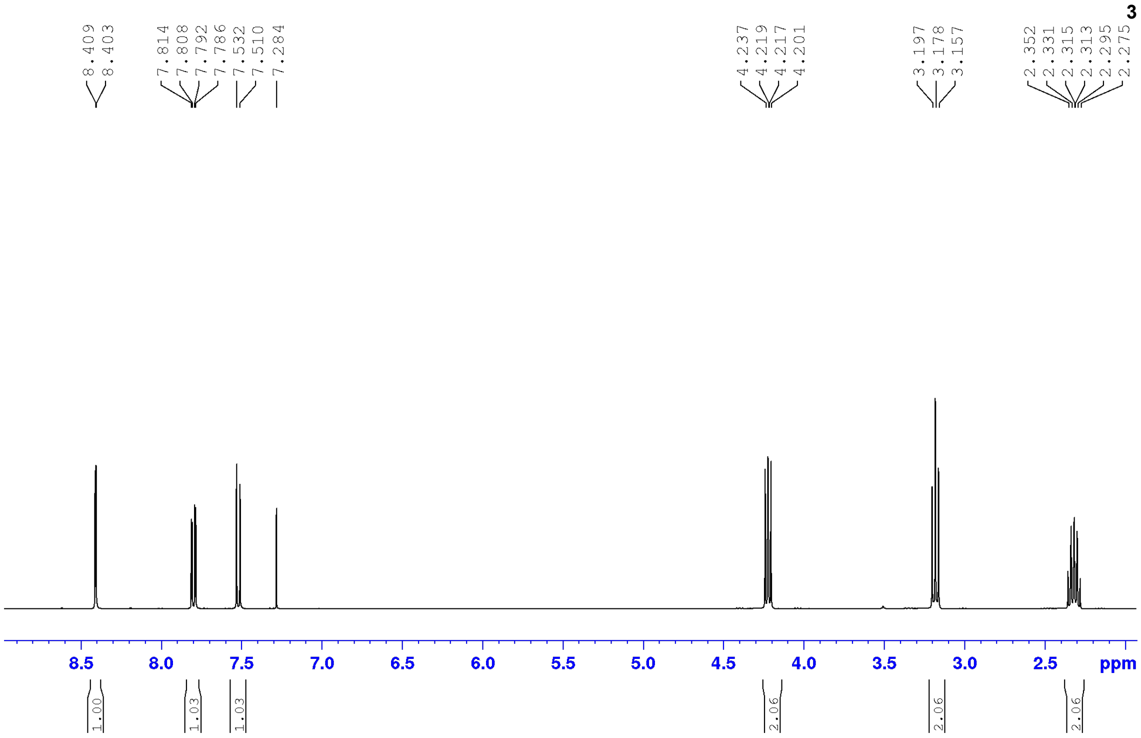

^{13}C{^{1}H} NMR (100.6 MHz, $CDCl_3$):

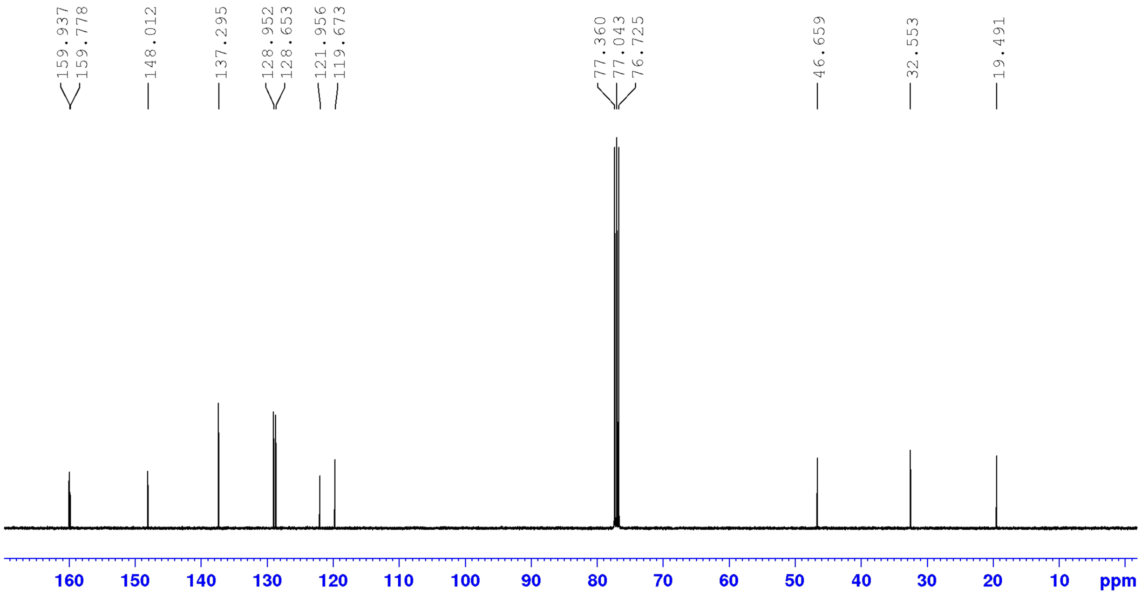

2-Bromo-6,7,8,9-tetrahydro-11*H*-pyrido[2,1-*b*]quinazolin-11-one (2-bromo-mackinazolin–one, 4):

^{1}H NMR (600 MHz, $CDCl_3$):

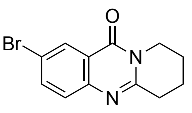

4

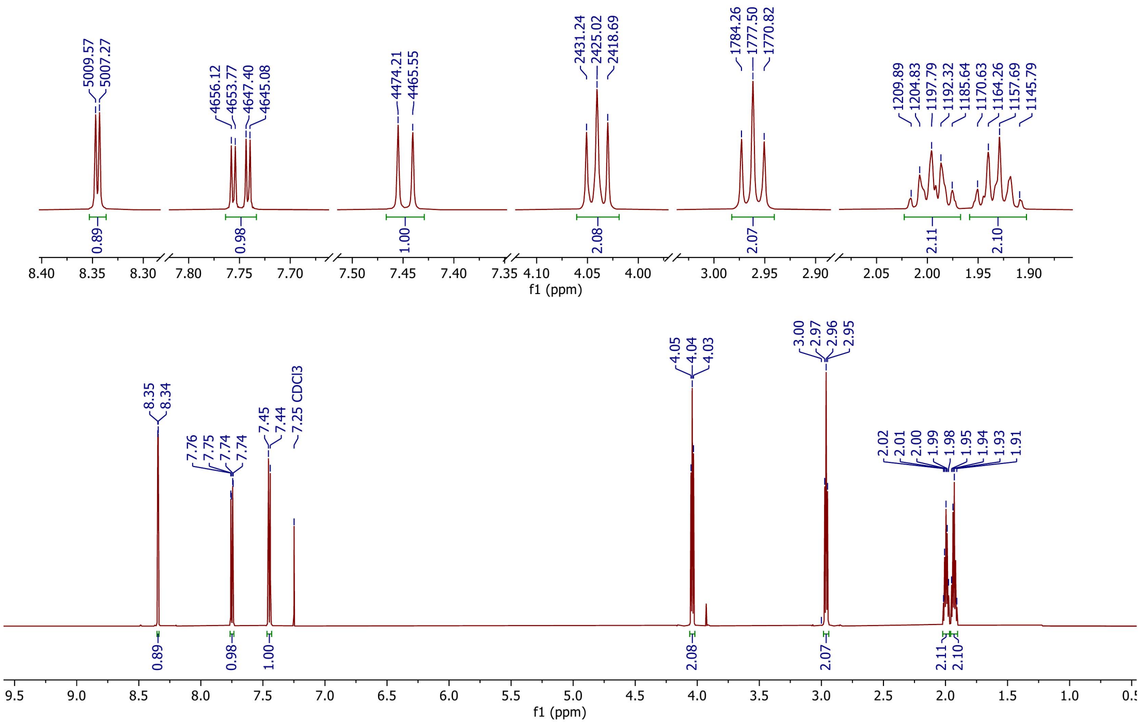

^{13}C{^{1}H} NMR (150 MHz, $CDCl_3$):

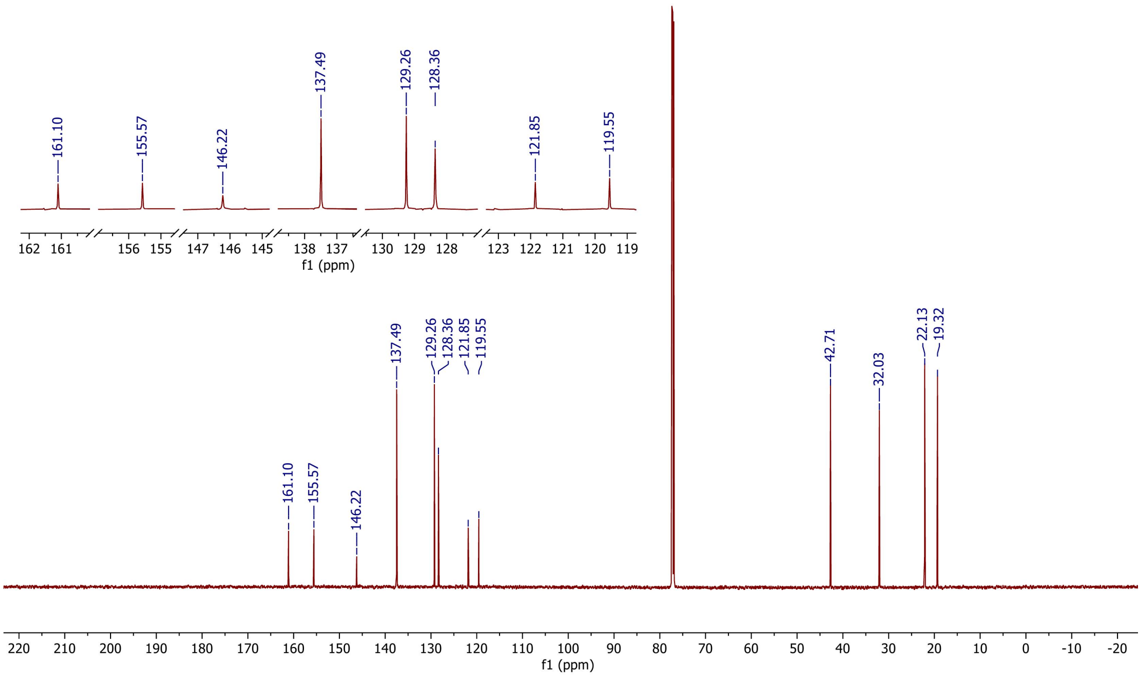

5,7-Dibromo-2,3-dihydropyrrolo[2,1-*b*]quinazolin-9(1*H*)-one (5,7-dibromodeoxyvasicinone, 5):

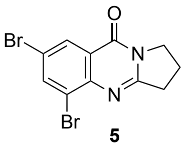

5

^{1}H NMR (600 MHz, $CDCl_3$):

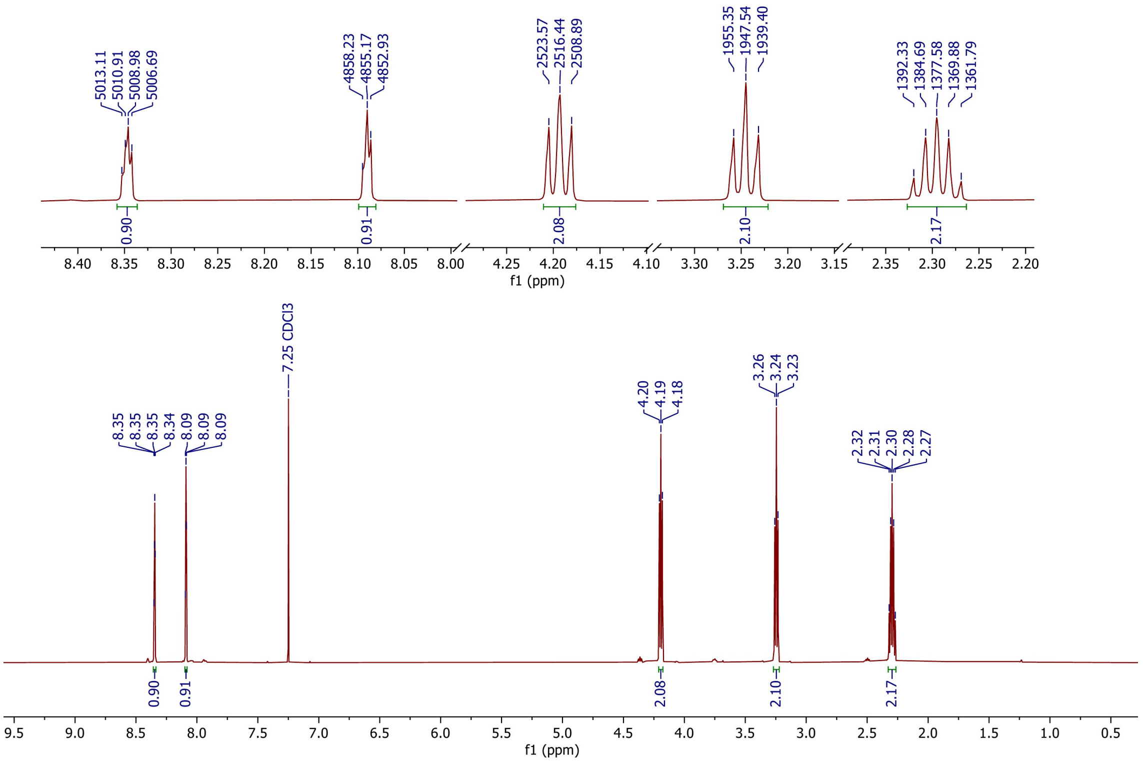

^{13}C{^{1}H} NMR (150 MHz, $CDCl_3$):

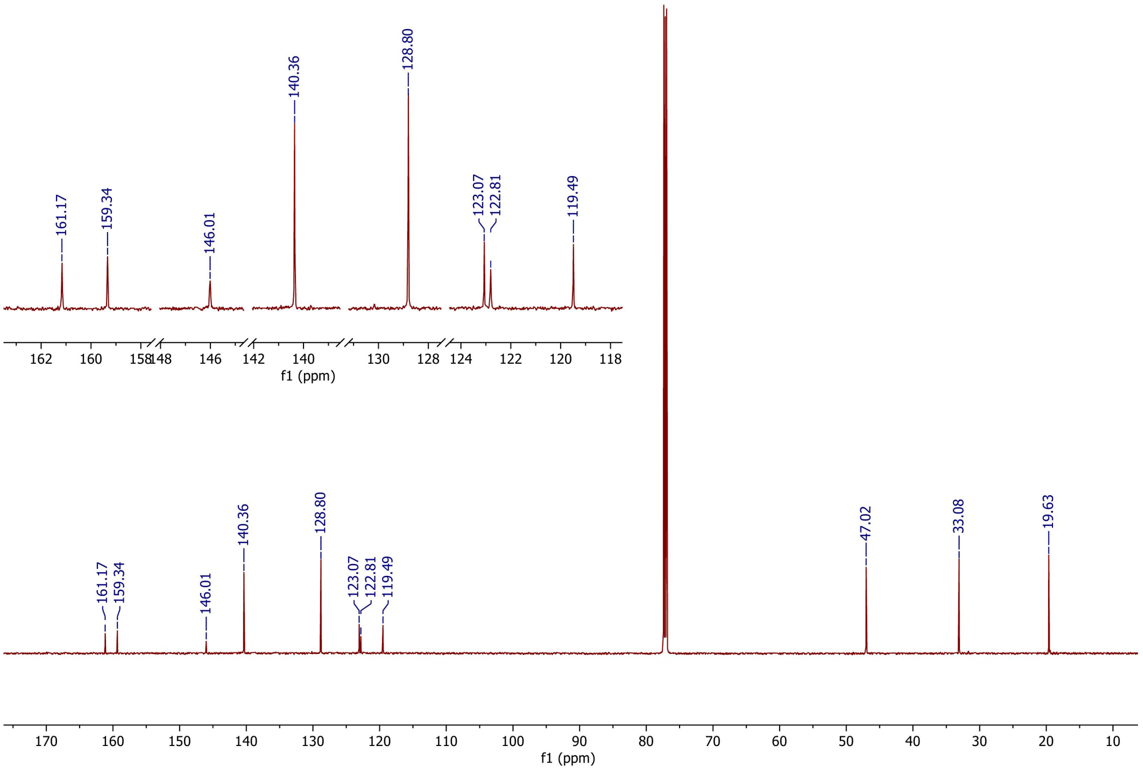

7-Phenyl-2,3-dihydropyrrolo[2,1-*b*]quinazolin-9(1*H*)-one (6)

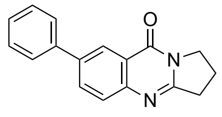

6

^{1}H NMR (400 MHz, $CDCl_3$):

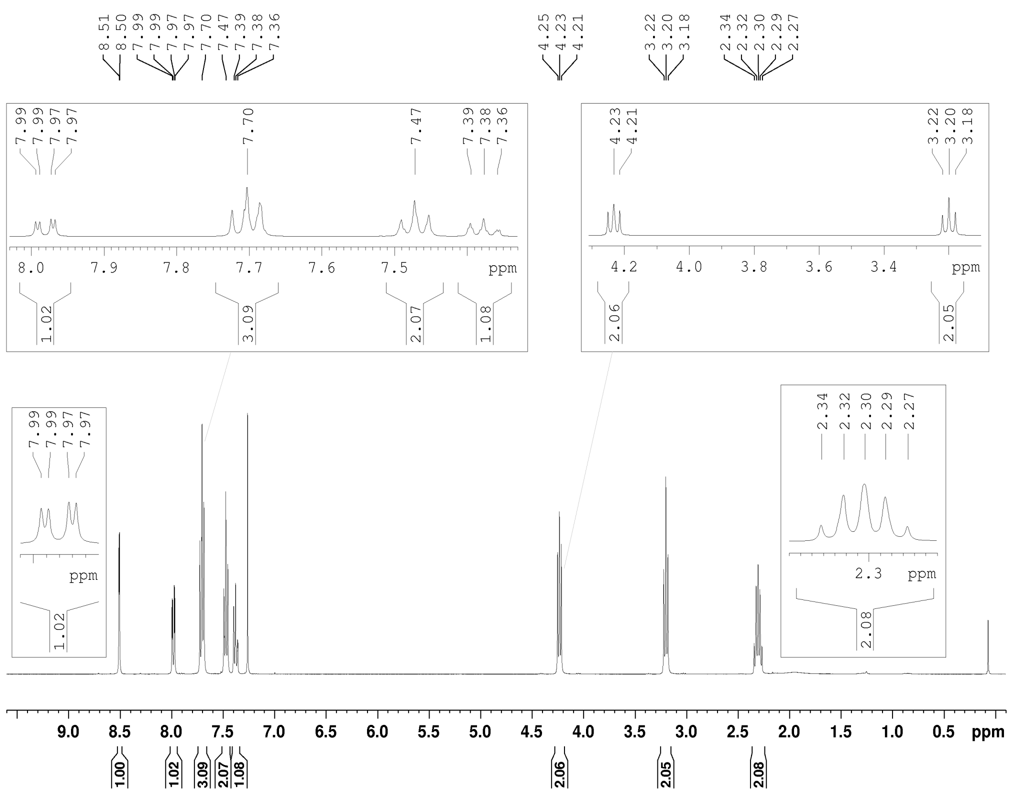

^{13}C{^{1}H} NMR (100.6 MHz, $CDCl_3$):

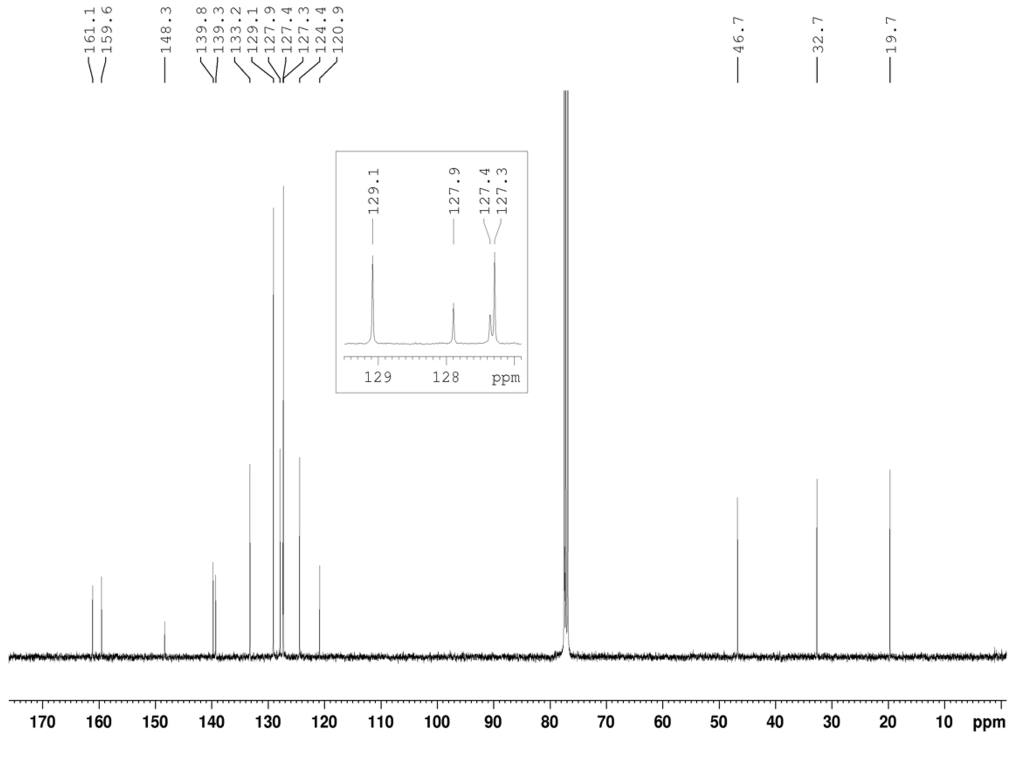

4-(9-Oxo-1,2,3,9-tetrahydropyrrolo[2,1-*b*]quinazolin-7-yl)benzonitrile (7)

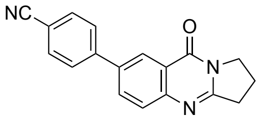

7

^{1}H NMR (400 MHz, $CDCl_3$):

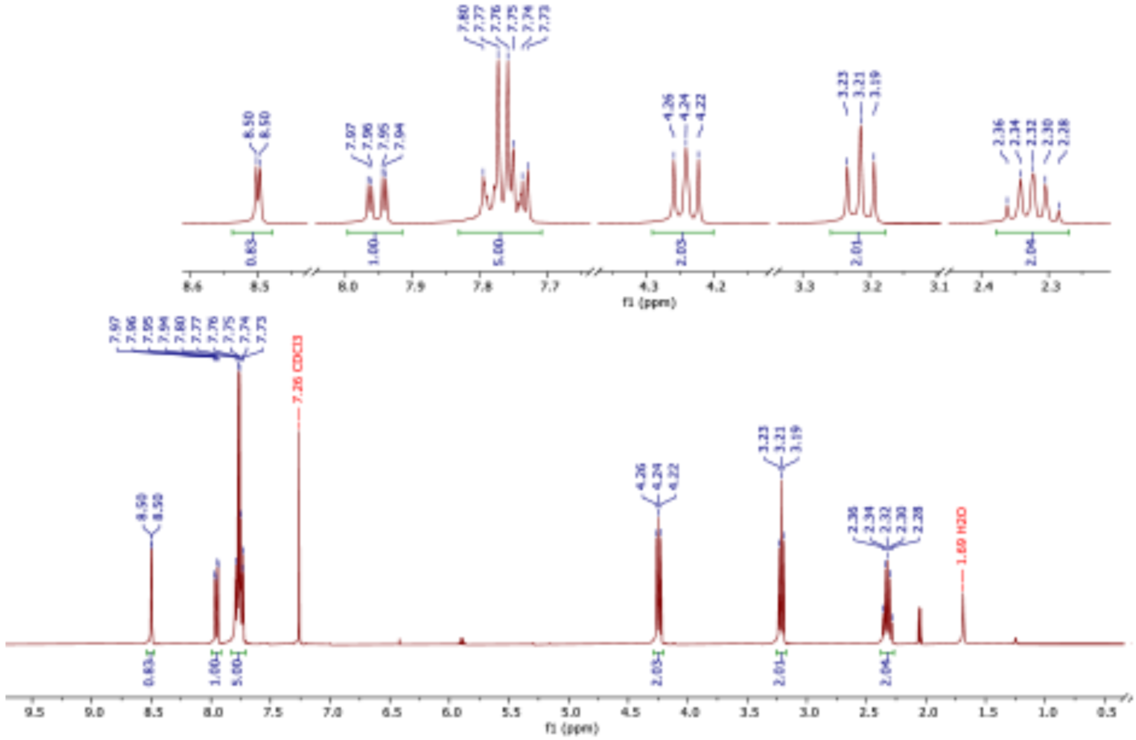

$^{13}C\{^{1}H\}$ NMR (100.6 MHz, $CDCl_3$):

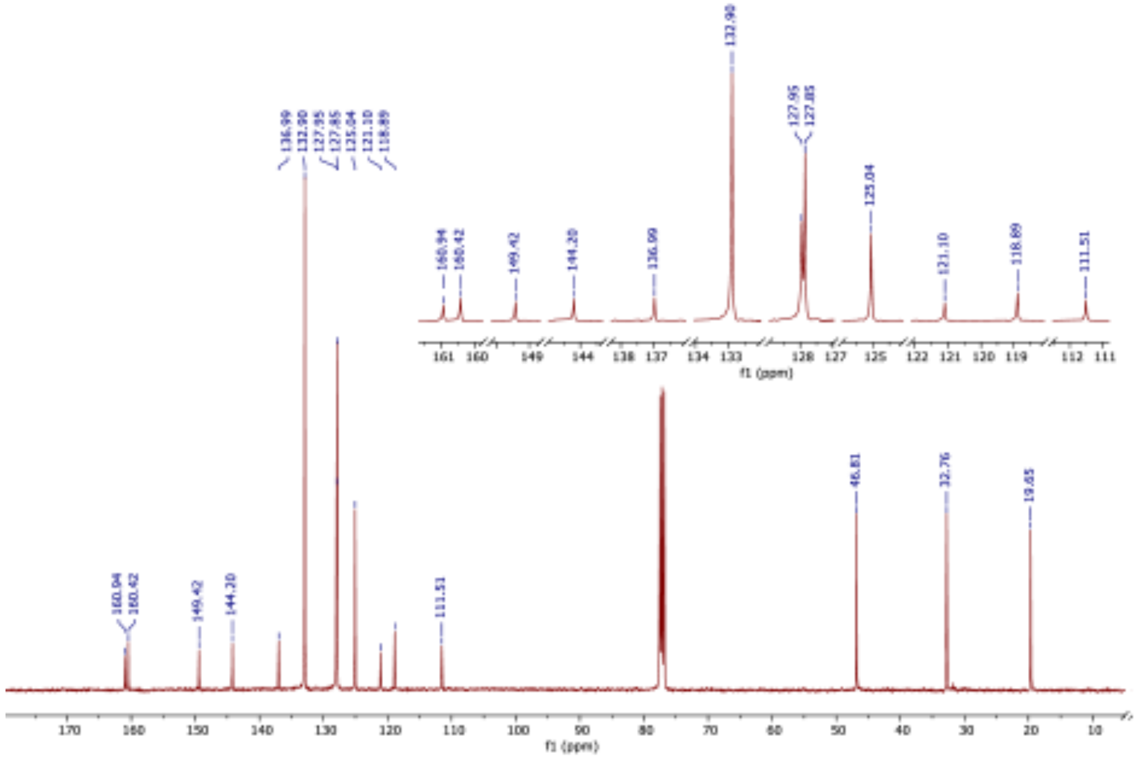

7-(Quinolin-8-yl)-2,3-dihydropyrrolo[2,1-*b*]quinazolin-9(1*H*)-one (8)

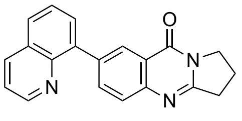

8

^{1}H NMR (400 MHz, CDCl$_3$):

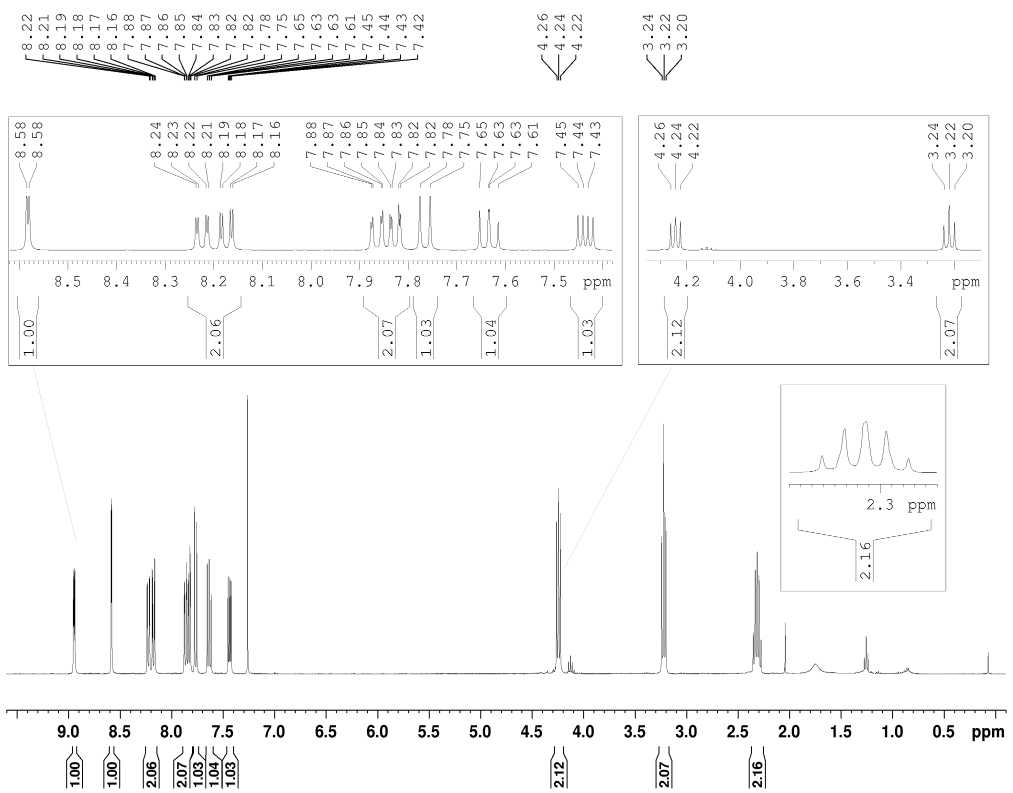

^{13}C{^{1}H} NMR (100.6 MHz, CDCl$_3$):

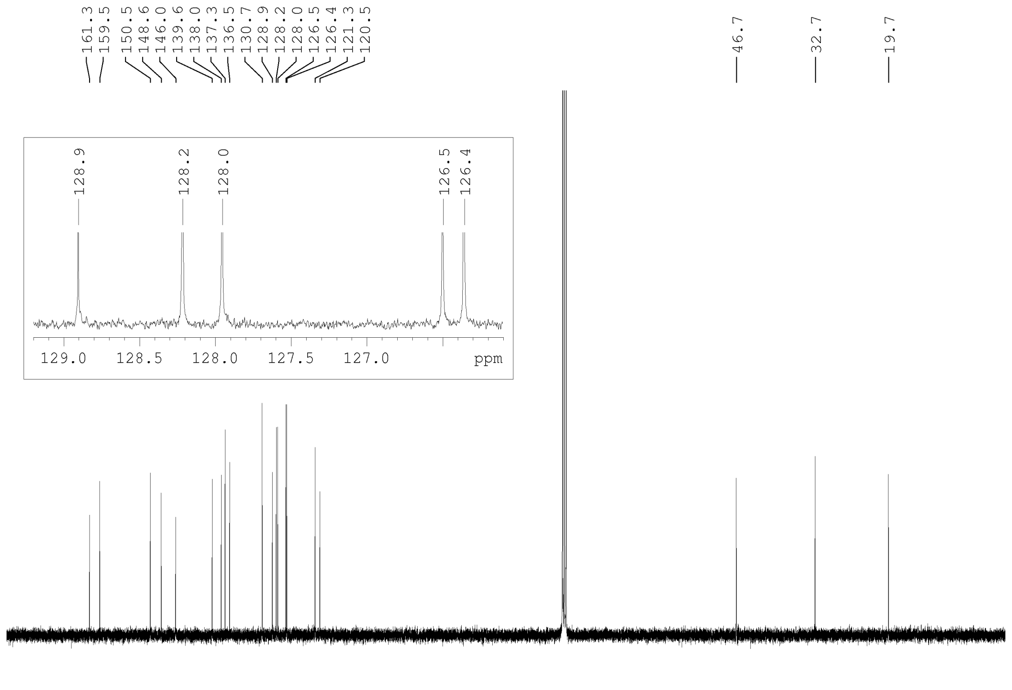

7-(3,4,5-Trimethoxyphenyl)-2,3-dihydropyrrolo[2,1-*b*]quinazolin-9(1*H*)-one (9)

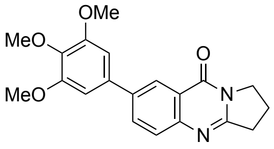

9

^{1}H NMR (400 MHz, CDCl$_3$):

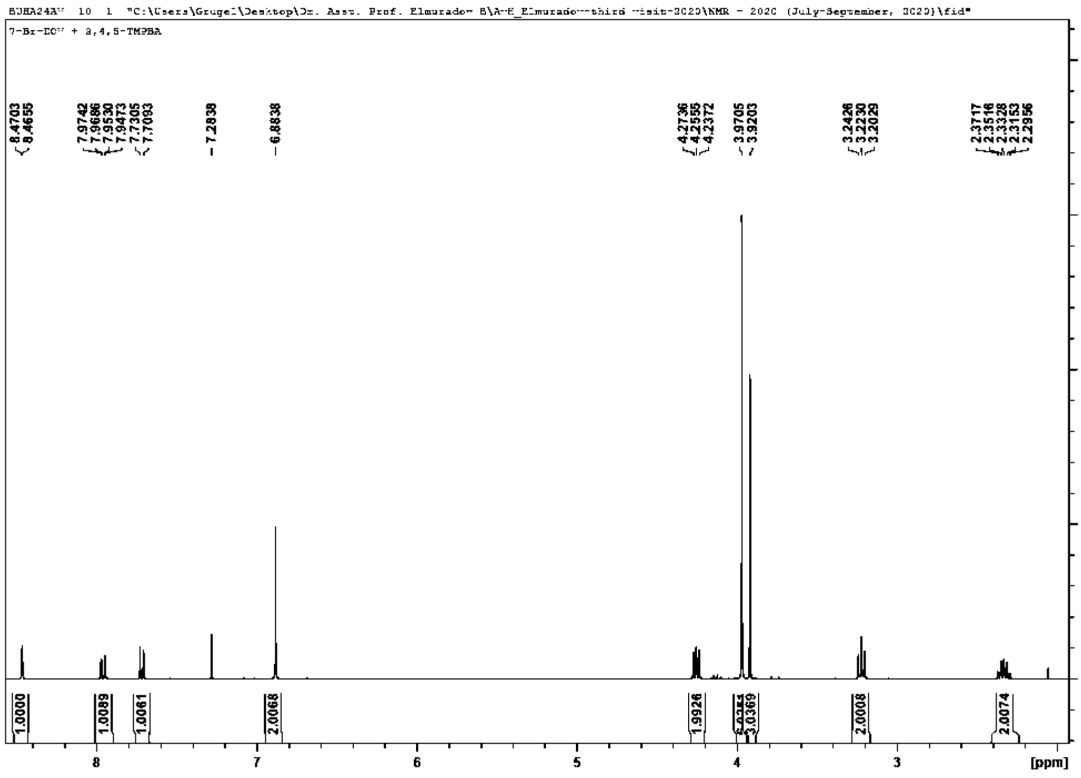

^{13}C{^{1}H} NMR (100.6 MHz, CDCl$_3$):

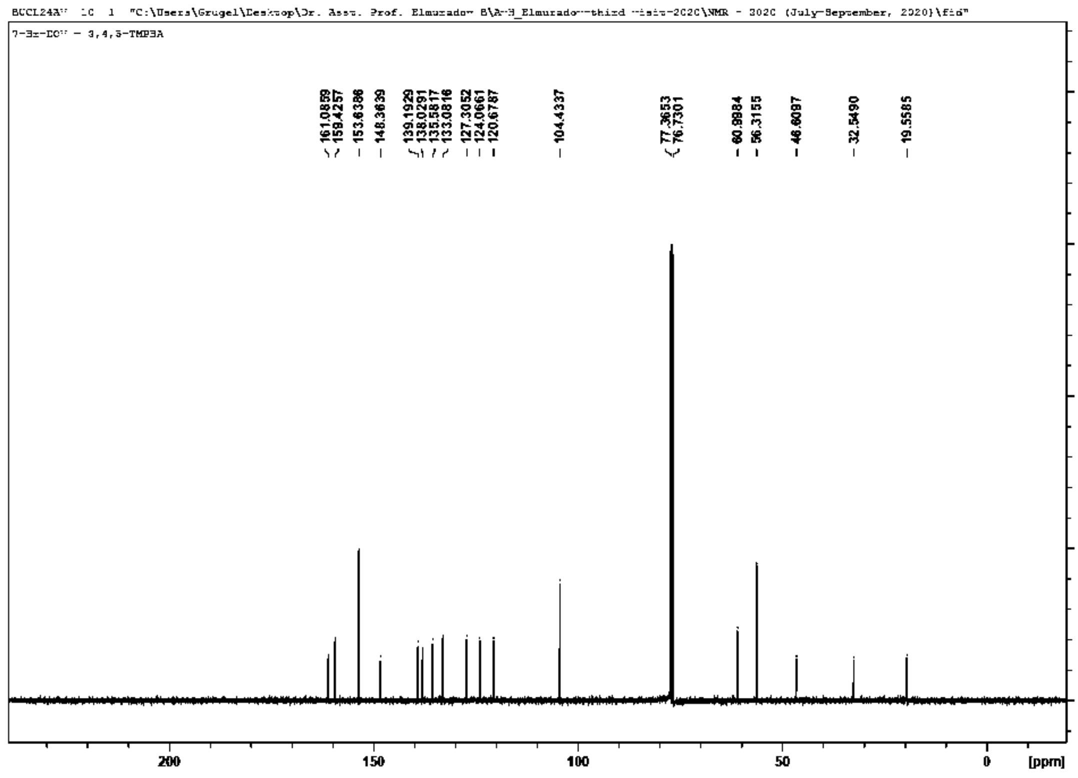

7-(Benzo[*b*]thiophen-3-yl)-2,3-dihydropyrrolo[2,1-*b*]quinazolin-9(1*H*)-one (10)

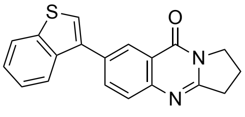

10

^{1}H NMR (400 MHz, $CDCl_3$):

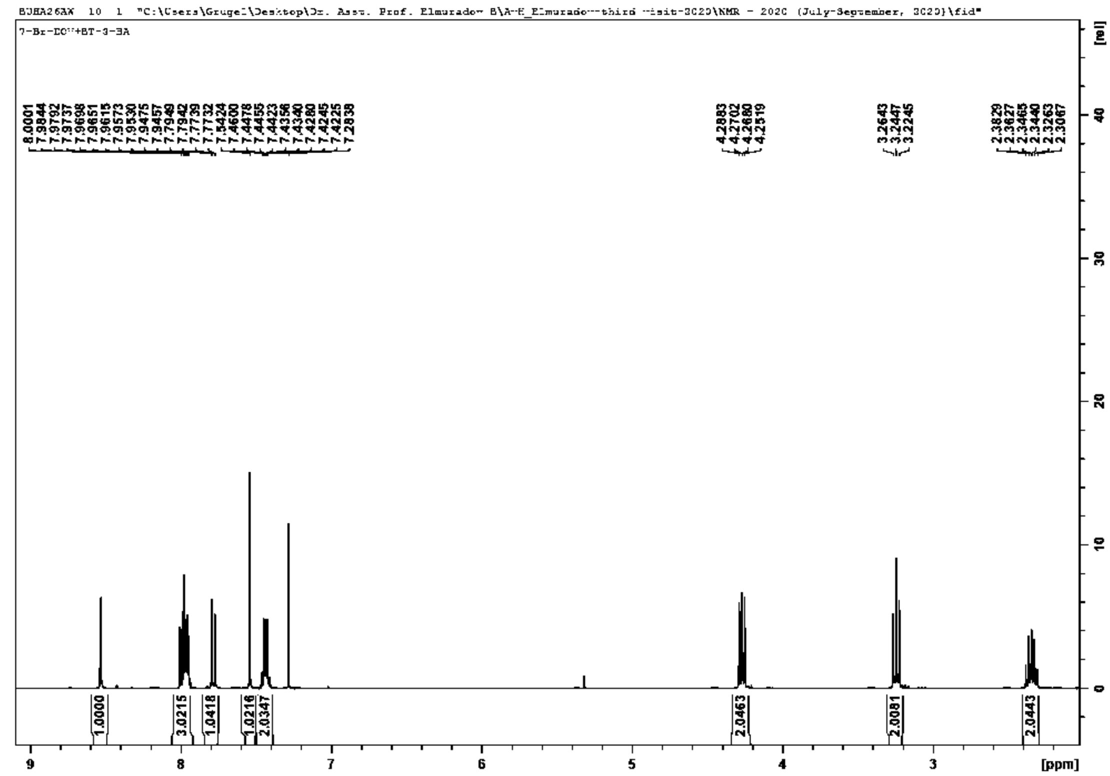

^{13}C{^{1}H} NMR (100.6 MHz, $CDCl_3$):

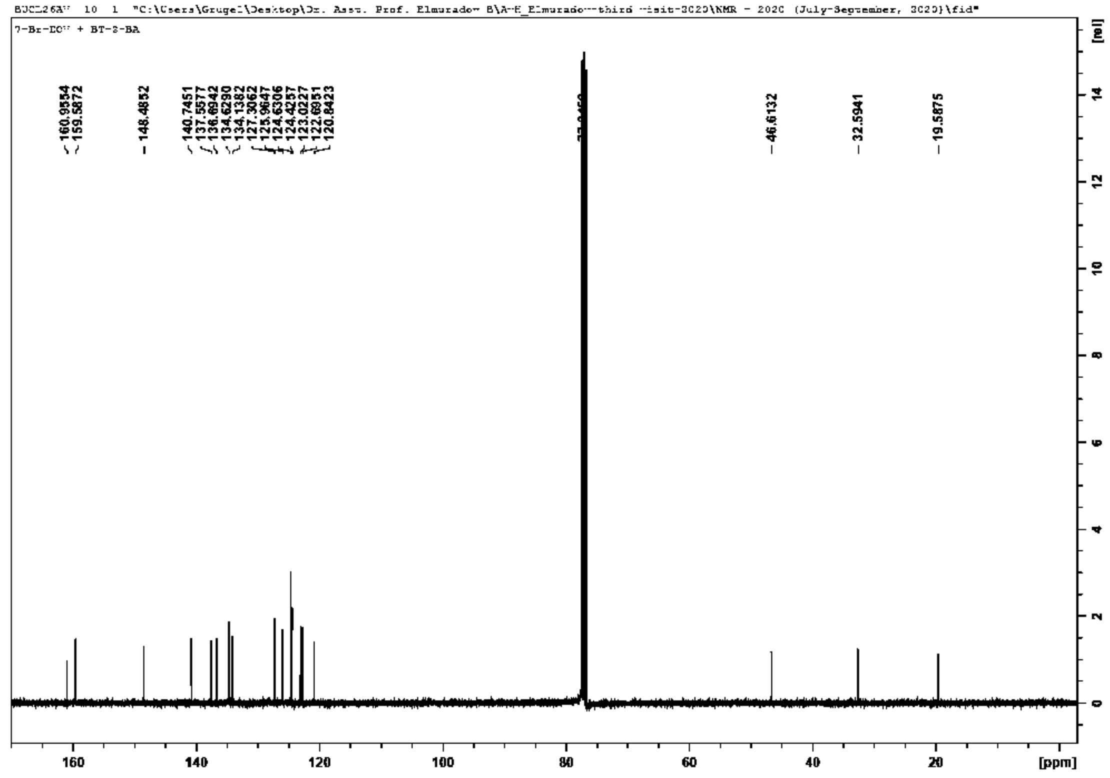

7-(Dibenzo[*b,d*]thiophen-4-yl)-2,3-dihydropyrrolo[2,1-*b*]quinazolin-9(1*H*)-one (11)

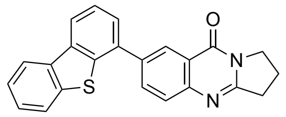

11

^{1}H NMR (400 MHz, $CDCl_3$):

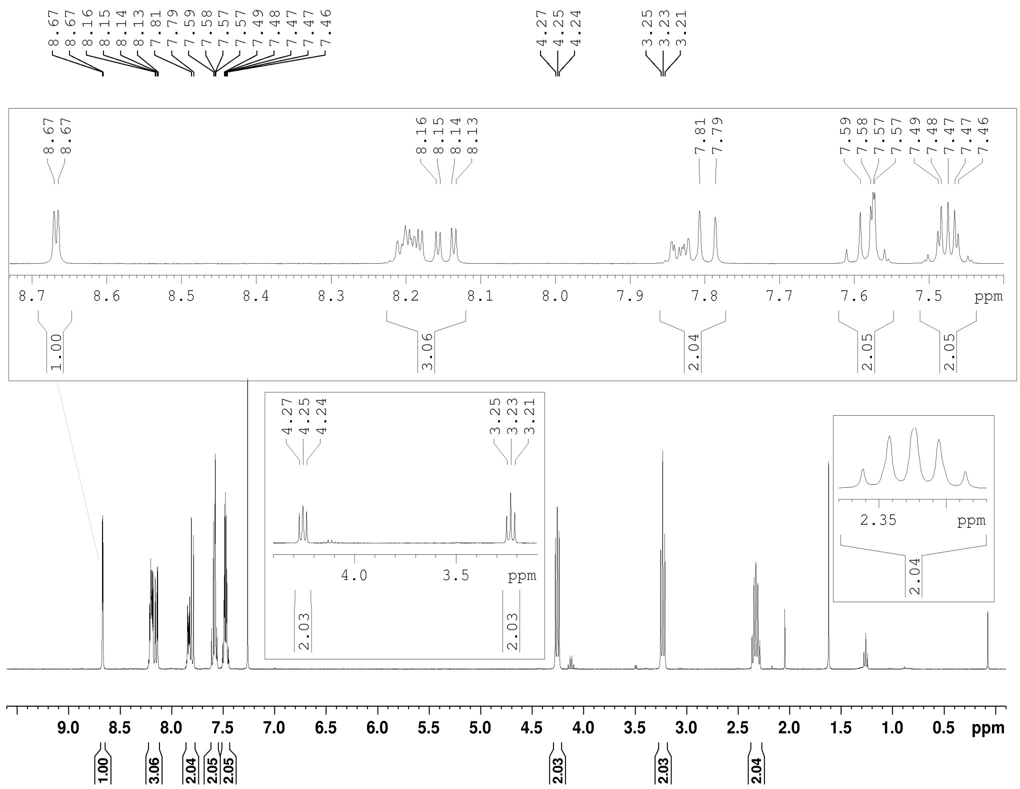

^{13}C{^{1}H} NMR (100.6 MHz, $CDCl_3$):

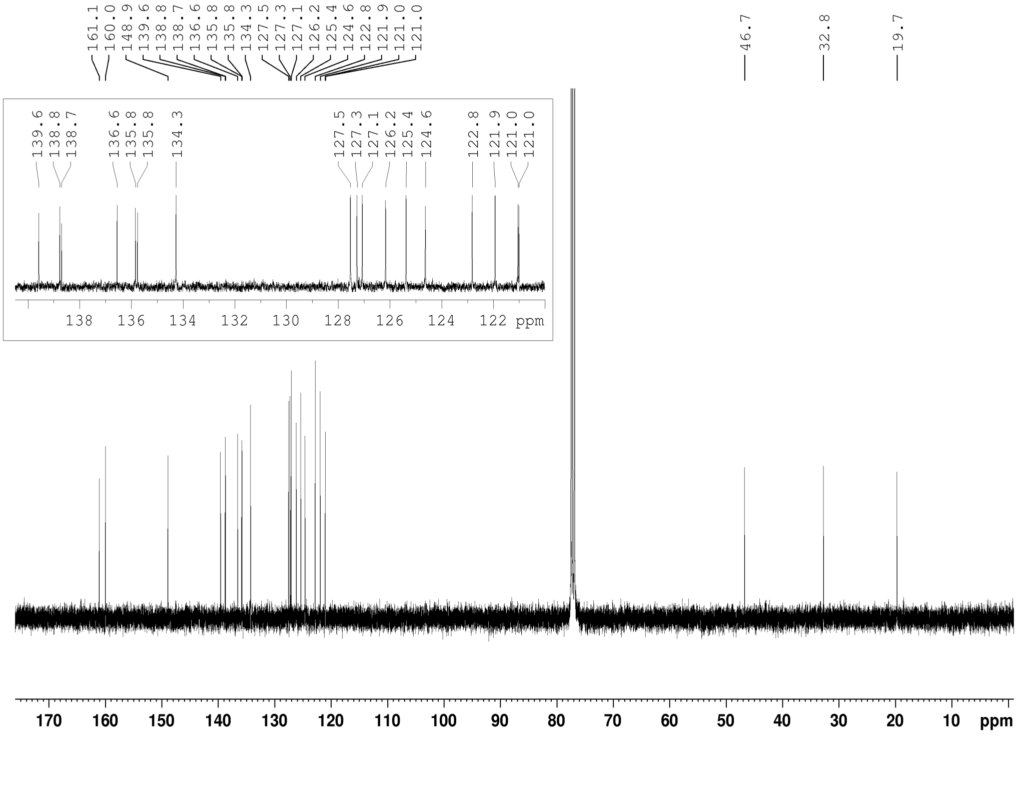

5,7-Diphenyl-2,3-dihydropyrrolo[2,1-*b*]quinazolin-9(1*H*)-one (12)

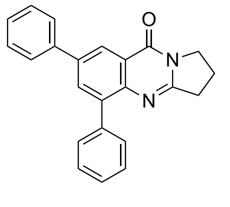

12

^{1}H NMR (400 MHz, $CDCl_3$):

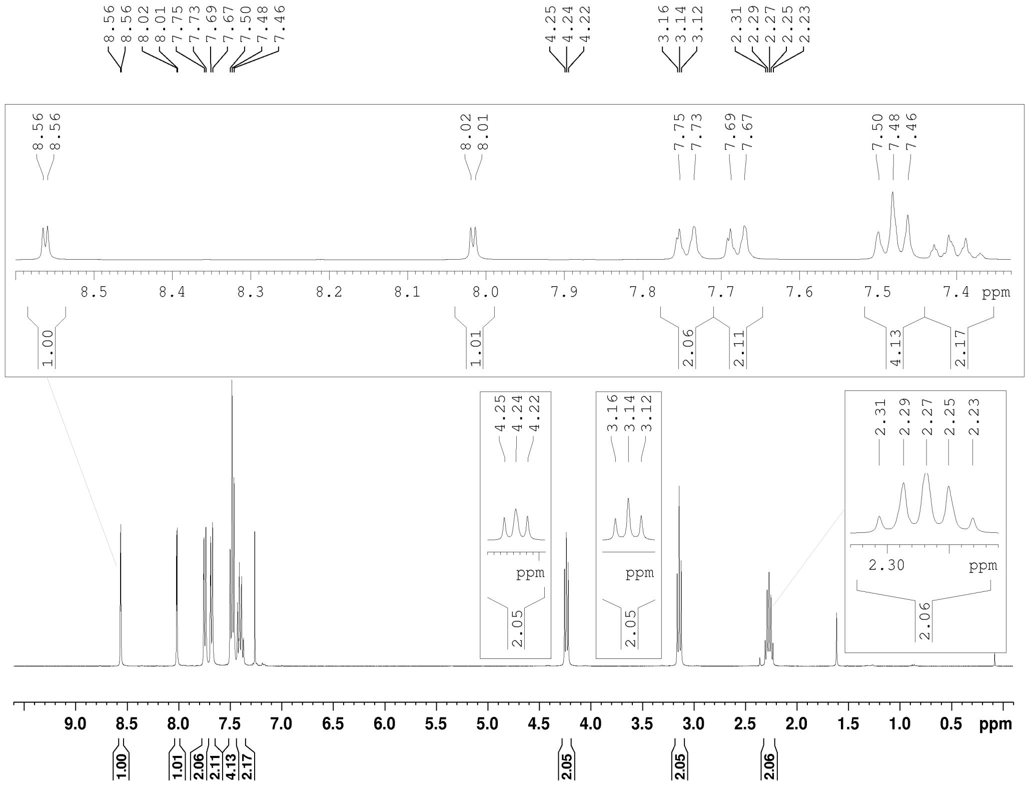

$^{13}C\{^{1}H\}$ NMR (100.6 MHz, $CDCl_3$):

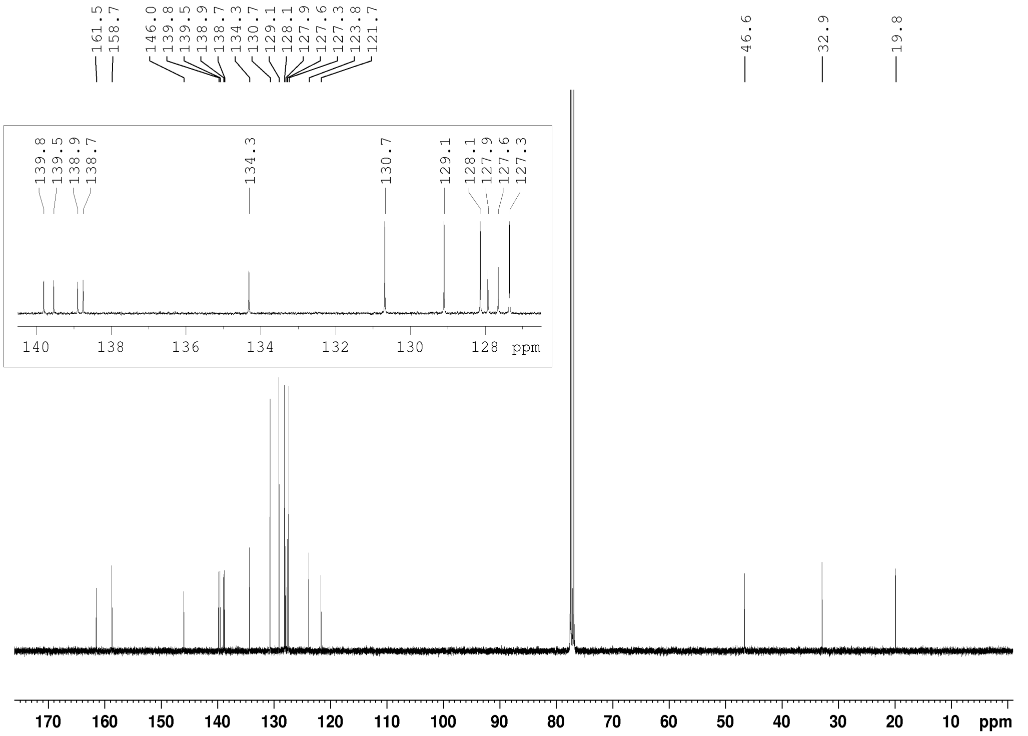

5,7-Bis(3,4,5-trimethoxyphenyl)-2,3-dihydropyrrolo[2,1-*b*]quinazolin-9(1*H*)-one (13)

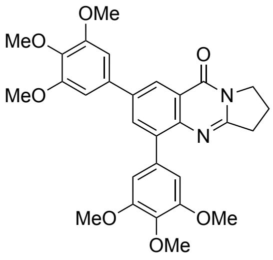

13

^{1}H NMR (400 MHz, $CDCl_3$):

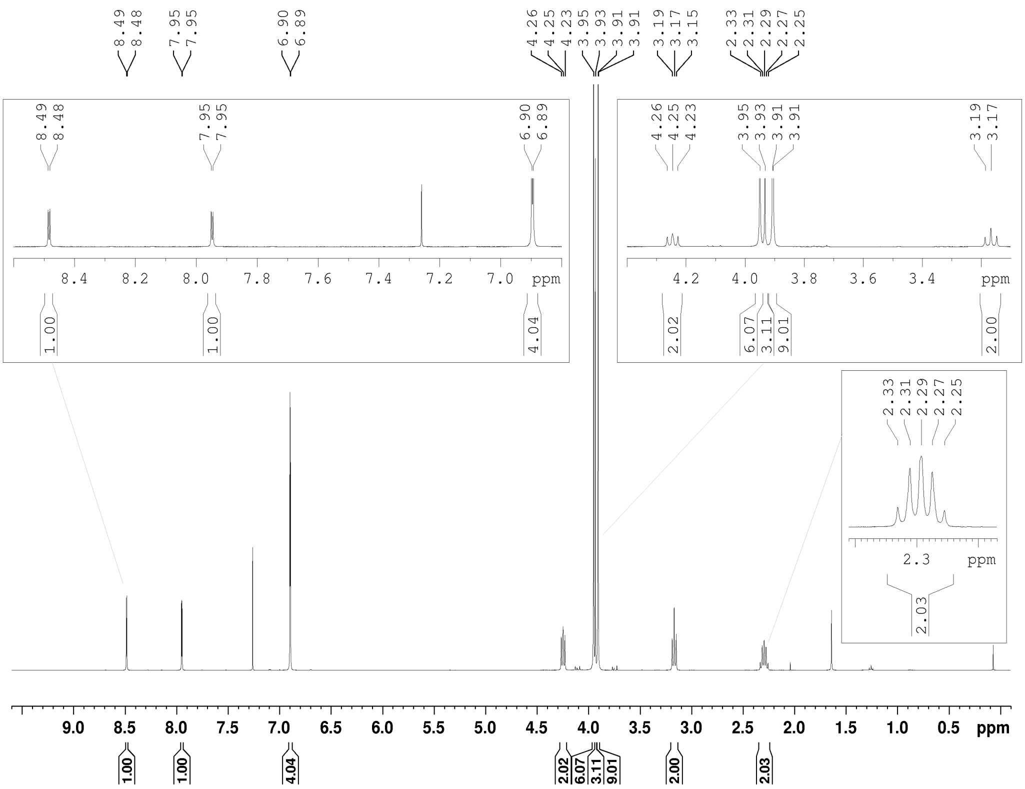

^{13}C{^{1}H} NMR (100.6 MHz, $CDCl_3$):

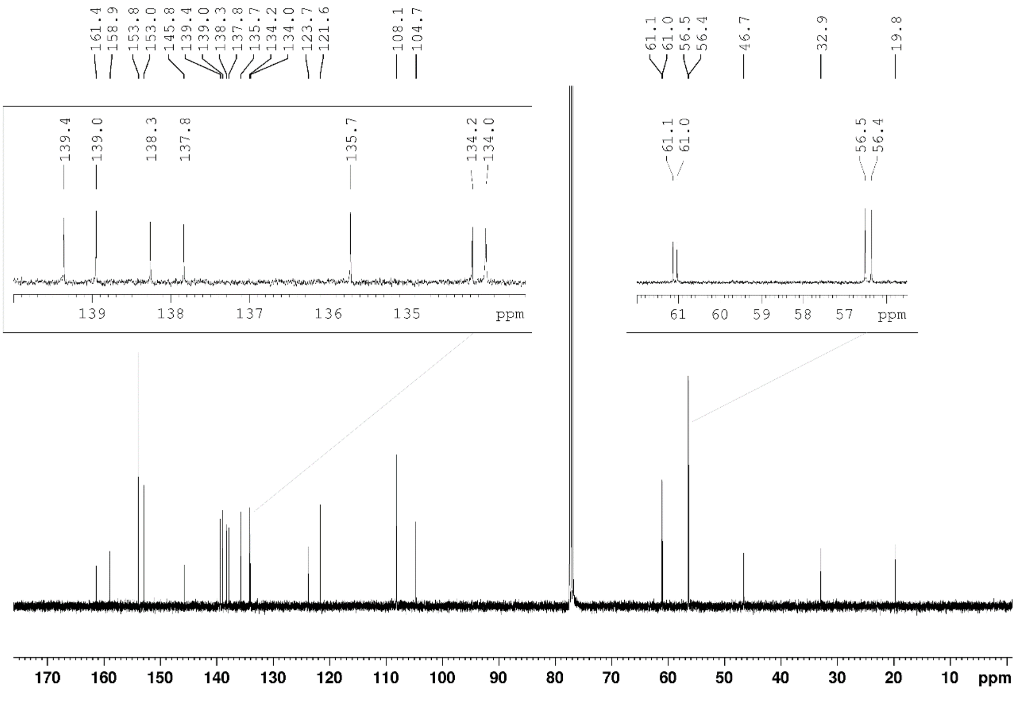

4,4'-(9-oxo-1,2,3,9-tetrahydropyrrolo[2,1-*b*]quinazoline-5,7-diyl)dibenzonitrile (14)

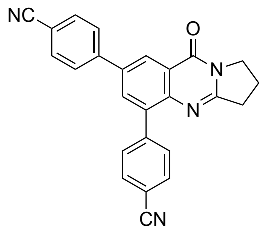

14

^{1}H NMR (400 MHz, $CDCl_3$):

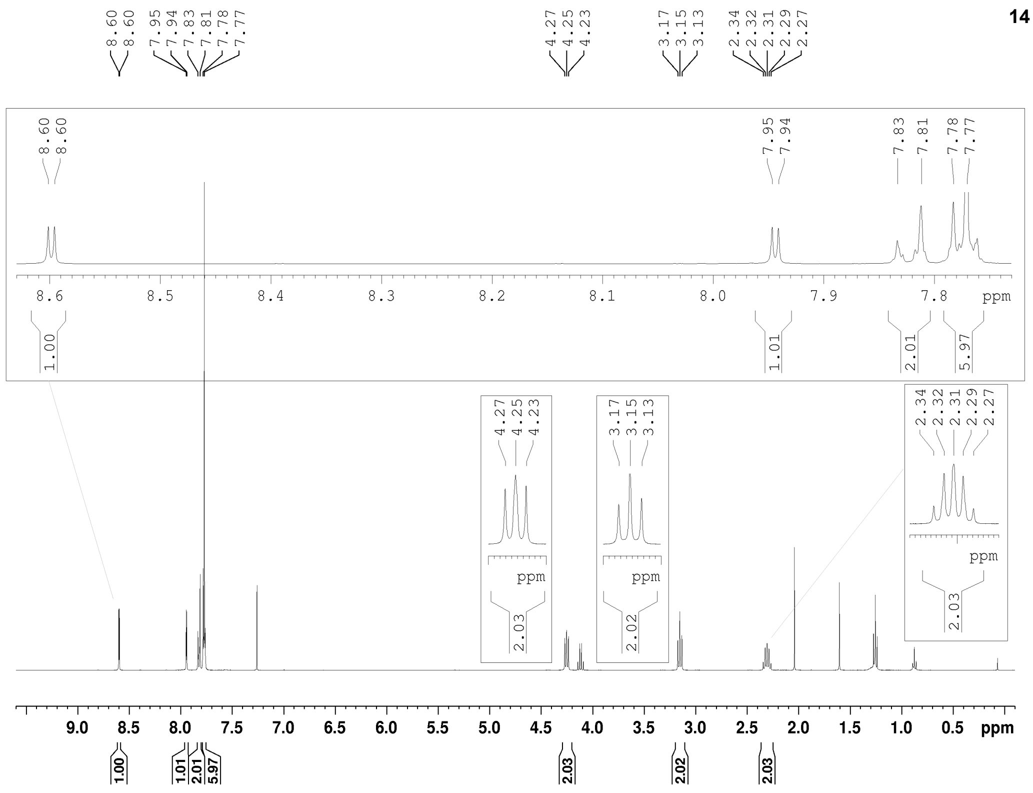

^{13}C{^{1}H} NMR (100.6 MHz, $CDCl_3$):

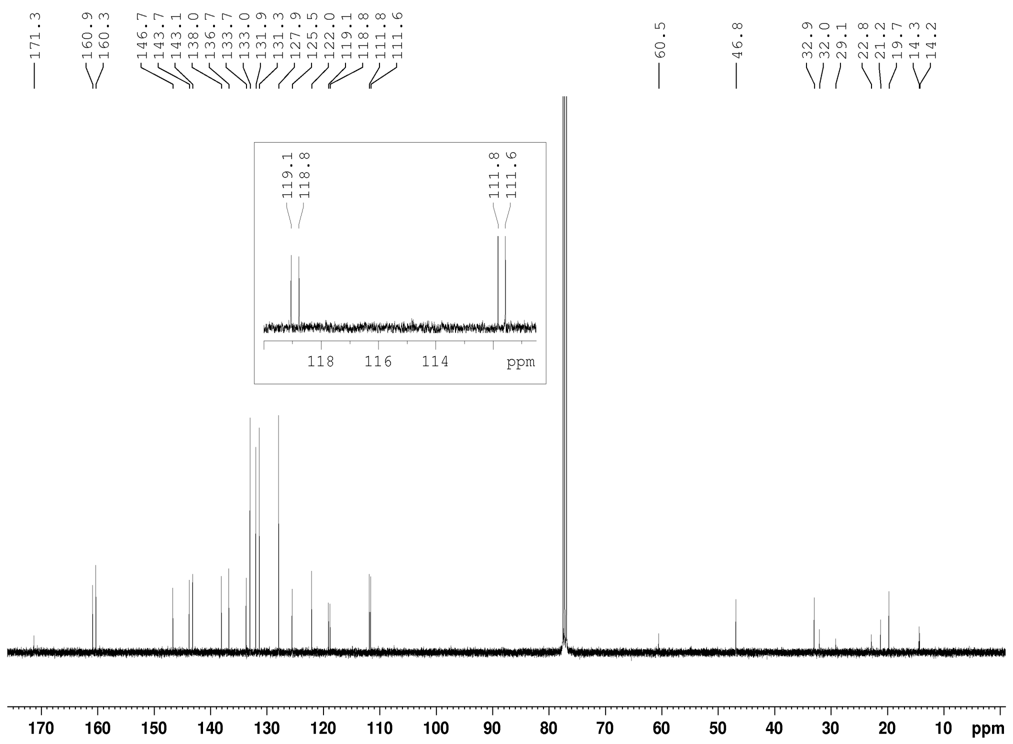

5,7-Bis(dibenzo[*b*,*d*]thiophen-4-yl)-2,3-dihydropyrrolo[2,1-*b*]quinazolin-9(1*H*)-one (15)

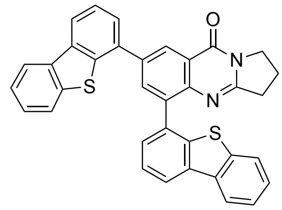

15

^{1}H NMR (400 MHz, $CDCl_3$):

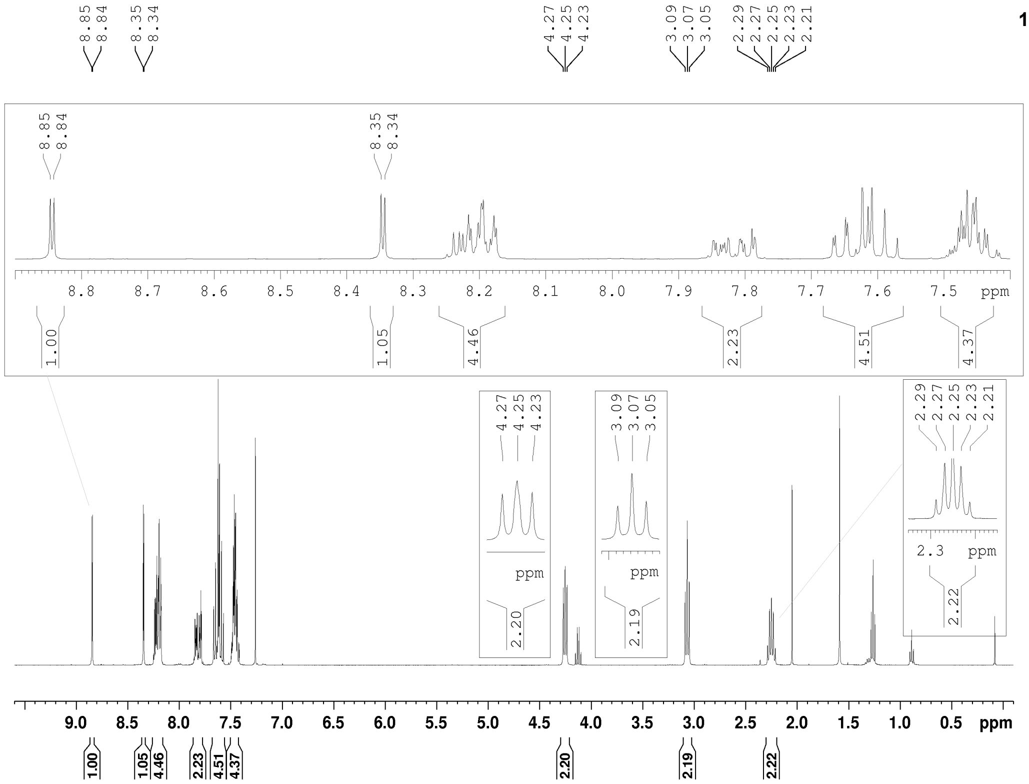

$^{13}C\{^1H\}$ NMR (100.6 MHz, $CDCl_3$):

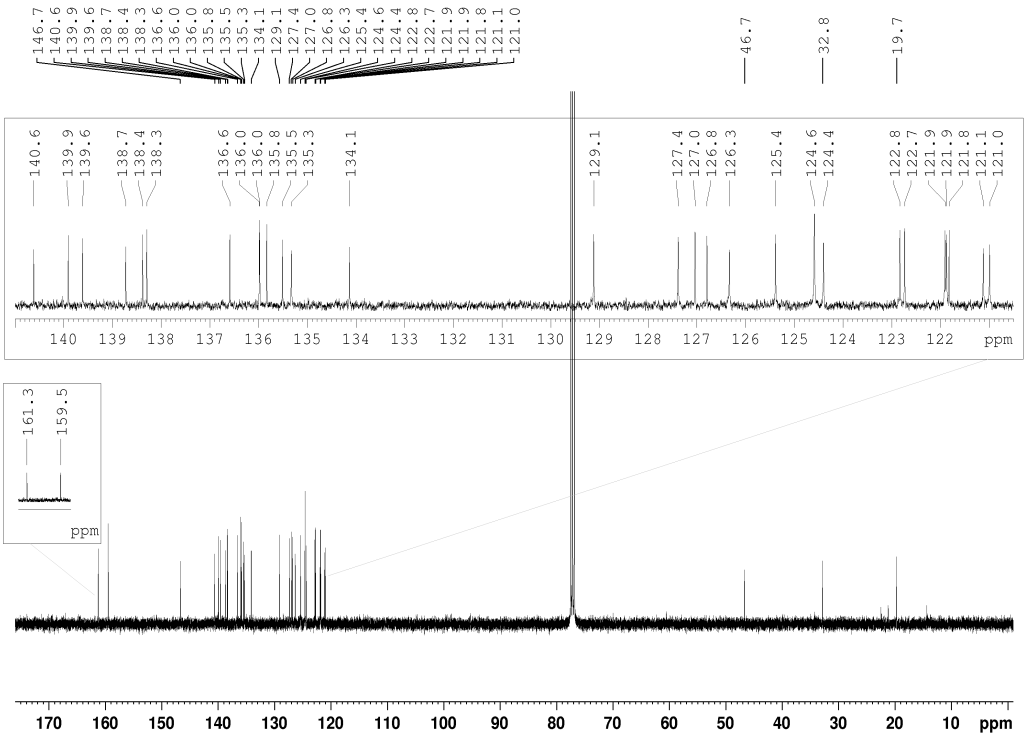

5-Bromo-7-(quinolin-8-yl)-2,3-dihydropyrrolo[2,1-*b*]quinazolin-9(1*H*)-one (16)

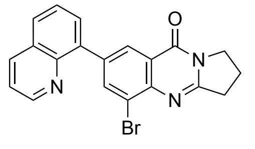

16

^{1}H NMR (500 MHz, $CDCl_3$):

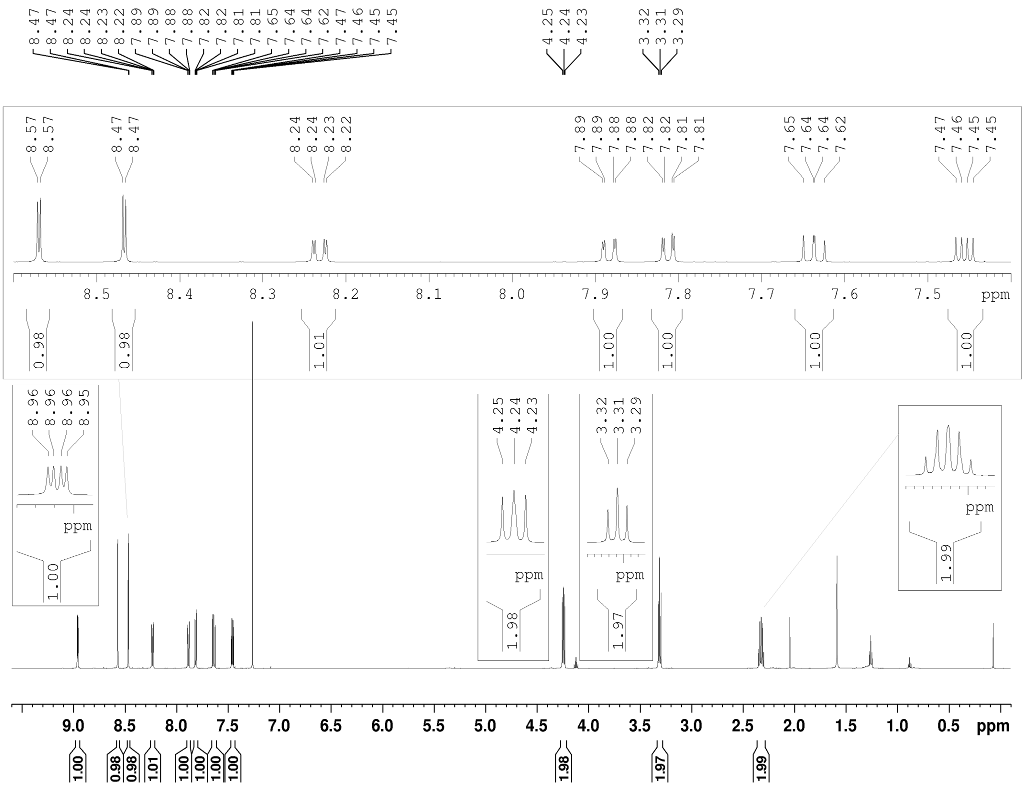

^{13}C{^{1}H} NMR (125 MHz, $CDCl_3$):

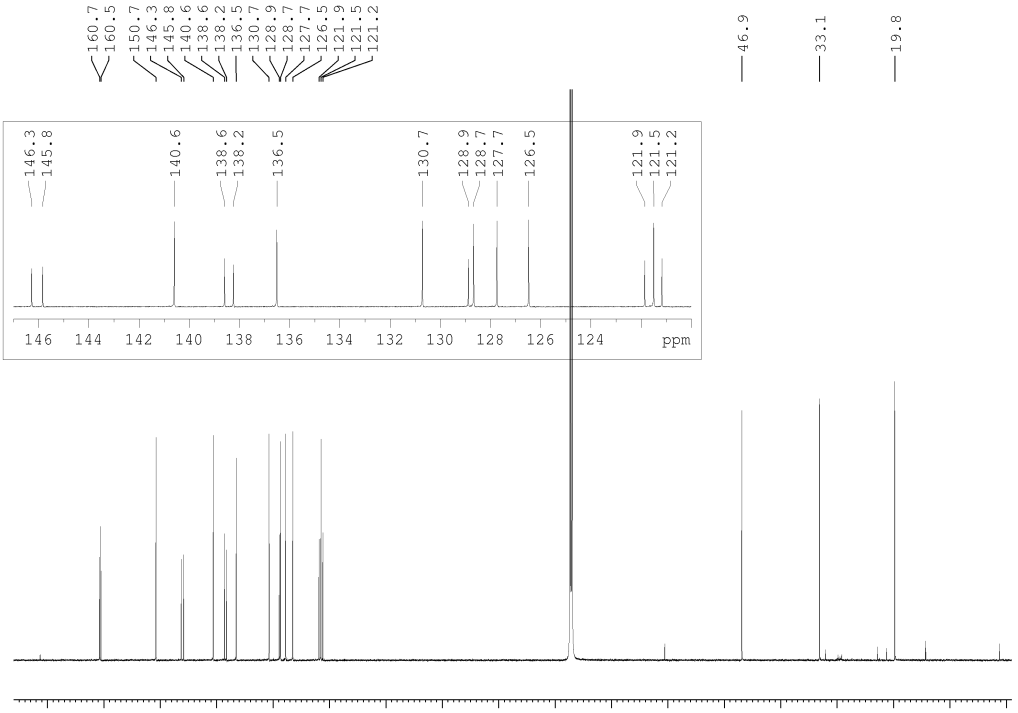

2-Phenyl-6,7,8,9-tetrahydro-11*H*-pyrido[2,1-*b*]quinazolin-11-one (17)

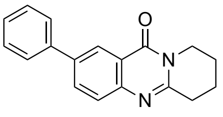

17

^{1}H NMR (400 MHz, CDCl$_3$):

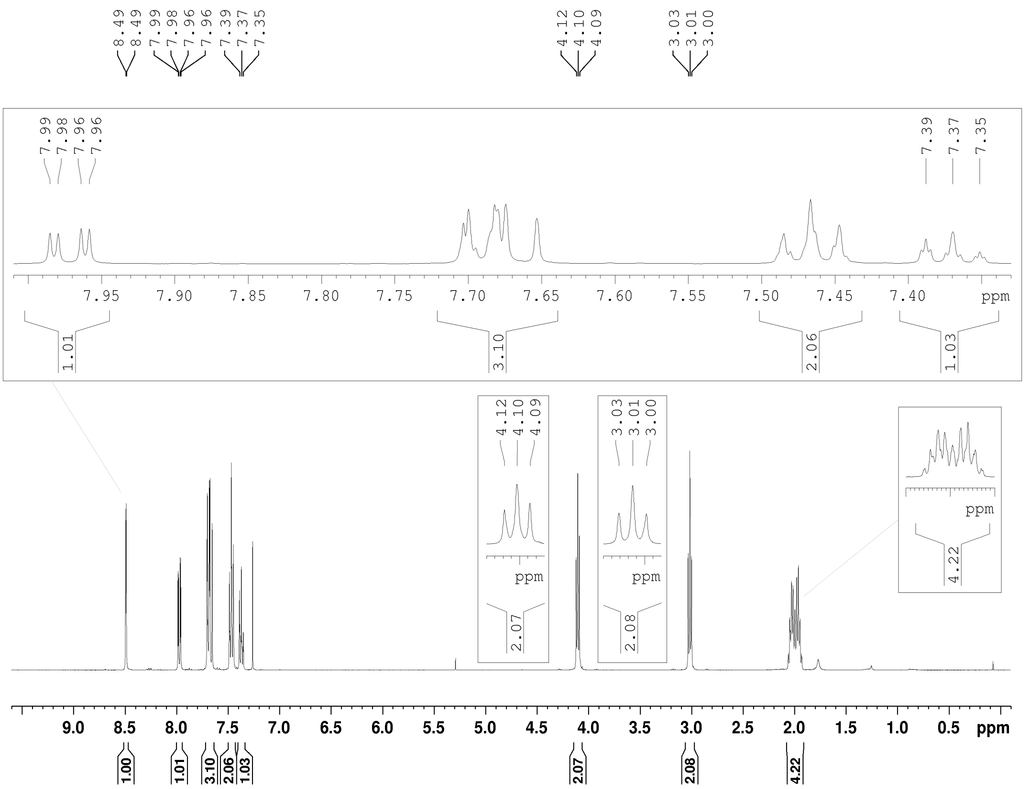

^{13}C{^{1}H} NMR (100.6 MHz, CDCl$_3$):

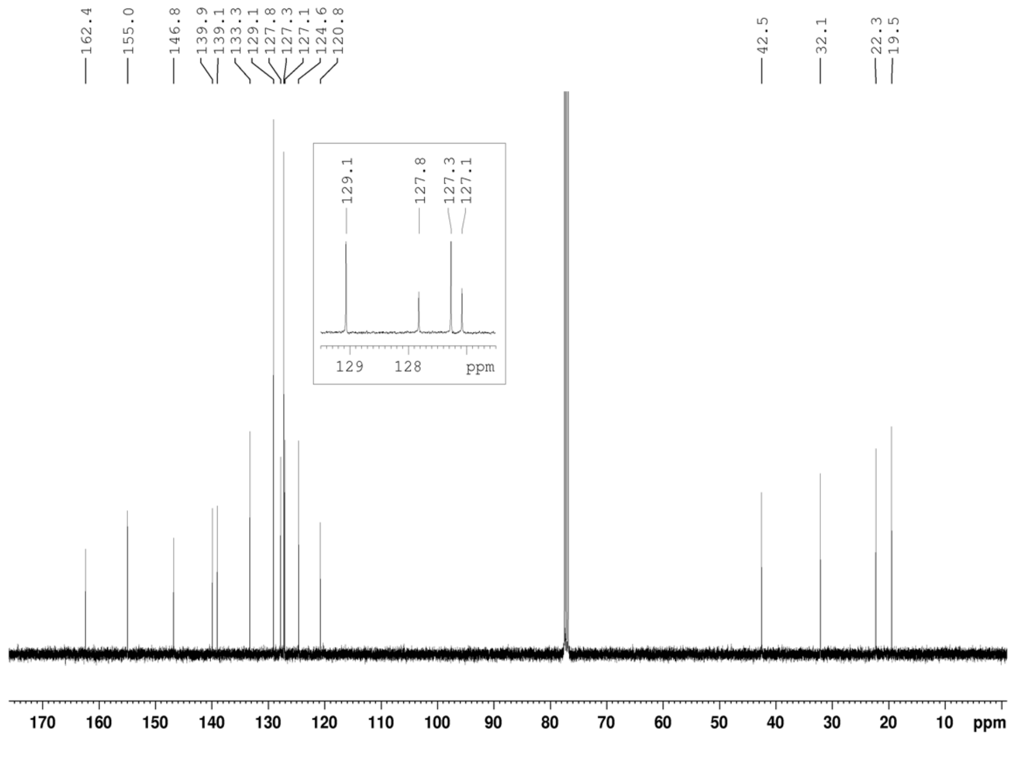

4-(11-Oxo-6,8,9,11-tetrahydro-7*H*-pyrido[2,1-*b*]quinazolin-2-yl)benzonitrile (18)

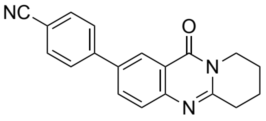

18

^{1}H NMR (400 MHz, $CDCl_3$):

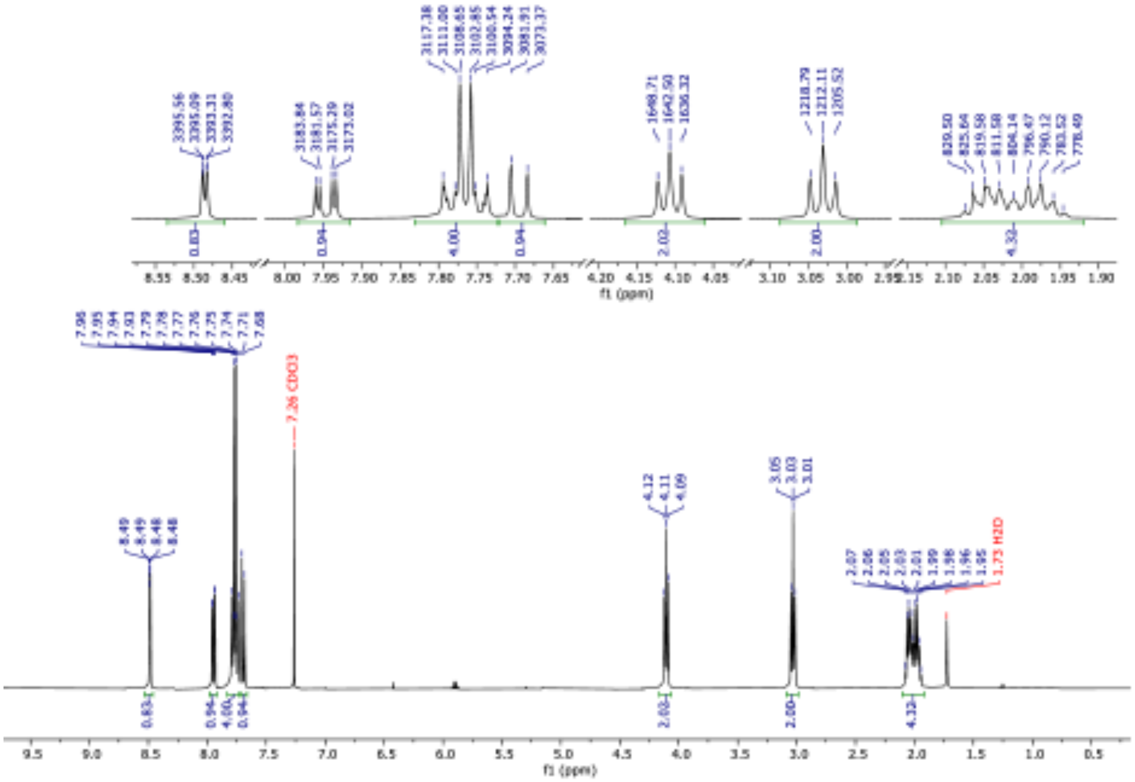

^{13}C{^{1}H} NMR (100.6 MHz, $CDCl_3$):

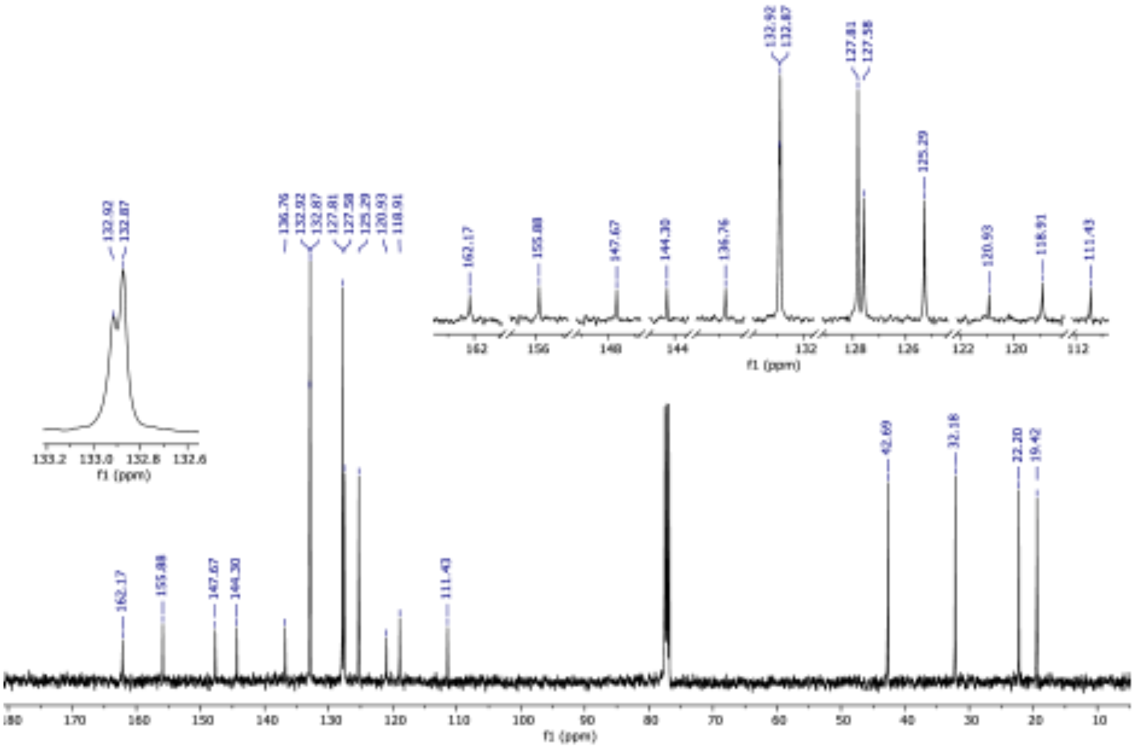

2-(3,4,5-Trimethoxyphenyl)-6,7,8,9-tetrahydro-11*H*-pyrido[2,1-*b*]quinazolin-11-one (19)

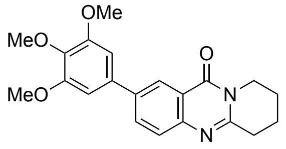

19

^{1}H NMR (400 MHz, $CDCl_3$):

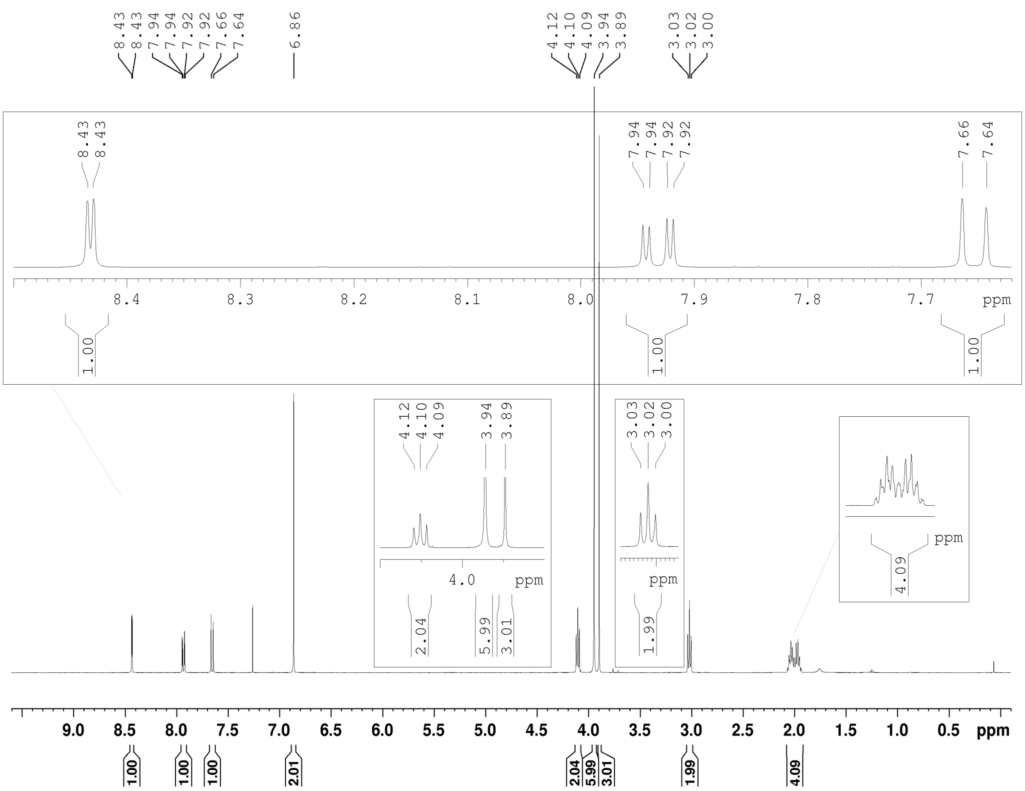

^{13}C{^{1}H} NMR (100.6 MHz, $CDCl_3$):

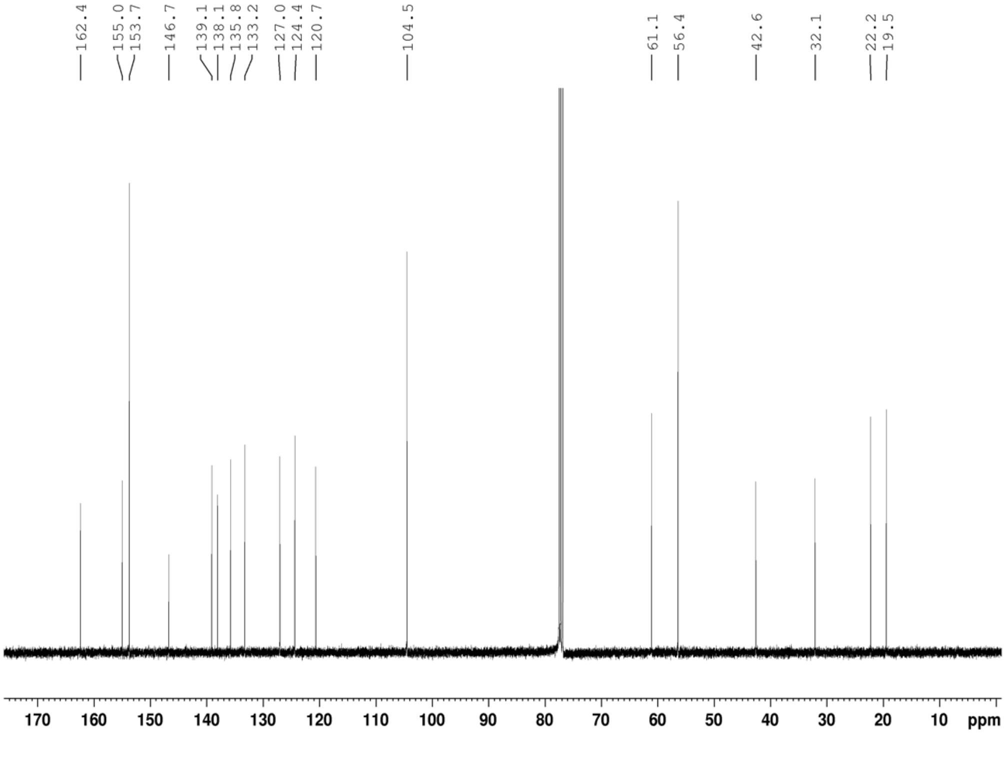

2-(Benzo[*b*]thiophen-3-yl)-6,7,8,9-tetrahydro-11*H*-pyrido[2,1-*b*]quinazolin-11-one (20)

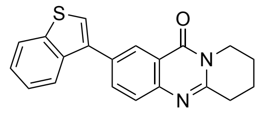

20

^{1}H NMR (400 MHz, $CDCl_3$):

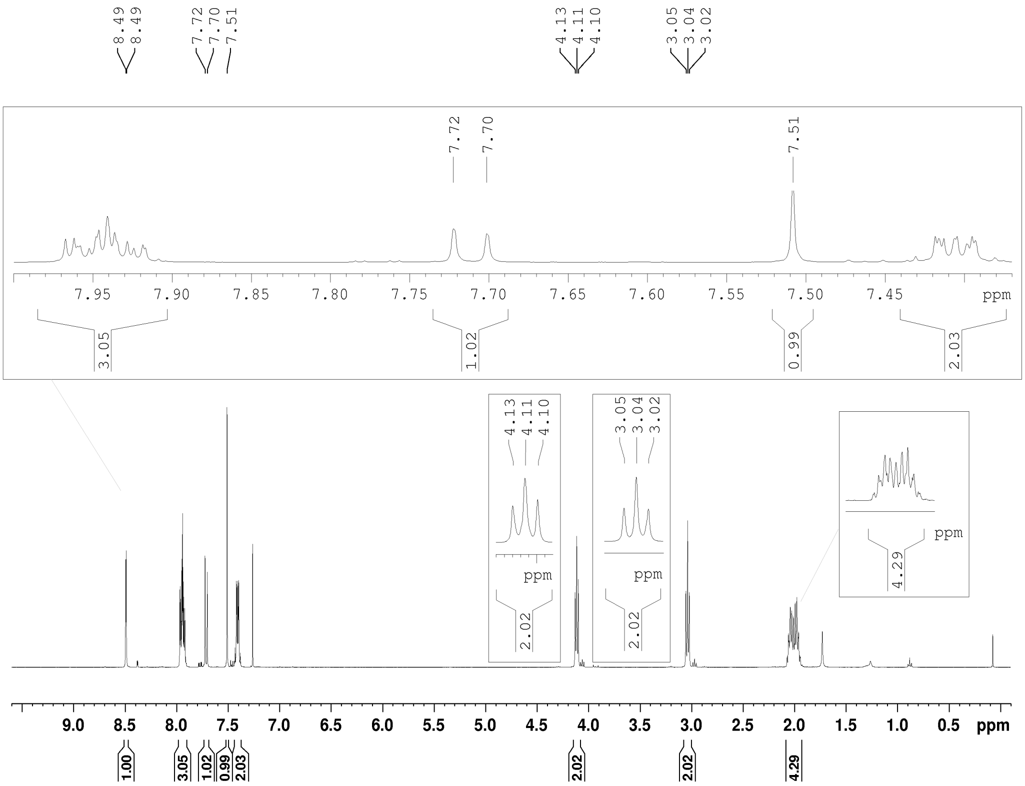

^{13}C{^{1}H} NMR (100.6 MHz, $CDCl_3$):

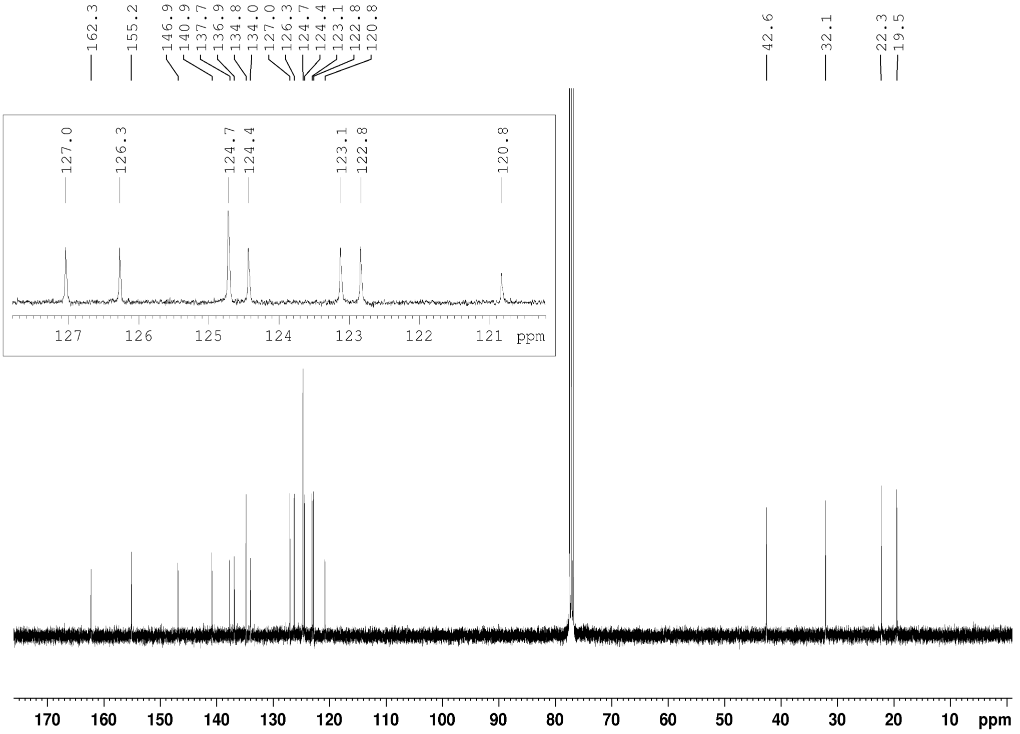

2-(Dibenzo[*b,d*]thiophen-4-yl)-6,7,8,9-tetrahydro-11*H*-pyrido[2,1-*b*]quinazolin-11-one (21)

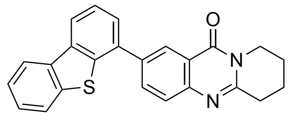

21

^{1}H NMR (400 MHz, $CDCl_3$):

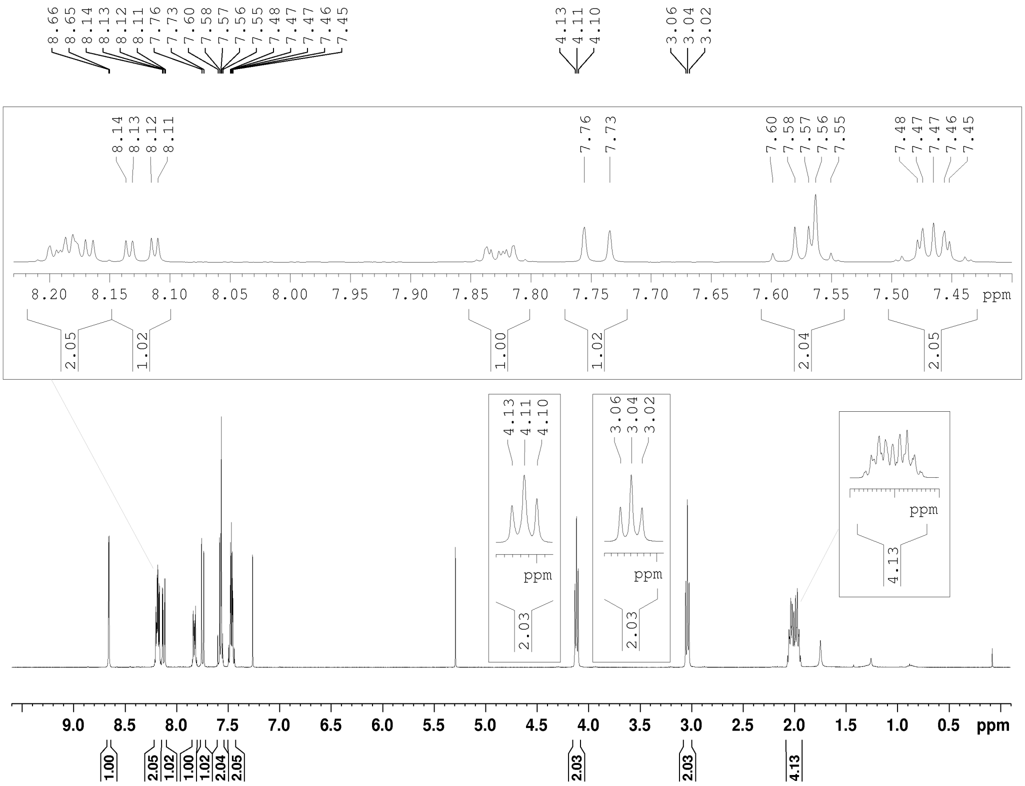

^{13}C{^{1}H} NMR (100.6 MHz, $CDCl_3$):

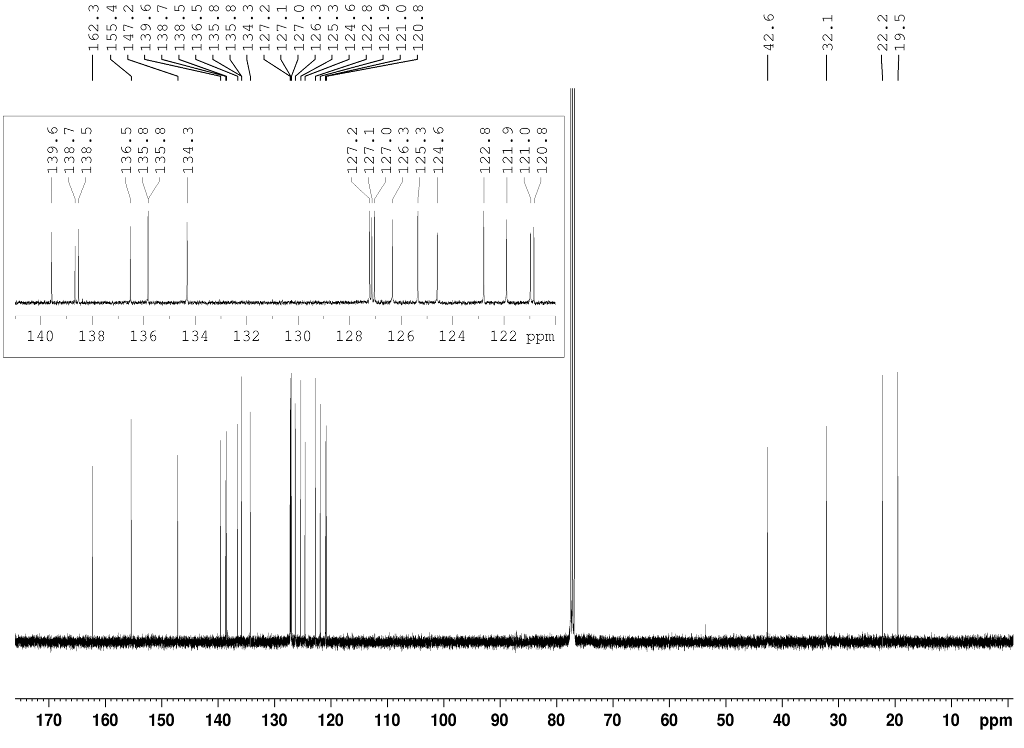

Supplement: Supplementary file 1 — Supporting Information [file OPEN-13-e202400197-s001.pdf]
